# Identification of a TLR2 Inhibiting Wheat Hydrolysate

**DOI:** 10.1002/mnfr.201800716

**Published:** 2018-11-02

**Authors:** Mensiena B. G. Kiewiet, Renske Dekkers, Martine P. van Gool, Laurien H. Ulfman, Andre Groeneveld, Marijke M. Faas, Paul de Vos

**Affiliations:** ^1^ Immunoendocrinology Division of Medical Biology Department of Pathology and Medical Biology University of Groningen University Medical Center Groningen Hanzeplein 1 9700 RB Groningen The Netherlands; ^2^ FrieslandCampina Stationsplein 4 3818 LE Amersfoort The Netherlands; ^3^ Department of Obstetrics and Gynecology University of Groningen University Medical Center Groningen 9713 GZ Groningen The Netherlands

**Keywords:** bioactive peptides, inhibition, mucositis, toll‐like receptor 2, wheat hydrolysates

## Abstract

**Scope:**

Wheat hydrolysates are used in medical nutrition to provide undernourished patients a readily digestible protein source, for instance to recover from chemotherapy‐induced intestinal mucosal inflammation. Since many hydrolysates of different sources can modulate the immune system, likely via Toll‐like receptors (TLRs), it is hypothesized that also wheat hydrolysates might interact with TLR signaling, which could be a way to prevent intestinal inflammation and damage.

**Methods and results:**

The capacity of three wheat hydrolysates to modulate immunity by interfering with TLR signaling is determined. All wheat hydrolysates have TLR modulating effects but only one has strong TLR2 inhibiting effects, attenuating both TLR2/1 and TLR2/6 signaling in a reporter cell system. This is likely induced by direct TLR2‐ectodomain binding, as confirmed by ELISA. Furthermore, this TLR2 blocking hydrolysate reduces IL‐6 production in human dendritic cells. Application of reversed‐phase–ultra HPLC combined with MS reveals that the presence of peptide WQIPEQSR is associated with the observed TLR2 inhibiting capacity.

**Conclusion:**

The study demonstrates TLR2‐inhibiting capacities of a wheat hydrolysate. The findings provide a good start for further research to investigate whether this hydrolysate might contribute to the management of intestinal mucosal inflammation in cancer patients receiving chemotherapy.

## 1 Introduction

Nutritional interventions with hydrolysates are used to prevent adverse clinical outcomes in undernourished patients.^1^ About 30% of hospitalized patients are at risk for undernutrition, which leads to prolonged hospital stay, increased readmissions, and increased mortality.^2^, ^3^ This malnutrition is not always caused by decreased appetite, but also by malabsorption due to intestinal issues, such as inflammation of the intestinal mucosal membrane, also referred to as intestinal mucositis.^4^ This may be the consequence of disease, or develop as a side effect of pharmaceutical intervention. For example, the majority of cancer patients treated with chemotherapeutics develop gastrointestinal mucositis, resulting in malnutrition.^5^

Clinical nutrition to treat undernourished patients with intestinal issues contains high amounts of proteins,^6^ that are hydrolyzed to facilitate uptake in the damaged intestine.^7^ Wheat hydrolysates are of special interest for this type of nutritional products, due to their relatively high peptide‐bound glutamine content,^8^ compared to, for example, cow's milk hydrolysates. Glutamine supplementation was found to decrease the length of a hospital stay, complications, and mortality in critically ill patients.^9^

Besides their nutritional value, protein hydrolysates have been recognized to actively modulate the immune system.^10^ Multiple hydrolysates were found to induce many different immune effects, including a range of anti‐inflammatory effects.^11^, ^12^, ^13^, ^14^ These effects could be induced by interaction of bioactive peptides in the hydrolysates with Toll‐like receptors (TLRs), since we previously showed immune effects of cow's milk hydrolysates via TLR activation or inhibition.^15^ Some studies on wheat hydrolysates also demonstrate similar immune effects.^16^ Thus, we hypothesized that wheat hydrolysates could potentially modulate immunity via TLR signaling.

Immunomodulating effects of wheat hydrolysates may be of special interest in treatment of mucositis induced by chemotherapy. Mucositis is a common side effects of chemotherapy.^17^ Currently, there is no cure for this side effect of chemotherapy but it is known that TLR signaling is involved.^18^ It starts with injury of epithelial and underlying cells by the chemotherapeutic drug, which results in the release of damage associated molecular patterns (DAMPs) by dying cells. These DAMPs stimulate TLRs on neighboring epithelial cells and immune cells, and induce immune activation^19^ causing mucosal damage and barrier dysfunction.^20^ Especially TLR2, TLR4, and TLR9 activation has been found to play a role in intestinal mucositis development, and a deficiency in these receptors was found to be protective against mucositis.^21^, ^22^

Inhibition of TLR2 by food components in the lumen of the gut has been shown to be an efficacious approach to prevent mucositis.^23^ Wheat hydrolysates may inhibit TLR2 on epithelial cells or, in case of barrier disruption, inhibit intestinal immune cell function in a similar way. Dendritic cells are of special interest in this setting. Specific subtypes are located within the epithelial layer,^24^ and can therefore come into contact with dietary molecules like hydrolysates in the lumen of the gut, but also mucositis inducing DAMPs. Via TLRs, these compounds can regulate their maturation status and cytokine production.^25^ The immune status of dendritic cells is suggested to play an important role in the regulation of the intestinal inflammation cascade,^26^ which makes them interesting targets for anti‐inflammatory food components like hydrolysates.

Here, we studied the possible inhibiting effects of three hydrolysates on TLR2, TLR4, and TLR9 activation, which might contain TLR modulating proteins and peptides. We further studied the effects of the most potential hydrolysate in more detail by investigating the inhibiting effects of the TLR2/1 and TLR2/6 dimers and by studying effects on dendritic cell cytokine production. We also identified the bioactive fraction within the hydrolysate, and identified possible bioactive peptides from this fraction. Our technology platform containing TLR expressing reporter cells might lead to identification of specific wheat hydrolysates that not only serve as a nutrient source, but can also serve as bioactive food component.

## 2 Experimental Section

### 2.1 Tested Materials

Wheat hydrolysates Wheat 1 (Hyvital Wheat Glutamine PU), Wheat 2 (Hyvital Wheat Glutamine PA), and Wheat 3 (Hyvital Wheat Glutamine PN) were obtained from FrieslandCampina (Amersfoort, the Netherlands). During the production, the hydrolysates were hydrolyzed to a final degree of hydrolysis (DH) of approximately 10–12%. An example of a typical molecular weight distribution (MWD) of the peptides of Hyvital Glutamine PU obtained by fingerprint analysis and size exclusion chromatography is shown in Supporting Information File S1, and the MWD of all tested hydrolysates are summarized in **Table** 1. The amount of free amino acids in the hydrolysates was less than 2% (w/w). The amino acid profile of the hydrolysates is listed in **Table** 2. The protein content (TN*5.79) of Hyvital Wheat Glutamine was between 76% and 79%. The samples were tested for endotoxins by using the Limulus amebocyte lysate assay (LAL) according to the manufacturer's instructions (ThermoFisher Scientific, Waltham, US). Endotoxin concentrations in the samples had no significant activating effect on the cells applied.

**Table 1:** Overview of the molecular weight distribution of the hydrolysates studied

Samples	Source	Molecular weight distribution [%]					
		>10 000 Da	10 000–5000 Da	5000–2000 Da	2000–1000 Da	1000–500 Da	<500 Da
Wheat 1	Wheat gluten	17	15	20	14	12	22
Wheat 2	Wheat gluten	1	1	5	9	18	66
Wheat 3	Wheat gluten	1	1	4	7	13	74

**Table 2:** Amino acid profiles of the hydrolysates studied

Amino acids [mg g^–1^]	Hydrolysate		
	Wheat 1	Wheat 2	Wheat 3
Alanine	19	19	17
Arginine	27	24	22
Aspartic acid	24	22	19
Cysteine	21	17	10
Glutamic acid (including glutamine)	294	342	328
Glycine	26	28	28
Histidine	14	14	8
Isoleucine	32	28	24
Leucine	54	54	47
Lysine	12	12	9
Methionine	11	9	8
Phenylalanine	42	46	42
Proline	104	114	115
Serine	31	38	31
Threonine	19	20	15
Tryptophan	5	5	2
Tyrosine	26	24	22
Valine	31	31	26
Branch chain amino acids	117	113	96

### 2.2 Dosage Information

The goal of the performed experiments was to show whether hydrolysates can interact with TLRs, and therefore, a concentration was chosen that was optimal for our HEK TLR reporter cell lines. The used concentration of 2 mg mL^–1^ was based on our previous experience with testing dietary molecules in HEK reporter cell assays, and it was found that the milligram range was the concentration at which the HEK cells responded adequately and provided enough response to be able to measure meaningful differences between samples.^15^, ^27^ Furthermore, other in vitro experiments studying hydrolysates described in literature also used concentrations in the milligram range.^28^

Cells were inspected visually, and no toxic effects were observed after incubation with the hydrolysates. The studied hydrolysates are intended to be used in clinical nutrition during a limited time of illness. In these products, the protein concentration is relatively high, since a higher protein intake (up to 1.5 g kg^–1^ d^–1^, for example, 20 g of protein taken at once) is recommended in patients with intestinal inflammation.^29^ During the intake of this significant amount of protein at once, it is expected that a protein concentration of 2 mg mL^–1^ can be reached in the lumen of the gut. The protein concentrations used in the in vitro setting are therefore assumed to be physiologically relevant.

### 2.3 Inhibition Assay Using HEK‐XBlue‐hTLR2, HEK‐XBlue‐hTLR4, and HEK‐XBlue‐hTLR29 Reporter Cells

To test whether the wheat hydrolysates are able to inhibit TLR2, TLR4, and TLR9 activation induced by known ligands, the samples were tested on a HEK‐XBlue‐hTLR2, HEK‐XBlue‐hTLR24, and HEK‐XBlue‐hTLR29 (Invivogen, Toulouse, France) reporter cell assay. To quantify TLR activation, the cell line contains both a TLR construct and a construct for secreted embryonic alkaline phosphatase (SEAP), which was coupled to the nuclear factor κB/activating protein‐1 (NF‐κB/AP‐1) promoter. NF‐κB/AP‐1 is a known downstream target of TLR receptors.^30^, ^31^

Cell culturing and the cell assay were performed following the manufacturer's instructions, and as described before.^30^ This method is also described in detail in the Supporting Information File S2. Since the most striking inhibiting effect was observed by 2 mg mL^–1^ Wheat 1 on TLR2, this effect was further investigated. The dose dependency of the overall TLR2 inhibition was tested by stimulating HEK‐XBlue‐hTLR2 cells with graded concentrations of wheat hydrolysate, and HKLM. The TLR2 receptor forms heterodimers with TLR1 and TLR6 in order to recognize a broader range of ligands^32^ and stimulate different immune pathways.^33^, ^34^ In order to investigate whether TLR2 inhibition is mediated via the TLR2/TLR1 or TLR2/TLR6 heterodimer, the above experiment was repeated using the TLR2/TLR1 specific tri‐acetylated lipopeptide P3CSK4 (25 ng mL^–1^)^35^ and the TLR2/TLR6 specific di‐acetylated lipopeptide FSL‐1 (25 ng mL^–1^)^36^ as activating ligands instead of HKLM.

### 2.4 TLR2 ELISA

HEK293T cells expressing TLR2‐ectodomain‐HA, which had previously been developed,^23^ were first expanded in T162 flasks with 50 µg mL^–1^ Blasticidin. TLR2‐ectodomain‐HA protein was immunoprecipitated as described before.^23^ TLR2 ELISA has been developed in our group before and has been performed as described.^23^ In short, ELISA plates (Corning, Tewksbury, MA, USA) were treated with 50 µL of 50 µg mL^–1^ poly‐l‐lysine for 1 h at 37 °C. Wells were washed once with 400 µL ELISA buffer (1 mm CaCl_2_ and 150 mm NaCl in 0.05 m Tris, pH 8.2). 1 mg mL^–1^ Wheat 1 in ELISA buffer was applied to the wells, and the plate was incubated for 4 h at 37 °C. Wells were then washed with ELISA buffer and blocked overnight with 3% milk powder (FrieslandCampina, Amersfoort, the Netherlands) in ELISA buffer (100 µL per well) at 4 °C. After washing the plate once with ELISA buffer, isolated TLR2‐ectodomain‐HA protein was applied to the wells at 10 µg per well and 1 µg per well and incubated for 3 h at 37 °C. As a negative control, 10 µg per well and 1 µg per well HA peptide was also applied to wells. After incubation, the plate was washed five times with ELISA buffer and incubated for 2 h at 37 °C with 50 µL mouse‐anti‐HA tag antibody (1:200 in 1:2 blocking buffer in ELISA buffer; Cell signaling, Danvers, MA, USA). After washing the plate again for five times, 50 µL goat‐anti‐mouse biotin antibody was applied to the wells for 1 h at 37 °C (1:500 in 1:2 blocking buffer in ELISA buffer; Southern Biotechnology, Birmingham, AL, USA). Then, wells were washed five times with ELISA buffer, and subsequently incubated for 1 h at 37 °C with streptavidin‐HRP antibody (1:1000 in 1:2 blocking buffer in ELISA buffer; Dako, Glostrup, Denmark). Finally, the plate was washed seven times with ELISA buffer, and incubated for 30 min at 37 °C with 100 µL TMB (Cell signaling, Danvers, MA, USA). The reaction was stopped by adding 100 µL stop solution (Cell signaling, Danvers, MA, USA). Absorbance (450 nm) was quantified using a VersaMax microplate reader (Molecular Devices GmbH, Biberach an der Riss, Germany) and SoftMax Pro Data Acquisition & Analysis Software.

### 2.5 Direct Stimulation of Dendritic Cells (DCs) with Wheat Hydrolysate

To investigate the direct effects of wheat hydrolysate on human DCs, cytokine production was measured after stimulation of immature DCs (MatTek Corporation, Ashland, MA, USA) with the wheat hydrolysate for 24 h. These cells are described to express TLR1, TLR2, TLR3, TLR4, TLR6, TLR7, and TLR9.

Stimulations were performed by seeding 6 × 10^4^ per well freshly thawed DCs in each well of a 96‐well plate (in 200 µL). Cells were precultured for 24 h as described in the manufacturer's instructions. Then, cells were exposed to 2 mg mL^–1^ hydrolysate and 10^7^ cells mL^–1^ HKLM, after which cells were incubated for 24 h (37 °C, 5% CO_2_). HKLM alone was used as a positive control, medium as a negative control. To assess the role of TLR2 inhibition in the effects observed, instead of the hydrolysate, cells were treated with 5 µg mL^–1^ TLR2 blocking antibody (PAb‐hTLR2, Invivogen, Toulouse, France) for 30 min, after which HKLM was added. Supernatant was collected and stored at –80 °C for cytokine measurements.

### 2.6 Assessment of Cytokine Expression

The levels of IL‐1β, IL‐1RA, IL‐10, IL‐12, IL‐6, IL‐8, MCP‐1/CCL2, MIP‐1α/CCL3, RANTES/CCL5, TNF‐α, and TSLP in the DC supernatant were measured using a custom‐made ProcartaPlex multiplex immunoassay (Affymetrix, CA, USA). The immunoassay was performed according to the manufacturer's protocol. Briefly, cytokine standards were resuspended, and serial dilutions were prepared. Antibody magnetic bead mix was added to the plate. After washing, standards and samples were added (50 µL per well), the plate was sealed, and incubated while shaking (30 min at room temperature (RT), overnight at 4 °C, and again 30 min at RT). After washing the plate twice, detection antibodies were added (25 µL per well) and the plate was incubated for 30 min at RT on a plate shaker. After incubation, the plate was washed twice and 50 µL per well streptavidin‐phycoerythrin was added. Again, the plate was incubated at RT for 30 min while shaking. Then, the plate was washed, and 120 µL per well of reading buffer was added. After shaking the plate for 5 min at RT fluorescence was measured using a Luminex 100 System. The data obtained were analyzed using StarStation software.

### 2.7 Fractionation of the Hydrolysate

The hydrolysate was fractionated based on size using an Amicon stirred cell (Merck, Nottingham, UK) with a capacity of 50 mL. In this way, four fractions were obtained, containing peptides and proteins >3 kDa, peptides between 3 and 1 kDa, peptides between 1 and 0.5 kDa, and peptides <0.5 kDa. A detailed description of the procedure can be found in Supporting Information file S2.

The protein concentration in the pooled fractions was measured using a Pierce BCA protein assay, following manufacturer's instructions (Thermoscientific, Pittsburgh, USA). Fractions with a concentration less than 20 mg mL^–1^ were concentrated using a SpeedVac centrifugal evaporator (ThermoScientific, Pittsburgh, USA) for 4 h. The filtered sterile water was concentrated in the same manner. The protein concentration of the concentrated protein fractions were measured again, and were now 20 mg mL^–1^ or higher. Fractions were stored at –20 °C before testing at a concentration of 2 mg mL^–1^ in the HEK‐XBlue‐hTLR2 cell lines as described above.

### 2.8 Peptide Analysis on RP‐Ultra HPLC Coupled to MS

In order to investigate which individual peptides could be responsible for TLR modulating effects, the peptide composition of the specific hydrolysate was fractionated and analyzed with RP‐ultra HPLC (RP‐UHPLC) coupled to MS. Technical details and a description of the procedure can be found in Supporting Information File S2.

The mass tolerance for the accepted annotation was ≤10 ppm between the theoretical mass and the measured mass, which is generally accepted for this type of equipment and method.^37^, ^38^, ^39^ Furthermore, each of the peptide identifications are confirmed by the presence of at least one of their b/y fragments. A generic method was used in which non‐specific enzyme specificity was selected. A list with intact wheat storage proteins used in the analysis is included as Supporting Information File S3. The peptides were annotated by MS and confirmed by MS/MS through the presence of b and y fragment ions with an assigned intensity of at least 50%.

Further analysis was performed by comparing the peptide compositions of the fractions. This was done by investigating which peptides were exclusively present in a fraction with TLR modulating effects. When peptides were present in all fractions, it was determined whether the relative abundance (response) was higher in the specific TLR modulating fraction compared to fractions with lesser effects.

### 2.9 Statistical Analysis

Statistical analysis was performed using Graphpad Prism. Normal distribution of the data was tested using the Kolmogorov–Smirnov test. When data were normally distributed, values were expressed as mean ± standard deviation (SD). Then, a *t*‐test or one‐way ANOVA followed by *t*‐tests was used to identify significant differences. When data were not normally distributed, values were expressed as median with range. Significant differences were in that case assessed using a Kruskal–Wallis test followed by a Dunn's post test. A *p*‐value of <0.05 was considered to indicate a statistical significant difference.

## 3 Results

### 3.1 Characteristics of the Wheat Hydrolysates

Three different wheat hydrolysates with different peptide compositions were investigated (Table 1). Wheat 1 differed most from the other wheat hydrolysates as it was found that Wheat 1 contained more proteins bigger than 10 kDa compared to the other wheat hydrolysates.

### 3.2 Hydrolysate Wheat 1 Strongly Inhibits HKLM Induced TLR2 Activation in a dose Dependent Manner

In order to test the TLR inhibiting capacity of the wheat hydrolysates, HEK‐XBlue‐hTLR2, HEK‐XBlue‐hTLR4, and HEK‐XBlue‐hTLR9 reporter cells were stimulated with 2 mg mL^–1^ hydrolysate and the corresponding ligands to induce TLR activation.

**Figure 1:**
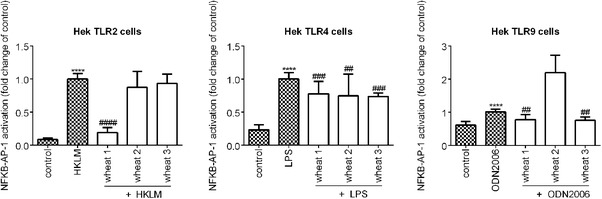
NF‐κB /AP‐1 activation in HEK‐XBlue‐hTLR2, HEK‐XBlue‐hTLR4, and HEK‐XBlue‐hTLR9 cells after simultaneous stimulation with its relevant ligand and 2 mg mL^–1^ of the wheat hydrolysates. Wheat hydrolysates were able to inhibit TLR activation. Hydrolysate Wheat 1 inhibited activation of TLR2, TLR4, and TLR9, with a strong inhibition of TLR2. Wheat 2 inhibited TLR4, while Wheat 3 inhibited TLR4 and TLR9. Data are expressed as median with range (*n* = 5). Significant differences were determined by using the Kruskal–Wallis test followed by the Dunn's test. Significant differences compared to the negative control were indicated by ^*^ *p *< 0.05, ^**^ *p *< 0.01, ^***^ *p *< 0.001), or by ^****^ *p *< 0.0001, significant differences compared to the positive control were indicated by ^#^ *p *< 0.05, ^##^ *p *< 0.01, ^###^ *p *< 0.001, or by ^####^ *p *< 0.0001.

Wheat hydrolysates showed TLR inhibiting capacities after TLR stimulation with a known ligand (**Figure** 1). Wheat 1 had the most pronounced effects. Besides a small, but statistically significant inhibition of TLR4 and TLR9 (both *p *< 0.05), it showed a strong TLR2 inhibiting effect (*p *< 0.05). Wheat 2 only inhibited TLR4 (*p *< 0.05), while Wheat 3 inhibited TLR4 and TLR9 (both *p *< 0.05).

**Figure 2:**
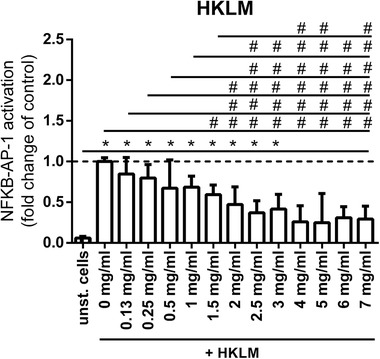
NF‐κB /AP‐1 activation in HEK‐XBlue‐hTLR2 cells after simultaneous stimulation with 10^7^ cells per mL HKLM and graded concentrations of Wheat 1. Wheat 1 showed TLR2 inhibition in a dose dependent way. Data are expressed as median with range (*n* = 5). Significant differences were determined by using the Kruskal–Wallis test followed by the Dunn's test. Significant differences compared to the negative control were indicated by ^*^ *p *< 0.05), ^**^ *p *< 0.01, ^***^ *p *< 0.001, or by ^****^ *p *< 0.0001, significant differences compared to the positive control were indicated by ^#^ *p *< 0.05, ^##^ *p *< 0.01, ^###^ *p *< 0.001, or by ^####^ *p *< 0.0001.

Since the strongest inhibiting effect was induced by Wheat 1 on TLR2, in further experiments, we only used Wheat 1 and studied its effect on TLR2 in more detail. To study the dose response effect of Wheat 1 on TLR2 inhibition, HEK‐XBlue‐hTLR2 cells were incubated with graded concentrations of Wheat 1 and HKLM (10^7^ cells per mL) (**Figure** 2). TLR inhibition was found to be decreased in a dose dependent fashion. A concentration of 1 mg mL^–1^ was the minimal hydrolysate concentration that was still able to induce a significantly decreased TLR2 activation (*p *< 0.05).

### 3.3 Wheat 1 Inhibits TLR2 by Direct Binding to TLR2‐Ectodomain

**Figure 3:**
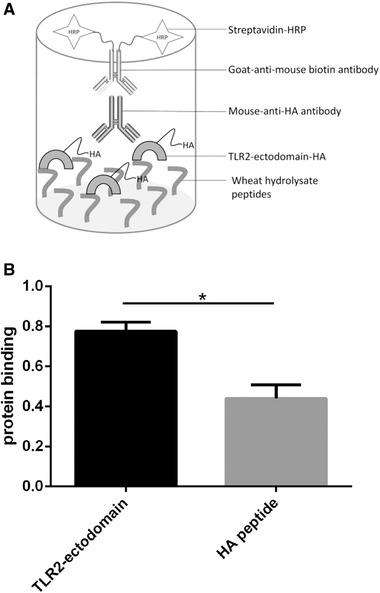
Wheat 1 showed a TLR2‐ectodomain binding effect. ELISA was performed to demonstrate TLR2‐ectodomain binding properties of Wheat 1. ELISA wells were loaded with either 10 µg TLR2‐ectodomain protein. HA peptide was used as a negative control. Data are expressed as mean with SD (*n* = 5). Statistical significant differences compared to the negative control were determined by using a *t*‐test and indicated by ^*^.

In order to determine whether the observed inhibiting effect of Wheat 1 on TLR2 signaling is due to direct binding of the hydrolysate to the TLR2‐ectodomain, an ELISA was developed in which binding of isolated TLR2 protein to immobilized Wheat 1 could be demonstrated. As shown in **Figure** 3, protein binding of TLR2‐ectodomain to Wheat 1 coated wells was significantly higher (*p *< 0.05) compared to the HA negative control when wells were loaded with the TLR2 protein. This indicates that direct binding of wheat peptides to TLR2‐ectodomain occurs.

### 3.4 Wheat 1 was Able to Inhibit Both TLR2/TLR1 and TLR2/TLR6 Signaling

TLR2 is able to form a heterodimer with either TLR1 or TLR6, which leads to different downstream immune responses.^33^, ^34^ In order to determine the heterodimer specificity of the TLR2 inhibiting effect of Wheat 1, HEK‐XBlue‐hTLR2 reporter cells were incubated with different concentrations of Wheat 1 together with either the TLR2/TLR1 ligand P3CSK4^35^ or the TLR2/TLR6 ligand FSL‐1.^36^

**Figure 4:**
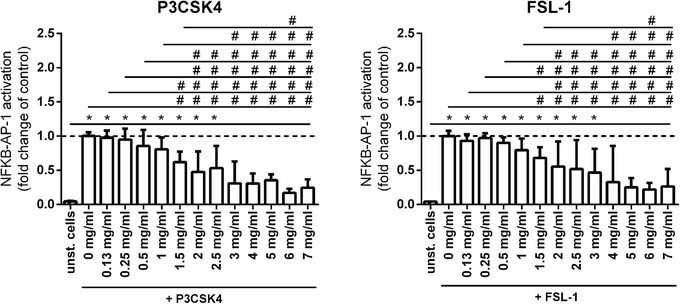
NF‐κB /AP‐1 activation in HEK‐XBlue‐hTLR2 cells after simultaneous stimulation with either the TLR2/1 ligand P3CSK4 or the TLR2/6 ligand FSL‐1 and graded concentrations of Wheat 1. Wheat 1 showed TLR2 inhibition in a dose dependent way, both in 25 ng mL^–1^ P3CSK4 and 25 ng mL^–1^ FSL‐1 stimulated cells. Data are expressed as median with range (*n* = 5). Significant differences were determined by using the Kruskal–Wallis test followed by the Dunn's test. Significant differences compared to the negative control were indicated by ^*^ *p *< 0.05, ^**^ *p *< 0.01, ^***^ *p *< 0.001, or by ^****^ *p *< 0.0001, significant differences compared to the positive control were indicated by ^#^ *p *< 0.05, ^##^ *p *< 0.01, ^###^ *p *< 0.001, or by ^####^ *p *< 0.0001.

As shown in **Figure** 4, Wheat 1 was able to inhibit both P3CSK4 and FSL‐1 induced TLR2 activation. Both for P3CSK4 and FSL‐1 stimulated cells, 1 mg mL^–1^ was the lowest hydrolysate concentration that was still able to induce a statistically significant decreased TLR2 activation compared to ligand stimulated cells (*p *< 0.05).

### 3.5 Wheat 1 Decreased HKLM Induced IL‐6 Production in DCs

**Figure 5:**
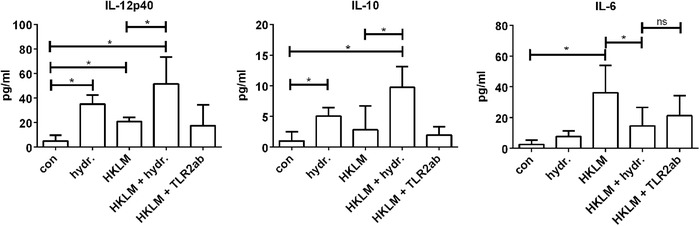
Cytokine production in DCs stimulated with Wheat 1, HKLM, or a combination. Wheat 1 (2 mg mL^–1^) increased IL‐12 and IL‐10 production in DCs, either alone or in combination with HKLM. Wheat 1 was able to inhibit HKLM induced IL‐6 production. Preincubation of DCs with a TLR2 antibody showed the same effect. Data are expressed as mean with SD (*n* = 5). Statistical significant differences compared to the negative control were determined by using *t*‐tests and indicated by ^*^.

Next, we investigated the effects of Wheat 1 on HKLM stimulated human DCs. DCs are important in orchestrating intestinal immune responses.^40^, ^41^ **Figure** 5 shows the effects of 2 mg mL^–1^ Wheat 1 on the production of the regulatory cytokine IL‐10, the proinflammatory cytokine IL‐12 and the pleiotropic cytokine IL‐6 of HKLM stimulated DCs, which are associated with DC activation.^42^ Effects on other cytokines measured are shown in Supporting Information File S4.

The production of IL‐12 was significantly increased after DC stimulation for 24 h with either Wheat 1 alone or HKLM alone compared to the negative control (both *p *< 0.05). When DCs were treated with a combination of TLR2 activating HKLM and Wheat 1, IL‐12 production was also significantly increased compared to unstimulated cells (*p *< 0.05), and this effect was even significantly higher compared to DCs treated with HKLM alone. The TLR2 blocking antibody had no effect on the IL‐12 production of HKLM stimulated cells.

For IL‐10, a similar effect was observed. Stimulation of DCs with Wheat 1 alone increased IL‐10 production significantly (*p *< 0.05), while HKLM stimulation had no significant effect on IL‐10 production by DCs. DCs treated with a combination of Wheat 1 and HKLM did result in an increased IL‐10 production, which was significantly different from both the unstimulated cells and HKLM treated cells (both *p *< 0.05). Again, the TLR2 blocking antibody did not affect HKLM stimulated DC IL‐10 production.

For IL‐6 another effect was observed. Stimulation with Wheat 1 alone did not change IL‐6 production of DCs, while HKLM stimulation significantly increased IL‐6 production compared to the IL‐6 production of untreated cells (*p *< 0.05). Interestingly DCs were treated with a combination of HKLM and Wheat 1, IL‐6 production was significantly decreased compared to DCs stimulated with HKLM alone (*p *< 0.05). This effect could be induced via TLR2, since the IL‐6 production in DCs treated with HKLM after preincubation with the TLR2 blocking antibody was similar to DCs treated with HKLM and Wheat 1.

### 3.6 The Peptide Fraction Containing Peptides Smaller than 0.5 kDa has the Strongest TLR2 Inhibiting Effects

To investigate which peptide(s) in Wheat 1 are responsible for the TLR inhibiting effects, size based fractions were produced (>3 kDa, 3–1 kDa, 1–0.5 kDa, and <0.5 kDa), and tested at a concentration of 2 mg mL^–1^ for TLR2 inhibiting effects in HKLM stimulated HEK‐XBlue‐hTLR2 reporter cells.

**Figure 6:**
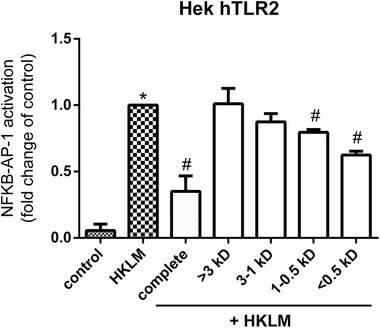
NF‐κB /AP‐1 activation in HEK‐XBlue‐hTLR2 cells after simultaneous stimulation with 10^7^ cells per mL HKLM and different size based fractions of Wheat 1. Only the two smallest fractions, 1–0.5 kDa and <0.5 kDa, showed TLR2 inhibition in HKLM stimulated cells. Data were expressed as median with range (*n* = 5). Significant differences were determined by using the Kruskal–Wallis test followed by the Dunn's test. Significant differences compared to the negative control were indicated by ^*^ *p *< 0.05, ^**^ *p *< 0.01), ^***^ *p *< 0.001, or by ^****^ *p *< 0.0001, significant differences compared to the positive control were indicated by ^#^ *p *< 0.05, ^##^ *p *< 0.01, ^###^ *p *< 0.001, or by ^####^ *p *< 0.0001.

As shown in **Figure** 6, it was found that only the fractions 1–0.5 kDa and <0.5 kDa showed a significantly reduced TLR2 activation compared to cells treated with HKLM alone (both *p *< 0.05). The fraction containing the peptides smaller than 0.5 kDa showed the strongest TLR2 inhibiting effect.

### 3.7 Peptides with a Potential TLR2 Inhibiting Effect were Identified in the Fraction <0.5 kDa

To identify the specific peptides responsible for the TLR2 inhibition in the fractions 1–0.5 kDa and <0.5 kDa, RP‐UHPLC coupled to MS was applied. The analysis also included the fraction 3–1 kDa, since this fraction contained no inhibiting effect. In the fraction 3–1 kDa 1300 peptides confirmed by minimal 1 first generation primary ion in the MS/MS signal were identified. In the fraction 1–0.5 kDa, 930 peptides were identified, and in the fraction <0.5 kDa, 861 peptides were identified (see Supporting Information File S5 for complete lists).

Differences in peptide composition can already be observed between fractions, by comparing UV absorbance profiles at 214 nm and mass spectrum (Supporting Information File S6 and S7).

To identify which peptides were uniquely present in TLR2 inhibiting fractions (<0.5 kDa and 1–0.5 kDa), the different peptides in the three fractions were compared, and only those with an assigned intensity of 50% or higher were selected. The peptide annotation was confirmed for all peptides by identification of b and y fragments by MS/MS. The peptides IFWGIPA and IAPVGIF were present in all fractions, and did not differ in relative abundance (response). The peptides MHILLP, TTIAPFGIFGTN, ILQQQL, and VCSVSQIIMRQ were unique for the <0.5 kDa fraction and absent in both the 1–0.5 kDa and 3–1 kDa fractions. Interestingly, the peptide WQIPEQSR (four matched first gen primary ions found) was present in both TLR2 inhibiting fractions, but not in the 3–1 kDa fraction without TLR2 inhibiting effects (**Table** 3). The relative abundance (response) of the peptide WQIPEQSR was similar in both fractions.

**Table 3:** List of possible TLR2‐inhibiting peptides

Peptide	Observed mass (Dalton) [M + H]	Source protein	Present in fraction		
			3–1 kDa	1–0.5 kDa	<0.5 kDa
MHILLP	723	ɣ‐gliadin	No	No	Yes
TTIAPFGIFGTN	1238	α/β gliadin	No	No	Yes
ILQQQL	742	α/β ‐gliadin, α‐gliadin	No	No	Yes
VCSVSQIIMRQ	1263	Avidin like A2,A3, A7	No	No	Yes
WQIPEQSR	1043	α/β gliadin	No	Yes	Yes

## 4 Discussion

Wheat hydrolysates are used for medical nutrition to provide undernourished patients a readily digestible protein source, for example, to recover from chemotherapy induced damage to the intestine. Another potential beneficial effect of hydrolysates is modulation of the immune system to inhibit chemotherapy‐induced intestinal damage and inflammation. There is evidence that Toll‐like receptors (TLRs) are involved in the induction of mucositis^21^ and some hydrolysates have been found to be capable of TLR modulation.^15^ Therefore, we studied TLR inhibiting effects of three wheat hydrolysates used in medical nutrition.

The inhibiting capacities of the three wheat hydrolysates were first studied for TLR2, TLR4, and TLR9, which are all associated with mucositis development.^21^, ^22^ They all had TLR inhibitory effects but to a varying extent and the effect was highly hydrolysate dependent. Wheat 1 showed a strong TLR2 inhibiting effect, which was not observed for the other hydrolysates. As shown in Figure 3, the inhibition of TLR2 activation by Wheat 1 was most likely due to direct binding of Wheat 1 to the TLR2‐ectodomain, and therewith blocking of TLR2 activation by potential proinflammatory DAMPs or other ligands. TLR2 has been shown before to be able to bind to proteins and (synthetic) peptides.^43^, ^44^, ^45^ The TLR2 ligand binding site has a large binding surface with many insertions and β‐sheets to which many proteins can bind.^46^ TLR2 is the most studied TLR in relation with mucositis.^21^, ^47^, ^48^ Its blockade can prevent doxorubicin‐induced mucositis.^21^ Doxorubicin is one of the most potent and commonly applied chemotherapeutic drugs,^49^ but its application is sometimes limited by its toxicity.^50^ Our data suggest that wheat hydrolysates might be instrumental in doxorubicin treated patients by increasing its therapeutic potential due to its TLR2 inhibiting effects. Since TLR2 regulation of the intestinal damage was found to be chemotherapeutic drugs specific,^23^ the effects of wheat hydrolysates might differ between chemotherapies.

TLR2 has the unique capacity to form dimers with TLR1 or TLR6.^32^ Depending on the dimer formed, more pro‐ or anti‐inflammatory responses are induced.^33^, ^34^ To better understand the effects of TLR2 inhibition by Wheat 1, we investigated whether the inhibiting effect was specific for TLR2/1 or TLR2/6 or a combination thereof. It was found that Wheat 1 inhibited both TLR2/1 as well as TLR2/6 signaling (Figure 4), indicating that Wheat 1 may block the TLR2 ligand binding site itself, in such a way that it does not interfere with the TLR2/1 and TLR2/6 dimerization.

Stimulating DCs with Wheat 1, HKLM or a combination had distinct effects on the production of the cytokines IL‐12, IL‐10, and IL‐6 (Figure 5). TLR2 activation by HKLM had a pronounced effect on IL‐6 production, which is known to be merely TLR2 dependent.^51^, ^52^ Although IL‐6 is a pleiotropic cytokine, it is known to enhance pro‐inflammatory events during intestinal injury. In mucositis, IL‐6, together with TNF‐α and IL‐1β, was found to be significantly increased both in blood and intestinal tissue in animal models and patients.^53^, ^54^, ^55^ Especially IL‐6 levels correlate with the severity of the inflammation,^56^ and reduction of IL‐6 attenuates intestinal inflammation.^57^ Here, we found that Wheat 1 was able to inhibit the HKLM induced IL‐6 production in DCs. Therefore, administration of wheat hydrolysate might be a new way to provide nutrients and simultaneously inhibit IL‐6, although more research is needed to confirm this. IL‐10 and IL‐12, nor any of the other cytokines measured, were inhibited by Wheat 1, which confirms the TLR2 specificity of the inhibiting effect of Wheat 1, as regulation of these cytokines depends more on other TLR types such as TLR4.^58^, ^59^

It is likely that only specific protein sequences in the wheat hydrolysate are responsible for the TLR2 binding effect. Therefore, we designed a strategy to identify the unique peptide sequences in Wheat 1 that might be responsible for the TLR2 inhibiting effects. We obtained size based fractions of the hydrolysate and detected most of the TLR2 inhibitory effects in the smallest fraction (<0.5 kDa). Four possible TLR2 inhibiting peptides in this fraction were identified by analyzing the fractions using RP‐UHPLC coupled to MS and comparing their peptide composition. Since only the peptide WQIPEQSR was present in both fractions showing TLR inhibition and since it was absent in the fraction without TLR2 effects, we propose that this peptide is most likely essential for TLR2 inhibition. However, since none of the tested fractions was able to recapitulate the TLR2 inhibiting effect of the complete hydrolysate, further studies should be performed to investigate possible synergic effects with other peptides. Modulation of TLR signaling by small molecules, which do not resemble PAMPs, is of recent interest.^45^, ^60^ Molecules have for example been found to bind the ligand site,^61^ but also intracellular regions,^62^ or interfere with dimerization and the downstream pathway.^63^ It might be argued that the activity of this peptide might disappear during gastrointestinal transition due to proteolytic digestion but it is known that WQIPEQSR will stay intact for a significant amount of time in the intestine, and will therefore be able to induce immune effect in the intestine. WQIPEQSR is a gliadin derived peptide and very stable even after treatment with pancreatic proteases, as well as after treatment with brush border proteolytic enzymes.^64^, ^65^

In this study, we identified a wheat hydrolysate with a strong TLR2 inhibitory effect due to binding of the TLR2‐ectodomain, which resulted in HKLM induced IL‐6 inhibition in DCs. Since TLR2 and IL‐6 are recognized to be crucial in the development of intestinal mucositis, future research should focus on testing whether wheat hydrolysates can be applied in clinical nutrition to attenuate mucositis. Overall, our findings provide a good start for further research to investigate whether this hydrolysate might contribute to the management of mucositis in cancer patients receiving chemotherapy.

## Conflict of Interest

The authors declare no conflict of interest.

## Supporting information

Supplementary

Supplementary

Supplementary

Supplementary

Supplementary

Supplementary

Supplementary
