# Supplementary material for: Identification of a TLR2 Inhibiting Wheat Hydrolysate
Source: Mol Nutr Food Res. 2018 Nov 2;62(23):1800716. doi: 10.1002/mnfr.201800716 (PMC6646915; doi:10.1002/mnfr.201800716)
Supplement: Supplementary file 1 — Supplementary [file MNFR-62-na-s001.pdf]

Supplementary file 1:

A

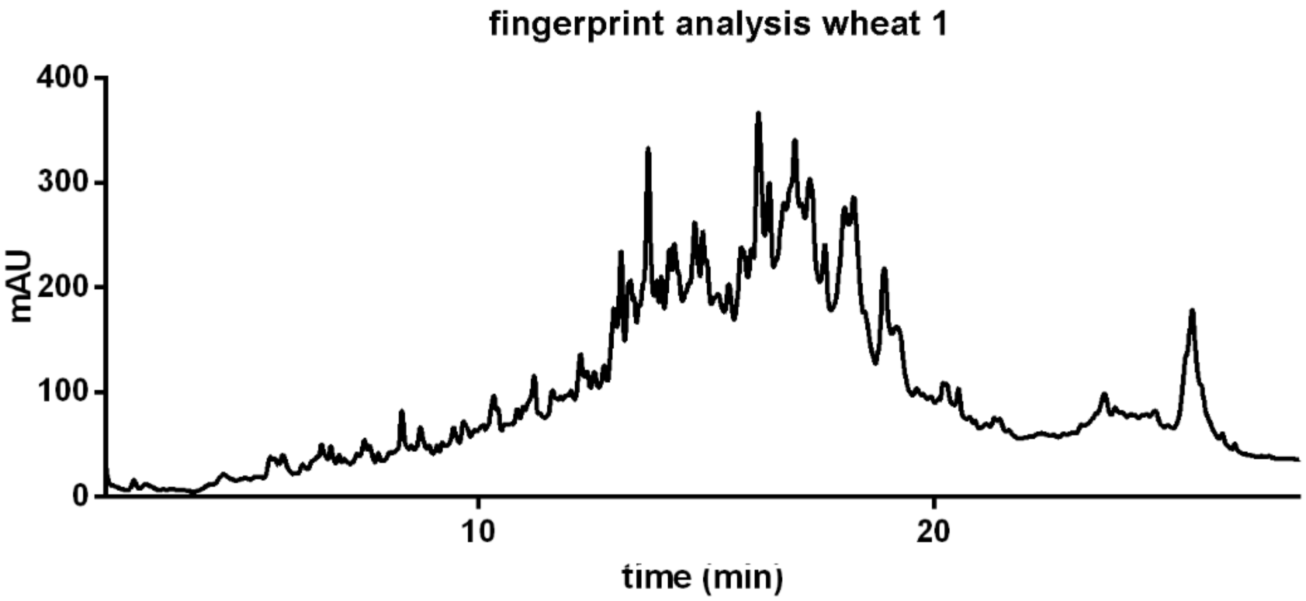

B

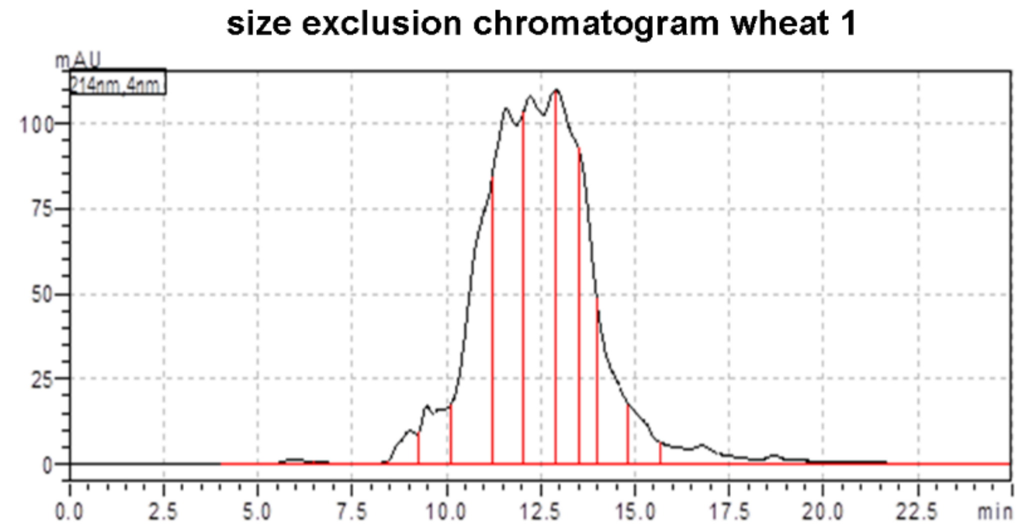

Supplementary file 2:

Reporter cell assay

To test whether the wheat hydrolysates are able to inhibit TLR2, 4 and 9 activation induced by known ligands, the samples were tested on a HEK-XBlue^{TM}-hTLR2, 4 and 9 (Invivogen, Toulouse, France) reporter cell assay.

For the assay, cells were seeded in a flat bottom 96 wells plate at a concentration described in table 1 (180 µL/well). Cells were stimulated with 2 mg/mL wheat hydrolysate, and the respective activating ligand (table 1) at the same time, and incubated for 24 hours (37℃, 95% oxygen, 5% CO_2). TLR ligand alone was used as a positive control. Medium was used as a negative control. After incubation, Quanti-Blue detection medium was added as described before [27]. Absorbance (650 nm) was quantified using a VersaMax microplate reader (Molecular Devices GmbH, Biberach an der Riss, Germany) and SoftMax Pro Data Acquisition & Analysis Software to determine SEAP activity, which represents activation of NF-κB/AP-1. The median and range for each sample were plotted as the fold-change compared to the positive control, which were TLR ligand stimulated cells. The positive controls were set at 1.

**Cell line**	**Cell density for seeding**	**Positive control (concentration in well)**
HEK-Blue human TLR2	2.8*10^5 cells/mL (180 µl/well)	Heat killed Listeria monocytogenes (10^7 cells/mL)<br>P3CSK4 (25 ng/mL)<br>FSL-1 (25 ng/mL)
HEK-Blue human TLR4	1.4*10^5 cells/mL (180 µl/well)	*Escherichia coli K12* Lipopolysaccharide (10 ng/mL)
HEK-Blue human TLR9	4.5*10^5 cells/mL (180 µl/well)	Type B CpG oligonucleotide (ODN 2006, 0,25 µM)

Cell densities and ligands used in the different reporter cell line assays.

Fractionation

The hydrolysate was fractionated based on size using an Amicon stirred cell (Merck, Nottingham, UK) with a capacity of 50 mL. Filter membranes with MWCO's of 3 kD (regenerated cellulose), 1 kD (regenerated cellulose) and 0.5 kD (cellulose acetate) (all Merck, Nottingham, UK) were used to prepare fractions containing peptides and proteins >3 kD, peptides between 3 and 1 kD, peptides between 1 and 0.5 kD and peptides <0.5 kD.Before use, the ultracentrifugal unit was sterilized, and the collecting tubes were cleaned with 70% ethanol. Filter membranes with MWCO's of 3 kD (regenerated cellulose), 1 kD (regenerated cellulose) and 0.5 kD (cellulose acetate) (all Merck, Nottingham, UK) were used to prepare fractions containing peptides and proteins >3 kD, peptides between 3 and 1 kD, peptides between 1 and 0.5 kD and peptides <0.5 kD. To remove glycerine from the membranes before use, the membranes were soaked in sterile H_2O for 1 hour.

Then, the hydrolysate was dissolved in 50 mL sterile water at a concentration of 40 mg/mL. To remove undissolved particles that could block the filtration membranes, the hydrolysate was centrifuged at 4000xg for 5 min, after which the supernatant was used for

further processing. Two mL of the hydrolysate was stored at -20℃. The rest was first filtered using the 3 kD filter under continuous stirring, by applying a N_2 pressure (3,5 bar). The permeate was collected in a 50 mL tube, until approximately 80% of the sample was filtered. Then, the filtration was stopped, and the retentate was stirred for 15 min to remove proteins from the membrane. The retentate was also collected in a 50 mL tube. The described filtration steps were repeated for the collected 3 kD permeate using the 1 kD filter and for the collected 1 kD permeate using the 0.5 kD filter. Sterile water was filtered in the same manner to check for contamination during the filtration steps. The filtration process was performed 7 times and fractions were pooled in order to collect enough volume of the sample for subsequent experiments.

RP-UHPLC

In order to investigate which individual peptides could be responsible for TLR modulating effects, the peptide composition of the specific hydrolysate was fractionated and analyzed with RP-UHPLC coupled to MS.

To this end, wheat fraction samples (3-1 kD, 1-0.5 D and <0.5 kD) were diluted 4x with Mili-Q water. The diluted samples were centrifuged (16.110 g for 10 min), after which the supernatant was transferred to an HPLC vial. The obtained fractions were analyzed on an H class Acquity UPLC system (Waters, Milford, MA, USA) equipped with a BEH C18 column (1.7 µm, 2.1×100 mm, Waters) with an Acquity BEH C18 guard precolumn. The UPLC system was coupled to an Acquity 145 UPLC® PDA detector (Waters). Separation was carried out using the following elution profile at a flowrate of 0.350 mL/min: 5% ACN isocratic for 2 minutes; 5-42% ACN in 37 minutes; isocratic cleaning step of 90% ACN for the duration of 3 minutes; and re-equilibration to starting conditions for 6 minutes. Ultraviolet (UV) data was acquired using MassLynx software (Waters).

The mass spectra of the peptides were determined with Electron Spray Ionization Time of Flight Mass Spectrometry (ESI -Q-TOF-MS), using an online SYNAPT G2-Si high definition mass spectrometer (Waters) coupled to the RP-UHPLC. The system was calibrated with sodium iodide. The capillary voltage was set to 3 kV with the source operation in positive ion mode and the source temperature at 150℃. The sample cone was operated at 40 V. Nitrogen was used as desolvation gas (500℃, 800 L/h) and cone gas (200 L/h). MS and MS/MS (Resolution method) data were collected between m/z 100-3000 with a scan time of 0.3 seconds. Online lock mass data (Angiotensin II, Mw 523.7751 Da) were collected and the correction was applied during data reprocessing. The data were analyzed using Unifi software (Waters).

Supplementary file 3:

List of reference proteins

Search strategy Uniprot: ‘triticum aestivum’+‘storage protein’ → 72 hits
Excluded enzymes and unknown fragments

Glutenin, high molecular weight subunit DY10 EGEASRQLQCERELQESSLEACRQVVDQQLAGRLPWSTG
LQMRCCQQLRDVSAKCRSVAVSQVARQYEQTVVPPKGGSFYPGETTPLQQLQQGIFWGTS
SQTVQGYYPGVTSPRQGSYYPGQASPQQPGQGQQPGKWQEPGQGQQWYYPTSLQQPGQGQ
QIGKGQQGYYPTSLQQPGQGQQGYYPTSLQHTGQRQQPVQGQQPEQGQQPGQWQQGYYPT
SPQQLGQGQQPRQWQQSGQGQQGHYPTSLQQPGQGQQGHYLASQQQPGQGQQGHYPASQQQPGQG
QQGHYPASQQQPGQGQQGHYPASQQEPGQGQQGQIPASQQQPGQGQQGHYPASLQQPGQGQQGHYP
TSLQQLGQGQQTGQPGQKQQPGQGQQTGQGQQPEQEQQPGQGQQGYYPTSLQQPGQGQQQGQGQQG
YYPTSLQQPGQGQQGHYPASLQQPGQGQPGQRQQPGQGQHPEQGKQPGQGQQGYYPTSPQQPGQGQ
QLGQGQQGYYPTSPQQPGQGQQPGQGQQGHCPTSPQQSGQAQQPGQGQQIGQVQQPGQGQQGYYPTS
VQQPGQGQQSGQGQQSGQGHQPGQGQQSGQEQQGYDSPYHVSAEQQAASPMVAKAQQPATQLPTVC
RMEGGDALSASQ

Glutenin, high molecular weight subunit DX5 EGEASEQLQCERELQELQERELKACQQVMDQQLRDISPE
CHPVVVSPVAGQYEQQIVVPPKGGSFYPGETTPPQQLQQRIFWGIPALLKRYYPSVTCPQ
QVSYYPGQASPQRPGQGQQPGQGQQGYYPTSPQQPGQWQQPEQGQPRYYPTSPQQSGQLQ
QPAQGQQPGQGQQGQQPGQGQPGYYPTSSQLQPGQLQQPAQGQQGQQPGQAQQGQQPGQGQQPGQG
QQGQQPGQGQQPGQGQQGQQLGQGQQGYYPTSLQQSGQGQPGYYPTSLQQLGQGQSGYYPTSPQQP
GQGQQPGQLQQPAQGQQPGQGQQGQQPGQGQQGQQPGQGQQPGQGQPGYYPTSPQQSGQGQPGYYP
TSSQQPTQSQQPGQGQQGQQVGQGQQAQQPGQGQQPGQGQPGYYPTSPQQSGQGQPGYYLTSPQQSG
QGQQPGQLQQSAQGQKGQQPGQGQQPGQGQQGQQPGQGQQGQQPGQGQPGYYPTSPQQSGQGQQP
GQWQQPGQGQPGYYPTSPLQPGQGQPGYDPTSPQQPGQGQQPGQLQQPAQGQQGQQLAQGQQGQQP
AQVQQGQRPAQGQQGQQPGQGQQGQQLGQGQQGQQPGQGQQGQQPAQGQQGQQPGQGQQGQQPG
QGQQGQQPGQGQQPGQGQPWYYPTSPQESGQGQQPGQWQQPGQGQPGYYLTSPLQLGQGQQGYYPT
SLQQPGQGQQPGQWQQSGQGQHWYYPTSPQLSGQGQRPGQWLQPGQGQQGYYPTSPQQPGQGQQLG
QWLQPGQGQQGYYPTSLQQTGQGQQSGQGQQGYYSSYHVSVEHQAASLKVAKAQQLAAQLPAMCR
LEGGDALSASQ

Avenin-like b1
QLETTCSQGFGQYQQQQQPGQRQLLEQMKPCVAFLQQQCRPL
RMPFLQTQVEQLSSCQIVQHQCCQQLAQIPERIRCHAIHSVVEAIMQQQSQQQWQERQQQ
AQHKSMRMLLENLSLMCNIYVPVQCQQQQQMGQQQQQQQLQEQLTPCATFLQHQCSPVTV
PFPQIPVDQPTSCQNVQHQCCRQLSQIPEQFRCQAIHNVAEAIRQQQPQQQWQGMYQPQQ
PAQHESIRMSLQALRSMCNIYIPVQCPAPTAYNIPMVATCTSGAC

Alpha/beta-gliadin
VRVPVPQLQPQNPSQQQPQEQVPLVQQQQFPGQQQPFPPQ
QPYPQPQPFPSQQPYLQLQPFPQPQLPYPQPQLPYPQPQLPYPQPQPFRPQQPYPQSQPQ
YSQPQQPISQQQQQQQQQQQQKQQQQQQQQILQQILQQQLIPCRDVVLQQHSIAYGSSQV
LQQSTYQLVQQLCCQQLWQIPEQSRCQAIHNVVHAIILHQQQQQQQQQQQQPLSQVSFQQ
PQQQYPSGQGSFQPSQQNPQAQGSVQPQQLPQFEEIRNLALETLPAMCNVYIPPYCTIAP
VGIFGTN
Glutenin, low molecular weight subunit 1D1 RCIPGLERPWQQQPLPPQQTFPQQPLFSQQQQQQLFP
QQPSFSQQQPPFWQQQPPFSQQQPILPQQPPFSQQQQLVLPQQPPFSQQQQPVLPPQQSP
FPQQQQQHQQLVQQQIPVVQPSILQQLNPCKVFLQQQCSPVAMPQRLARSQMLQQSSCHV
MQQQCCQQLPQIPQQSRYEAIRAIIYSIILQEQQQVQGSIQSQQQQPQQLGQCVSQPQQQ
SQQQLGQQPQQQQLAQGTFLQPHQIAQLEVMTSIALRILPTMCSVNVPLYRTTTSVPFGV
GTGVGAY

Glutenin, high molecular weight subunit 12
EGEASRQLQCERELQESSLEACRQVVDQQLAGRLPWSTG
LQMRCCQQLRDVSAKCRSVAVSQVARQYEQTVVPPKGGSFYPGETTPLQQLQQGIFWGTS
SQTVQGYYPSVTSPRQGSYYPGQASPQQPGQGQQPGKWQEPGQGQQWYYPTSLQQPGQGQ

QIGKGKQGYYPTSLQQPGQGQQIGQGQQGYYPTSPQHTGQRQQPVQGQQIGQGQQPEQGQ
QPGQWQQGYYPTSPQQLGQGQQPGQWQQSGQGQQGHYPTSLQQPGQGQQGHYLASQQQPAQGQQG
HYPASQQQPGQGQQGHYPASQQQPGQGQQGHYPASQQEPGQGQQGQIPASQQQPGQGQQGHYPASL
QQPGQQGHYPTSLQQLGQGQQIGQPGQKQQPGQGQQTGQGQQPEQEQQPGQGQQGYYPTSLQQPGQ
GQQQGQGQQGYYPTSLQQPGQGQQGHYPASLQQPGQGQGQPGQRQQPGQGQHPEQGQQPGQGQQG
YYPTSPQQPGQGQQLGQGQQGYYPTSPQQPGQGQQPGQGQQGHCPMSPQQTGQAQQLGQGQQIGQV
QQPGQGQQGYYPTSLQQPGQGQQSGQGQQSGQGHQPGQGQQSGQEKQGYDSPYHVSAEQQAASPMV
AKAQQPATQLPTVCRMEGGDALSASQ

Glutenin, low molecular weight subunit PTDUCD1
METSCIPGLERPWQEQPLPPQHTLFPQQQPFPQQQQPPFS
QQQPSFLQQQPILPQLPFSQQQQPVLPQQSPFSQQQLVLPPQQQYQQVLQQQIPIVQPSV
LQQLNPCKVFLQQQCNPVAMPQRLARSQMLQQSSCHVMQQQCCQQLPQIPEQSRYDVIRA
ITYSIILQEQQQGFVQAQQQQPQQLGQGVSQSQQQSQQQLGQCSFQQPQQQLGQQPQQQQ
VLQGTFLQPHQIAHLEVMTSIALRTLPTMCSVNVPLYSSTTSVPFSVGTGVGAYL

Glutenin, high molecular weight subunit PW212 EGEASEQLQCERELQELQERELKACQQVMDQQLRDISPE
CHPVVVSPVAGQYEQQIVVPKGGSFYPGETTPPQQLQQRIFWGIPALLKRYYPSVTSPQQ
VSYYPGQASPQRPGQGQQPGQGQQSGQGQQGYYPTSPQQPGQWQQPEQGQPGYYPTSPQQ
PGQLQQPAQGQQPGQGQQGRQPGQGQPGYYPTSSQLQPGQLQQPAQGQQGQQPGQGQQGQQPGQGQ
QPGQGQQGQQPGQGQQPGQGQQGQQLGQGQQGYYPTSLQQSGQGQPGYYPTSLQQLGQGQSGYYPT
SPQQPGQGQQPGQLQQPAQGQQPEQGQQGQQPGQGQQGQQPGQGQQPGQGQPGYYPTSPQQSGQGQ
PGYYPTSSQQPTQSQQPGQGQQGQQVGQGQQAQQPGQGQQPGQGQPGYYPTSPLQSGQGQPGYYLTS
PQQSGQGQQPGQLQQSAQGQKGQQPGQGQQPGQGQQGQQPGQGQQGQQPGQGQPGYYPTSPQQSGQ
GQQPGQWQQPGQGQPGYYPTSPLQPGQGQPGYDPTSPQQPGQGQQPGQLQQPAQGQQGQQLAQGQQ
GQQPAQVQQGQQPAQGQQGQQLGQGQQGQQPGQGQQPAQGQQGQQPGQGQQGQQPGQGQQPGQG
QPWYYPTSPQESGQGQQPGQWQQPGQWQQPGQGQPGYYLTSPLQLGQGQQGYYPTSLQQPGQGQQP
GQWQQSGQGQHGYYPTSPQLSGQGQRPGQWLQPGQGQQGYYPTSPQQSGQGQQLGQWLQPGQGQQ
GYYPTSLQQTGQGQQSGQGQQGYYSSYHVSVEHQAASLKVAKAQQLAAQLPAMCRLEGGDALSASQ
Glutenin, low molecular weight subunit
QISQQQQAPPFSQQQQPPFSQQQQPPFSQQQQSPFSQQQQQ
PPFAQQQQPPFSQQPPISQQQQPPFSQQQQPQFSQQQQPPYSQQQQPPYSQQQQPPFSQQ
QQPPFSQQQQQPPFTQQQQQQQQQQPFTQQQQPPFSQQPPISQQQQPPFLQQQRPPFSRQ
QQIPVIHPSVLQQLNPCKVFLQQQCIPVAMQRCLARSQMLQQSICHVMQQQCCQQLRQIP
EQSRHESIRAIIYSIILQQQQQQQQQQQQQQGQSIIQYQQQQPQQLGQCVSQPLQQLQQQ
LGQQPQQQQLAHQIAQLEVMTSIALRTLPTMCNVNVPLYETTTSVPLGVGIGVGVY

Avenin-like a1
QLYTTCSQGYGQCQQQPQPQPQPQPQMNTCAAFLQQCSQTP
HVQTQMWQASGCQLVRQQCCQPLAQISEQARCQAVCSVAQIIMRQQQGQSFGQPQQQVPV
EIMRMVLQTLPLMCRVNIPQYCTTTPCSTITPAIYSIPMTATCAGGAC

Alpha/beta-gliadin
VRFPVPQLQPQNPSQQQPQEQVPLVQQQQFLGQQQPFPPQ
QPYPQPQPFPSQLPYLQLQPFPQPQLPYSQPQPFRPQQPYPQPQPQYSQPQQPISQQQQQ
QQQQQQQQQQQQQILQQILQQQLIPCMDVVLQQHNIAHGRSQVLQQSTYQLLQELCCQHL
WQIPEQSQCQAIHNVVHAIILHQQQKQQQQPSSQVSFQQPLQQYPLGQGSFRPSQQNPQA
QGSVQPQQLPQFEEIRNLALQTLPAMCNVYIPPYCTIAPFGIFGTN

Avenin-like a5
QLDTTCSQGYGQCQQQPQQQVNTCSALLQQCSPTPYVQSQM
WQASGCQLMRQQCCQPLAQISEQARCHAVCGVAQVIMRQQQGQSFGQPQQQQGQSFSQPQ
QQVPIEIRRMVLQTLPSMCNVNIPQYCTTTPCSTITQTPYNVPMATTCVGGTC

Alpha/beta-gliadin A-II
VRVPVPQLQLQNPSQQQPQEQVPLVQEQQFQGQQQPFPPQ
QPYPQPQPFPSQQPYLQLQPFPQPQLPYPQPQPFRPQQPYPQPQPQYSQPQQPISQQQQQ
QQQQQQQQQQILQQILQQQLIPCRDVVLQQHNIAHGSSQVLQESTYQLVQQLCCQQLWQI
PEQSRCQAIHNVVHAIILHQQHHHHQQQQQQQQQQPLSQVSFQQPQQQYPSGQGFFQPSQ
QNPQAQGSFQPQQLPQFEEIRNLALQTLPAMCNVYIPPYCTIAPFGIFGTN

Alpha/beta-gliadin A-III
VRVPVPQLQPQNPSQQQPQEQVPLMQQQQQFPGQQEQFPP
QQPYPHQQPFPSQQPYPQPQPFPPQLPYPQTQPFPPQQPYPQPQPQYPQPQQPISQQQAQ
QQQQQQQTLQQILQQQLIPCRDVVLQQHNIAHASSQVLQQSSYQQLQQLCCQQLFQIPEQ
SRCQAIHNVVHAIILHHHQQQQQQPSSQVSYQQPQEQYPSGQVSFQSSQQNPQAQGSVQP
QQLPQFQEIRNLALQTLPAMCNVYIPPYCSTTIAPFGIFGTN

Alpha/beta-gliadin A-IV
VRVPVPQLQPQNPSQQQPQKQVPLVQQQQFPGQQQPFPPQ
QPYPQQQPFPSQQPYMQLQPFPQPQLPYPQPQLPYPQPQPFRPQQSYPQPQPQYSQPQQP
ISQQQQQQQQQQQQQQQQILQQILQQQLIPCRDVVLQQHSIAHGSSQVLQQSTYQLVQQFC
CQQLWQIPEQSRCQAIHNVVHAIILHQQQQQQQQQQQQQQPLSQVCFQQSQQQYPSGQG
SFQPSQQNPQAQGSVQPQQLPQFEEIRNLALETLPAMCNVYIPPYCTIAPVGIFGTN
Alpha/beta-gliadin clone PW1215
VPVPQPQPQNPSQPQPQGQVPLVQQQQFPGQQQQFPPQ
QPYPQPQPFPSQQPYLQLQPFPQPQPFPPQLPYPQPPPFSPQQPYPQPQPQYPQPQQPIS
QQQAQQQQQQQQQQQQQQQQQQILQQILQQQLIPCRDVVLQQHNIAHARSQVLQQSTYQP
LQQLCCQQLWQIPEQSRCQAIHNVVHAIILHQQQRQQQPSSQVSLQQPQQQYPSGQGFFQ
PSQQNPQAQGSVQPQQLPQFEEIRNLALQTLPRMCNVYIPPYCSTTIAPFGIFGTN

Alpha/beta-gliadin A-V
VRVPVPQLQPQNPSQQQPQEQVPLVQQQQFPGQQQQFPPQ
QPYPQPQPFPSQQPYLQLQPFPQPQPFPPQLPYPQPQSFPPQQPYPQQQPQYLQPQQPIS
QQQAQQQQQQQQQQQQQQQILQQILQQQLIPCRDVVLQQHNIAHASSQVLQQSTYQLLQQ
LCCQQLLQIPEQSQCQAIHNVAHAIIMHQQQQQQQEQKQQLQQQQQQQQLQQQQQQQQQPSSQVS
FQQPQQQYPSSQVSFQPSQLNPQAQGSVQPQQLPQFAEIRNLALQTLPAMCNV
YIPPHCSTTIAPFGISGTN

Alpha/beta-gliadin A-I
VRVPVPQLQPQNPSQQQPQEQVPLVQQQQFLGQQQPFPPQ
QPYPQPQPFPSQQPYLQLQPFLQPQLPYSQPQPFRPQQPYPQPQPQYSQPQQPISQQQQQ
QQQQQQQQQQQQQIIQQILQQQLIPCMDVVLQQHNIVHGKSQVLQQSTYQLLQELCCQH
LWQIPEQSQCQAIHNVVHAIILHQQQKQQQQPSSQVSFQQPLQQYPLGQGSFRPSQQNPQ
AQGSVQPQQLPQFEEIRNLARK

Alpha/beta-gliadin clone PW8142
PVPQLQPKNPSQQQPQEQVPLVQQQQFPGQQQQFPPQQPY
PQPQPFPSQQPYLQLQPFPQPQPFLPQLPYPQPQSFPPQQPYPQQRPKYLQPQQPISQQQ
AQQQQQQQQQQQQQQQQQILQQILQQQLIPCRDVVLQQHNIAHASSQVLQQSTYQLLQQL
CCQQLLQIPEQSRCQAIHNVVHAIIMHQQEQQQQLQQQQQQQLQQQQQQQQQQQQPSSQV
SFQQPQQQYPSSQGSFQPSQQNPQAQGSVQPQQLPQFAEIRNLALQTLPAMCNVYIPPHC
STTIAPFGIFGTN

Avenin-like b5
QLETTCSQGFRQYQQQQQPGQRQLLEQMRPCVAFLQQQCRPL
RMPFLQTQVEQLSSCQIVQYQCCQQLAQIPEQIRCHAIHNVVEAIMQQQSQQQRQERQQQ
AQHKSMRMLLETLYLMCNIYVPIQCQQQQQLGQQQQQQLQEQLTPCATFLQHQCSPVTVP
FPQIPVDQPTSCQNVQHQCCRQLSQIPEQFRCQAIHNVAEAIRQQQPQQQWQGMYQPQQP
AQLESIRMSLQALRSMCSIYIPVQCPAPTAYNIPMVATYTGGAC

Avenin-like a2
QLYTTCSQGYGQCQQQPQPQPQPQPQMNTCAAFLQQCIQTP
YVQSQMWQASGCQLMRQQCCQPLAQISEQARCQAVCSVSQIIMRQQQGQRFGQPQQQQGQ
SFGQPQQQVPVEIMRMVLQTLPSMCSVNIPQYCTTTPCSTITPAIYSIPMTATCAGGAC

Avenin-like a3
QLYTTCSQGYGQCQQQPQPQPQMNTCAAFLQQCIQTPYVQS
QMWQASGCQLMRQQCCQPLAQISEQARCQAVCSVSQIIMRQQQGQRFGQPQQQQGQSFGQ
PQQQVPVEIMRMVLQTLPSMCSVNIPQYCTTTPCSTITPAIYSIPMTATCAGGAC

Avenin-like a4
QLDTTCSQGYGQCQQQPQQQVNTCSALLQQCSPTPYVQSQM
WQASGCQLMRQQCCQPLAQISEQARCQAVCSVAQVIMRQQQGQSFGQPQQQVQSFSQPQH
QVPIEITRMVLQTLPSMCNVNIPQYCTTTPCRTITQTPYNIPMSATCVGGTC

Avenin-like b10
QLETTCSQGFGQSQQQQQPGQRQLLEQMKPCVAFLQQKCSPL
RMPFLQTQVEQLSSCQIVQYQCCQQLAQIPERTRCHAIHIVVEAIIQQQSQQQWQEPQQQ
AQHKSMRMLLENLSLMCNIYVPVQCQQQQQLGQQQQQQLQEQLTPCTTFLQQQCSPVTVP
FPQIPVDQPTSCQNVQHQCCRQLSQIPEQFRCQAIHNVAEAIRQQQPQQQWQGMYQPQQP
AQLESIRMSLQALRSMRSIYIPVQCPAPTTYNIPLVATYTGGAC

Avenin-like b2
QLETTCSQGFGQSQQQQQPGQRQLLEQMKPCVAFLQQKCSPL
RMPFLQTQVEQLSSCQIVQYQCCQQLAQIPERTRCHAIHIVVEAIIQQQSQQQWQEPQQQ
AQHKSMRMLLENLSLMCNIYVPVQCQQQQQMGQQPQQQQLQEQLTPCATFLQHQCSPVTV
PFPQIPVDQPTSCQNVQHQCCRQLSQIPEQFRCQAIHNVAEAIRQQQPQQQWQGMYQPQQ
PAQHESIRMSLQALRSMCNIYIPVQCPAPTAYNIPMVATCTSGAC

Avenin-like b8
QLETTCSQGFGQSQQQQQPGQRQLLEQMKPCVAFLQQKCGPL
RMPFLQTQVEQLSSCQIVQYQCCQQLAQIPERTRCHAIHIVVEAIIQQQSQQQWQEPQQQ
AQHKSMRMLLENLSLMCNIYVPVQCQQQQQLGQQQQQQLQEQLTPCTTFLQQQCSPVTVP
FPQIPVDQPTSCQNVQHQCCRQLSQIPEQFRCQAIHNVAEAIRQQQPQQQWQGMYQPQQP
AQLESIRMSLQALRSMCSIYIPVQCPAPTTYNIPLVATYTGGAC

Avenin-like a6
QLYTTCSQGYGQCQQQPQPQPQPQPQPQMNTCSAFLQQCSQ
TAYVQSQMWQASGCQLMRQQCCQPLAQISEQARCQAVCSVAQIIMRQQQGQRFGQPQQQQ
GQSFSQPQQQVPVEIMGMVLQTLPSMCSVNIPQYCTTTPCSTIAPAIYNIPMTATCAGGA
C

Avenin-like b3
QLETTCSQGFGQSQQQQQPGQRQLLEQMKPCVAFLQQKCSPL
RMPFLQTQVEQLSSCQIVQYQCCQQLAQIPERTRCHAIHIVVEAIIQQQSQQQWQEPQQQ
AQHKSMRMLLENLSLMCNIYVPVQCQQQQQLGQQQQQQLQEQLTPCTTFLQQQCSPVTVP
FPQIPVDQPTSCQNVQHQCCRQLSQIPEQFRCQAIHNVAEAIRQQQPQQQWQGMYQPQQP
AQLESIRMSLQALRSMCNIYIPVQCPAPTTYNIPLVATYTGGAC

Avenin-like b6
QLETTCSQGFGQSQQQQQPGQRQLLEQMKPCVAFLQQKCSPL
RMPFLQTQVEQLSSCQIVQYQCCQQLAQIPERTRCHAIHIVVEAIIQQQSQQQWQEPQQQ
AQHKSMRMLLENLSLMCNIYVPVQCQQQQQLGQQQQQQLQEQLTPCTTFLQQQCSPVTVP
FPQIPVDQPTSCQNVQHQCCRQLSQIPEQFRCQAIHNVAEAIRQQQPQQQWQGMYQPQQP
AQLESIRMSLQALRSMCSIYIPVQCPAPTTYNIPLVATYTGGAC

Avenin-like a7
QLYTTCSQGYGQCQQQPQPQPQMNTCSAFLQQCIQTPYVQS
QMWQASSCQLMRQQCCQPLAQISEQARCQAVCSVSQIIMRQQQGQRFGQPQQQQGQSFSQ
PQQQVPVEIMRMVLQTLPSMCSVNIPQYCTTTPCSTITPAIYSIPMTATCAGGAC

Avenin-like b4
QLETTCSQGFRQYQQQQQPGQRQLLEQMRPCVAFLQQQCRPL
RMPFLQTQVEQLSSCQIVQYQCCQQLAQIPEQIRCHAIHNVVEAIMQQQSQQQRQERQQQ
AQHKSMRMLLENLSLMCNIYVPIQCQQQQQLGQQQQQQLQEQLTPCATFLQHQCSPVTVP
FPQIPVDQPTSCQNVQHQCCRQLSQIPEQFRCQAIHNVAEAIRQQQPQQQWQGMYQPQQP
AQLESIRMSLQALRSMCSIYIPVQCPAPTAYNIPMVATYTGGAC

Avenin-like b9

QLETTCSQGFGQSQQQQQPGQRQLLEQMKPCVAFLQQKCSPL
RMPFLQTQVEQLSSCQIVQYQCCQQLAQIPERTRCHAIHIVVEAIIQQQSQQQWQEPQQQ
AQHKSMRMLLENLSLMCNIYVPVQCQQQQQLGQQQQQQLQEQLTPCTTFLQQQCSPVTVP
FPQIPVDQPTSCQNVQYQCCRQLSQIPEQFRCQAIHNVAEAIRQQQPQQQWQGMYQPQQP
AQLESIRMSLQALRSMCSIYIPVQCPAPTTYNIPLVATYTGGAC

Avenin-like b11
QLDTTCSQGFRQYQQQQQPGQRQLLEQMRPCVAFLQQQCRPL
RMPFLQTQVEQLSSCQIDQYQCCQQLAQIPEQIRCHAIHNVVEAIMQQQSQQHRQERQQQ
AQHKSMRMLLETLYLMCNIYVPIQCQQQQQLGQQQQQQLQEQLTPCATFLQHQCSPVTVP
FPQIRVDQPTSCQNVQHQCCRQLSQIPEQYRCQAIHNVAEAIRHQQPQQQCQGMYQPQQP
AKLESIRMSLQALRSMCRIYIPVQCPAPTAYNIPMVATYTGGAC

Avenin-like b7
QLETTCSQGFGQSQQQQQPGQRQLLEQMKPCAAFLQQKCSPL
RMPFLQTQVEQLSSCQIVQYQCCQQLAQIPERTRCHAIHIVVEAIIQQQSQQQWQEPQQQ
AQHKSMRMLLENLSLMCNIYVPVQCQQQQQLGQQQQQQLQEQLTPCTTFLQQQCSPVTVP
FPQIPVDQPTSCQNVQHQCCRQLSQIPEQFRCQAIHNVAEAIRQQQPQQQWQGMYQPQQP
AQLESIRMSLQALRSMCSIYIPVQCPAPTTYNIPLVATYTGGAC

Gamma-gliadin
NIQVDPSGQVQWLQQQLVPQLQQPLSQQPQQTFPQPQQTFP
HQPQQQVPQPQQPQQPFLQPQQPFPQQPQQPFPQTQQPQQPFPQQPQQPFPQTQQPQQPF
PQQPQQPFPQTQQPQQPFPQLQQPQQPFPQPQQQLPQPQQPQQSFPQQQRPFIQPSLQQQ
LNPCKNILLQQSKPASLVSSLWSIIWPQSDCQVMRQQCCQQLAQIPQQLQCAAIHSVVHS
IIMQQQQQQQQQQGIDIFLPLSQHEQVGQGSLVQGQGIIQPQQPAQLEAIRSLVLQTLPS
MCNVYVPPECSIMRAPFASIVAGIGGQ

Gamma-gliadin B
NMQADPSGQVQWPQQQPFLQPHQPFSQQPQQIFPQPQQTFP
HQPQQQFPQPQQPQQQFLQPRQPFPQQPQQPYPQQPQQPFPQTQQPQQPFPQSKQPQQPF
PQPQQPQQSFPQQQPSLIQQSLQQQLNPCKNFLLQQCKPVSLVSSLWSIILPPSDCQVMR
QQCCQQLAQIPQQLQCAAIHSVVHSIIMQQEQQEQLQGVQILVPLSQQQQVGQGILVQGQ
GIIQPQQPAQLEVIRSLVLQTLPTMCNVYVPPYCSTIRAPFASIVASIGGQ

Gamma-gliadin B-I
SCISGLERPWQQQPLPPQQSFSQQPPFSQQQQQPLPQ
QPSFSQQQPPFSQQQPILSQQPPFSQQQQPVLPQQSPFSQQQQLVLPPQQQQQQLVQQQI
PIVQPSVLQQLNPCKVFLQQQCSPVAMPQRLARSQMWQQSSCHVMQQQCCQQLQQIPEQS
RYEAIRAIIYSIILQEQQQGFVQPQQQQPQQSGQGVSQSQQQSQQQLGQCSFQQPQQQLG
QQPQQQQQQQVLQGTFLQPHQIAHLEAVTSIALRTLPTMCSVNVPLYSATTSVPFGVGTG
VGAY

Gamma-gliadin
NMQVDPSGQVQWPQQQPFPQPQQPFCQQPQRTIPQPHQTFH
HQPQQTFPQPQQTYPHQPQQQFPQTQQPQQPFPQPQQTFPQQPQLPFPQQPQQPFPQPQQ
PQQPFPQSQQPQQPFPQPQQQFPQPQQPQQSFPQQQQPAIQSFLQQQMNPCKNFLLQQCN
HVSLVSSLVSIILPRSDCQVMQQQCCQQLAQIPQQLQCAAIHSVAHSIIMQQEQQQGVPI
LRPLFQLAQGLGIIQPQQPAQLEGIRSLVLKTLPTMCNVYYVPPDCSTINVPYANIDAGIG
GQ

Gamma-gliadin
NIQVDPSGQVQWPQQQPFPQPQPFSQQPQQAFLQPQHTFPL
QPQQVFPQPQQPQQQFPQPQQPQQPFPQPQQPQLPFPQQPQQPFPQPQQPQQPFPQSQQP
QQPFPQPQQQFPQPQQPQQSFPQQQPPLIQPYLQQQMNPCKNYLLQQCNPVSLVSSLVSM
ILPRNDCQVMQQQCCQQLAQIPRQLQCTAIHSVVHAIIMQQEQQGIQILRPLFQLVQGQG
IIQPQQPAQYEVIRSLVLRTLPNMCNVYVRPDCSTINAPFASIVAGIGGQ

Low-molecular-weight glutenin storage protein
QISQQQQQPPFSQQQQPQFSQQPPFSQQQQPPFSQQQQQPP

FAQQQQPPFSQQPPISQQQQPPFSQQQQPQFSQQQQPPYSQQQQPPYSQQQQPPFSQQQQ
PPFSQQQQQPPFTQQQQQQQQQQPFTQQQQPPFSQQPPISQQQQPPFSQQQQPQFSQQQQ
IPVIHPSVLQQLNPCKVFLQQQCIPVAMQRCLARSQMLQQSICHVMQQQCCQQLRQIPEQ
SRHESIRAIIYSIILQQQQQQQQQQQQQGQSIIQYQQQQPQQLGQCVSQPQQQLQQQLGQQ
PQQQQLAHGTFLQPHQIAQLEVMTSIAPRTLPTMCSVNVPLYETTTSVPLGVGIGVGVY

Gamma-gliadin
NMQVDPSGQVQWPQQQPFLQPHQPFSQQPQQIFPQPQQTFP
HQPQQQFPQPQQPRQQFLQPRQPFPQQPQQPYPQQPQRPFPQTQQPQQPFPQSKQPQQPF
PQPQQPQQSFPQQQPSLIQQSLQQQLNPCKDFLLQQCKPVSLVSSLWSIILPPSDCQVMR
QQCCQQLAQIPQQLQCAAIHSVVHSTIMQQEQQEQLQGVQILGPLSQQQQVGQGILVQGQ
GIIQPQQPAQLEVIGSLVLQTLPTMCNVHVPPYCSTIRAPFASIVASIGGQE

LMW-GS P-21
QMETRCIPGLERPWQQQPLPPQQTFPQQPLFSQQQQQQLFP
QQPSFSQQQPPFWQQQPPFSQQQPILPQQPPFSQQQQLVLPQQPPFSQQQQPVLPPQQSP
FPQQQQQHQQLVQQQIPVVQPSILQQLNPCKVFLQQQCSPVAMPQRLARSQMLQQSSCHV
MQQQCCQQLPQIPQQSRYEAIRAIIYSIILQEQQQVQGSIQSQQQQPQQLGQCVSQPQQQ
SQQQLGQQPQQQQLAQGTFLQPHQIAQLEVMTSIALRILPTMCSVNVPLYRTTTSVPFGV
GTGVGAY

Alpha gliadin
VRVSVPQLQPQNPSQQQPQEQVPLVQQQQFLGQQQPFPPQ
QPYPQLQPFPSQQPYLQLQPFPQPQLPYSQPQPFRPQQPYPQPQPQYSQPQQPISQQQQQ
QQQQQQQQQQQQQQQQILQQILQQQLIPCMDVVLQQHNIVHGRSQVLQQSTYQLLRELCC
QHLWQIPEQSQCQAIHNVVHAIILHQQQKQQQQPSSQVSFQQPLQQYPLGQGSFRPSQQN
PQAQGSVQPQQLPQFEEIRNLALQTLPAMCNVYIPPYCTIAPFGIFGTN

Alpha-gliadin
VRVPVPQLQPQNPSQQQPQEQVPLVQQQQFLGQQQPFPPQ
QPYPQPQPFPSQQPYLQLQPFLQPQLPYSQPQPFRPQQPYPQPQPQYSQPQQPISQQQQQ
QQQQQQQQQQQQQQIIQQILQQQLIPCMDVVLQQHNIVHGKSQVLQQSTYQLLQELCCQH
LWQIPEQSQCQAIHNVVHAIILHQQQKQQQQPSSQVSFQQPLQQYPLGQGSFRPSQQNPQ
AQGSVQPQQLPQFEEIRNLALQTLPAMCNVYIPPYCTIAPFGIFGTN

Omega gliadin storage protein
ARELNPSNKELQSPQQSFSYQQQPFPQQPYPQQPYPSQQPY
PSQQPFPTPQQQFPEQSQQPFTQPQQPTPIQPQQPFPQQPQQPQQPFPQPQQPFPWQPQQ
PFPQTQQSFPLQPQQPFPQQPQQPFPQPQLPFPQQSEQIIPQQLQQPFPLQPQQPFPQQP
QQPFPQPQQPIPVQPQQSFPQQSQQSQQPFAQPQQLFPELQQPIPQQPQQPFPLQPQQPF
PQQPQQPFPQQPQQSFPQQPQQPYPQQQPYGSSLTSIGGQ

Low-molecular-weight glutenin storage protein
QMETRCIPGLERPWQQQPLPPQQTFPQQPLFSQQQQQQLFP
QQPSFSQQQPPFWQQQPPFSQQQPILPQQPPFSQQQQLVLPQQPPFSQQQQPVLPPQQSP
FPQQQQQHQQLVQQQIPVVQPSILQQLNPCKVFLQQQCSPVAMPQRLARSQMLQQSSCHVM
QQQCCQQLPQIPQQSRYEAIRAIIYSIILQEQQQVQGSIQSQQQQPQQLGQCVSQPQQQS
QQQLGQQPQQQQLAQGTFLQPHQIAQLEVMTSIALRILPTMCSVNVPLYRTTTSVPFGVG
TGVGAY

High molecular weight glutenin subunit EGEASGQLQCERELQEHSLKACRQVVDQQLRDVGPECQP
VGGGPVARQYEQQVVVPPKGGSFYPGETTPPQQLQQSILWGVPALLRRYYLSVASPQQVS
YYPGQASSRRPGQGQQEYYLTSPQQSGQWQQPGQGQSGYYPTSPQQSGQKQPGYYPTSPW
QPEQLQQPTQGQQRQQPGQGQQLRQGQQGQQSGQGQPRYYPTSSQQPGQLQQLVQGQQGQQPERGQ
QGQQSGQGQQLGQGQQGQQPGQKQQSGQGQQGYYPISPQQLGQGQQSGQGQLGYYPTSPQQSGQGQ
SGYYPTSAQQPGQLQQSTQEQQLGQEQQDPQSGQGRQGQQSGQRQQDQQSGQGQQPGQRQPGYYSTS
PQQLGQGQPRYYPTCPQQPGQEQQPRQLQQPEHGQQGQQPEQGQQGQQQRQGEHGQQPGQGQQGQQ
PGQGQPGYYPTSPQQSGQGQPGYYPTSPQQSGQLQQPAQGQQPGQEQQGQQPGQGQQPGQGQPGYYP
TSPQQPGQEQQLEQWQQSGQGQPGHYPTSPLQPGQGQPGYYPTSPQQIGQGQQPGQLQQPTQGQQGQ
QPGQGQQGQQPGEGQQGQQPGQGQQPGQGQPGYYPTSLQQSGQGQQPGQWQQPGQGQPGYYPTSSL

QPEQGQQGYYPTSQQQPGQGPQPGQWQQSGQGQQGYYPTSSQQSGQGQQPGQWLQPGQWLQSGYYL
TSPQQLGQGQQPRQWSQPRQGQQGYYPTSPQQSGQGQQLGQGQQGYYPTSPQQSGQGQQGYDSPYY
VSAEHQAASLKVAKAQQLAAQLPAMCRLEGGDALLASQ

Low-molecular-weight glutenin storage protein
QMETSCIPGLERPWQQQPLQQKETFPQQPPSSQQQQPFPQQ
PPFLQQQPSFSQQPLFSQKQQPVLPQQPAFSQQQQTVLPQQPAFSQQQHQQLLQQQIPIV
HPSILQQLNPCKVFLQQQCSPVAMPQHLARSQMWQQSSCNVMQQQCCQQLPRIPEQSRYE
AIRAIIFSIILQEQQQGFVQPQQQQPQQSVQGVYQPQQQSQQQLLQCSFQQPQQQLGQQP
QQQQVQKGTFLQPHQIARLEVMTSIALRTLPTMCSVNVPLYSSITSAPLGVGSRVGAY

Alpha-gliadin storage protein
VRFPVPQLQPQNPSQQLPQEQVPLVQQQQFLGQQQPFPPQ
QPYPQPQPFPSQLPYLQLQPFPQPQLPYSQPQPFRPQQPYPQPQPQYSQPQQPISQQQQQ
QQQQQQQQQQQQQILQQILQQQLIPCMDVVLQQHNIAHGRSQVLQQSTYQLLQELCCQHL
WQIPEQSQCQAIHNVVHAIILHQQQKQQQQPSSQVSFQQPLQQYPLGQGSFRPSQQNPQA
QGSVQPQQLPQFEEIRNLALQTLPAMCNVYIPPYCTIAPFGIFGTN

Low-molecular-weight glutenin storage protein
QMETSCISGLERPWQQQPLPPQQSFSQQPPFSQQQQQPLPQ
QPSFSQQQPPFSQQQPILSQQPPFSQQQQPVLPQQSPFSQQQQLVLPPQQQQQQLVQQQI
PIVQPSVLQQLNPCKVFLQQQCSPVAMPQRLARSQMWQQSSCHVMQQQCCQQLQQIPEQS
RYEAIRAIIYSIILQEQQQGFVQPQQQQPQQSGQGLSQSQQQSQQQLGQCSFQQPQQQLG
QQPQQQQQQVLQGTFLQPHQIAHLEAVTSIALRTLPTMCSVNVPLYSATTSVRFGVGTGV
GAY

Alpha-/beta-gliadin storage protein VRVPVPQLQPQNPSQQQPQEQVPLVQQQQFPGQQQPFPPQ
QPYPQPQPFPSQQPYLQLQPFPQPQLPYPQPQPFRPQQPYPQPQPQYSQPQQPISQQQQQ
QQQQQQQQQILQQILQQQLIPCMDVVLQQHNIAQGRSQVLQQSTYQLLQELCCQHLWQIP
EQSQCQAIHNVVHAIILHQQHHHHQQQQQQQQQQPLSQVSFQQPQQQYPSGQGFFQPSQQ
NPQAQGSFQPQQLPQFEAIRNLALQTLPAMCNVYIPPYCTIAPFGIFGTN

Alpha-gliadin storage protein
VRWPVPQLQPQNPSQQQPQEQVPLVQQQQFLGQQQPFPPQ
QPYPQPQPFPSQQPYLQLQPFSQPQLPYSQPQPFRPQQPYPQPQPQYSQPQQPISQQQQQ
QQQQQQQQQQQQEQQILQQILQQQLTPCMDVVLQQHNIARGRSQVLQQSTYQLLQELCCQ
HLWQIPEKLQCQAIHNVVHAIILHQQQQKQQQPSSQVSFQQPQQQYPLGQGSFRPSQQNP
QAQGSVQPQQLPQFEEIRNLALQTLPAMCNVYIPPYCTIAPFGIFGTN

Alpha-/beta-gliadin storage protein
VRVPVPQLQPQNPSQQQPQEQVPLVQQQQFLGQQQPFPPQ
QPYPQPQPFPSQQPYLQLQPFPQPQLPYPQPQPFRPQQPYPQPQPQYSQPQQPISQQQQQ
QQQQQQQQQQILQQILQQQLIPCMDVVLQQHNIVHGRSQVLQQSTYQLLRELCCQHLWQI
PEQSQCQAIHNVVHAIILHQQQKQQQQPSSQVSFQQPLQQYPLGQGSFRPSQQNPQAQGS
VQPQQLPQFEEIRNLALQTLPAMCNVYIPPYCTIAPFGIFGTN

Storage protein
SHDDEDDRRGGHSLQQCVQRCRQERPRYSHARC
VQECRDDQQQHGRHEQEEEQGRGRGWHGEGEREEEHGRGRGRHGEGEREEEHGRGRGRHG
EGEREEERGRGHGRHGEGEREEERGRGRGRHGEGEREEEEGRGRGRRGEGERDEEQGDSR
RPYVFGPRSFRRIIQSDHGFVRALRPFDQVSRLLRGIRDYRVAIMEVNPRAFVVPGFTDA
DGVGYVAQGEGVLTVIENGEKRSYTVKEGDVIVAPAGSIMHLANTDGRRKLVIAKILHTI
SVPGKFQFLSVKPLLASLSKRVLRAAFKTSDERLERLFNQRQGQEKTRSVSIVRASEEQL
RELRREAAEGGQGHRWPLPPFRGDSRDTFNLLEQRPKIANRHGRLYEADARSFHALANQD
VRVAVANITPGSMTAPYLNTQSFKLAVVLEGEGEVQIVCPHLGRESESEREHGKGRRREE
EEDDQRQQRRRGSESESEEEEEQQRYETVRARVSRGSAFVVPPGHPVVEISSSQGSSNLQ
VVCFEINAERNERVWLAGRNNVIGKLGSPAQELTFGRPAREVQEVFRAQDQDEGFVAGPE
QQSREQEQEQERHRRRGDRGRGDEAVETFLRMATGAI

Seed storage protein

NMQVDPSGQVPWPQQQPFPQPHQPFSQQPQQTFPQPQQTFP
HQPQQQFSQPQQPQQQFIQPQQPFPQQPQQTYPQRPQQPFPQTQQPQQPFPQSQQPQQPF
PQPQQQFPQPQQPQQSFPQQQPSLIQQSLQQQLNPCKNFLLQQCKPVSLVSSLWSMILPR
SDCQVMRQQCCQQPAQIPQQLQCAAIHSIVHSIIMQQEQQEQRQGVQILVPLSQQQQVGQ
GTLVQGQGIIQPQQPAQLEVIRSLVLQTLATMCNVYVPPYCSTIRAPFASIVAGIGGQ

High-molecular-weight glutenin
EGEASEQLQCDRELQELQERELKACQQVMDQQLRDISPECHPVVVSPVAGQYEQQIVVP
PKGGTFYPGETTPPQQLQQRIFWGIPALLKRYYPSVTCPQQVSYYPGQASPQRSRDITSS
SYHVSVEHQAASLKVAKAQQLAAQLPAMCRLEGGDALSASQ

Alpha/beta-gliadin storage protein VRVPVPQLQPKNPSQQQPQEQVPLVQQQQFPGQQQQFPPQQPY
PQPQPFPSQQPYLQLQPFPQPQPFLPQLPYPQPQSFPPQQPYPQQRPMYLQPQQPISQQQ
AQQQQQQQQQQQQQQQQQILQQILQQQLIPCRDVVLQQHNIAHASSQVLQQSTYQLLQQL
CCQQLLQIPEQSRCQAIHNVVHAIIMHQQEQQQQLQQQQQQQLQQQQQQQQQQQQPSSQV
SFQQPQQQYPSSQGSFQPSQQNPQAQGSVQPQQLPQFAEIRNLALQTLPAMCNVYIPPHC
STTIAPFGIFGTN

Alpha-gliadin storage protein
VRVPVPQLQPKNPSQQQPQEQVPLVQQQQFPGQQQQFPPQQPY
PQPQPFPSQQPYLQLQPFPQPQPFLPQLPYPQPQSFPPQQPYPQQRPKYLQPQQPISQQQ
AQQQQQQQQQQQQQQQQQILQQILQQQLIPCRDVVLQQHNIAHASSQVLQQSTYQLLQQL
CCQQLLQIPEQSRCQAIHNVVHAIIMHQQEQQQQLQQQQQQQLQQQQQQQQQQQQPSSQV
SFQQPQQQYPSSQVSFQPSQLNPQAQGSVQPQQLPQFAEIRNLALQTLPAMCNVYIPPHC
STTIAPFGIFGTN

supplementary file 4

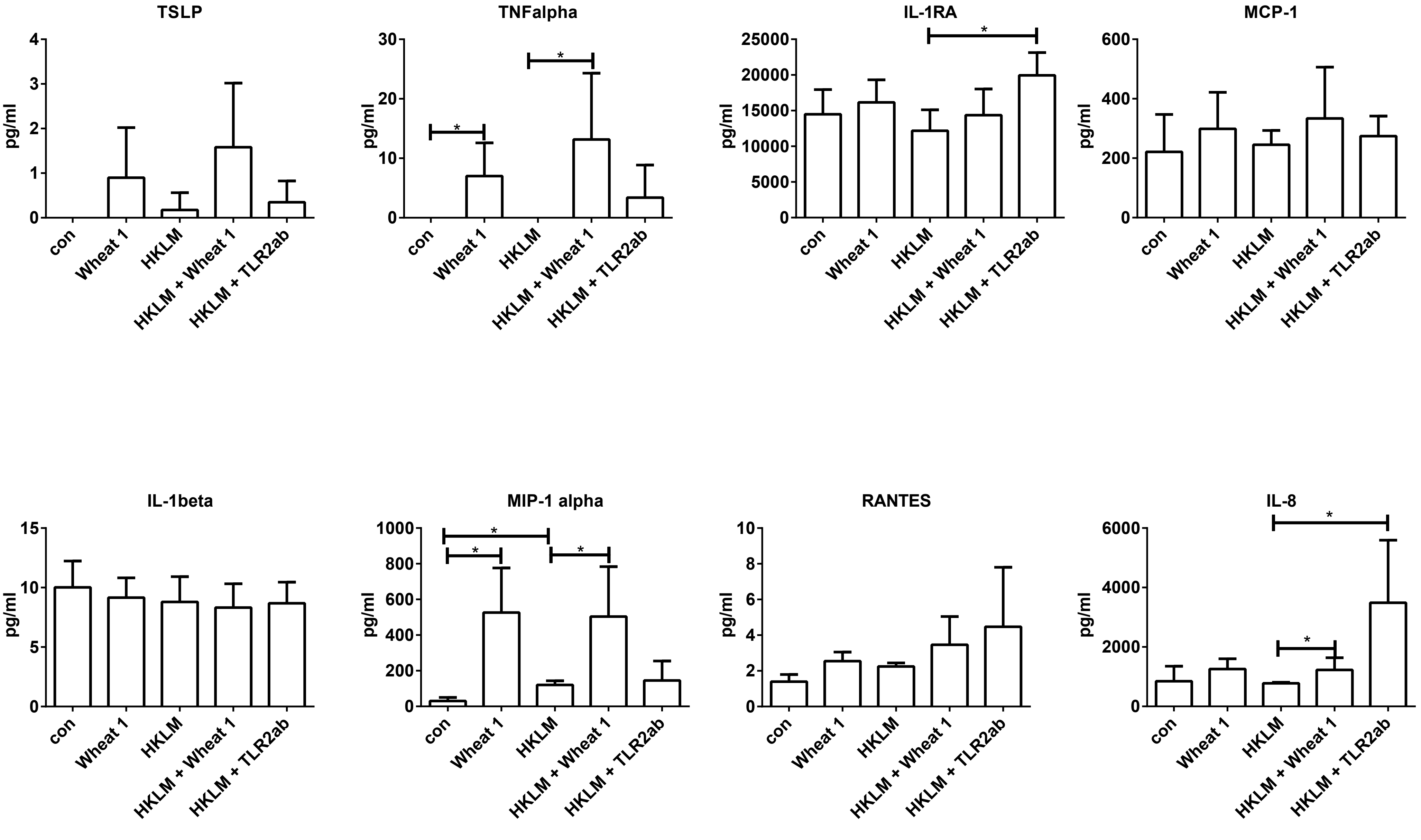

Supplementary file 5:

<0.5 kD
AAFKTSDERL
AAIHSVAH
AAIHSVAHSIIMQ
AEAIRQQQPQQQWQGMYQ
AEAIRQQQPQQQWQGMYQ
AGRNNVIGKLGSP
AHARSQV
AHASSQVLQQSS
AIHSIVH
AIHSVAHSIIMQ
AIHSVVEAIMQQQSQ
AIILHQ
AIILHQQ
AIILHQQ
AIIMHQQ
AIIMHQQEQQ
AIIMHQQQQ
AIM
AIQSFLQQ
AIYNIPM
AIYSIPMTA
ALETL
ALETLPAM
ALQTLPAMCNVYIPPYCTIAPF
ALRSM
AMCNVYIPPHCSTTIAPFGI
AMPQRLARSQ
ANRHGRLYEADA
ANRHGRLYEADARSFHALA
APFASI
APFASIV
APFASIVA
APFASIVAG
APFASIVAGIGGQ
APTAYNIPMVATY
APYLNTQSFK
AQGLGIIQPQQPAQLEG
AQGTFL
AQGTFLQPHQIAQ
AQGTFLQPHQIAQL
AQIIMRQQ
AQIIMRQQQ
AQIPERT
AQIPQQL
AQVIMRQQQ
ARCQAVCSVA
ASIVAGIGGQ
ASLVSSLW
AVANITP

AVCGVAQ
AVCGVAQVI
AVCSVA
AVCSVSQIIM
AVTSIA
AVVLEGEGEV
CAAIHSIVHSI
CCQQLRQIPEQSR
CKNFLLQQCKPVSLVSS
CQAIHNVA
CQQLLQIP
CQQPQRTIP
CSVNVPLYR
CSVSQIIMRQ
CTIAPFGI
CTIAPFGIF
CTTTPCSTIAPAIYNIPMTAT
CVAFLQQKCGPL
DCSTINAPFASIV
DDQRQQRR
DIFLPLSQH
DPSGQVPW
DPSGQVPWPQQQPFPQ
DREL
DVVLQ
DVVLQQH
EAIIQ
EAIIQQQSQQQW
EASEQLQCDRELQELQERELKACQQVMDQQLRDISP
ECHPVVVSPVAGQYEQ
EHGRGRGRHGEGE
EIRNLALQT
EITRMVLQ
EQGDSRRPYV
EQMKPCVA
EQMKPCVA
EQSRH
ESTYQLV
EVMTSIA
EVMTSIALR
FGQPQQQVPVE
FGQYQQQQ
FGVGTGV
FIQPQQPFPQQP
FIQPSL
FIQPSLQQ
FIQPSLQQQV
FLQPHQIAQL
FLQPQQPFPQQP
FLQQQCRPL
FLQTQVEQLSSCQIVQY
FPGQQQPFP

FPPQLPY
FPQIRVD
FPQPQPFL
FPQPQQPQQQF
FPQPQQPQQS
FPQPQQPQQS
FPQPQQPQQSFPQQQQ
FPQQPLFSQ
FPQQPQQPYPQQPQQPFPQTQQPQQ
FPQSQQPQQQ
FPSQQPYMQLQPFPQ
FQQPLQQ
FRPQQS
FRQY
FRRIIQSD
FSQPQLPYSQ
FSQPQQPQQQ
FSQPQQPQQQFIQPQQPFPQQPQQTYPQRPQ
FSQPQQQFPQPQQPQQ
FSQQPPISQQQQP
FSQQPQQAFLQ
FSQQQPIL
FSQQQQQPLPQQPSFSQQQPPFSQQQ
FTDADGVGY
FVVPPGHPVVEIS
GCQLVR
GCQLVRQQ
GDRGRGDEAV
GEGEVQIVCPHLG
GEGVLTVIENG
GFRQ
GFVQPQQQ
GIIQPQQP
GPLRMPFLQTQVEQLSSCQIVQYQCCQQLAQIP
GPLSQQQQVG
GQGHRWPL
GQGIIQPQQPAQL
GQGTLVQGQGI
GQQQPFPPQ
GQVQWLQ
GQVSFQSSQQN
GRESESE
GRGRGRH
GRGWH
GSIMHLA
GSLVQGQGIIQ
GSSQVLQQSTYQLVQQLCCQQLWQIPE
GSVQPQQLP
GSVQPQQLPQ
GVAQVI
GVGYVAQ
HAIIMHQQEQQQQLQQQQQQQ

HAIIMQQEQQG
HALANQDV
HARCV
HAVCGVA
HCSTTIAPFGIF
HESIRMSLQALRSMCNIYI
HGRLYEADAR
HGSSQVLQQS
HHQQQQQQQQQQPL
HILL
HKSMR
HKSMRML
HLAN
HLANT
HNIAHGRS
HNIVH
HNIVHGK
HPSVLQ
HPSVLQQL
HQQLV
HQQQRQQ
HRRRG
HSIIMQQQQ
HSVAHSI
HSVVEAI
HSVVHA
HSVVHAIIM
HSVVHAIIMQQ
HTIIH
HVMQQQCCQQLQQIPEQ
HVPPYCSTIR
HVQTQM
HVSVEH
IAHASS
IAPA
IAPFGI
IAPFGIF
IAPFGIFGTN
IAPFGIS
IAPVGIF
IAPVGIFGTN
IENGEKRSY
IFGT
IFWGIPA
IGKLGSPAQ
IHSIV
IHSIVH
IHSVVHSTIMQQEQQEQ
IILHQQ
IILQEQQQGF
IILQQQ
IIM

IIMHQQEQQ
IIMQ
IIMRQ
IIMRQQQGQSFGQPQ
IIQPQQPAQ
IIQPQQPAQL
ILGPLSQ
ILHQQ
ILQEQQQVQG
ILQQIL
ILQQQL
ILQQQL
ILSQQPPFSQ
ILVQGQGI
IMGMV
IMQQQQ
IMRAPFAS
INAPFAS
IPCRDVVLQQHSIAHGSSQVLQQSTYQLVQQFC
IPEQSR
IPERT
IPLVAT
IPMTAT
IPPYCTIAPFGI
IPQPHQT
IPVIH
IPVQCPAP
IPVQCPAPTTYNIPL
IPVVQ
IPVVQP
IQCQQQQQLGQ
IQPSL
IQPSLQ
IQPSLQQQ
IQPYLQQ
IQPYLQQQ
IQVDPSGQVQW
IRCHAIH
IRHQQ
ISQQQQQPPF
ITQTPYNI
ITRMVLQTL
IVASIGGQ
IVASIGGQ
IVHGRSQVLQQST
IVQPSVL
IVVEAIIQQQSQQQWQEPQQ
IVVPPKGG
KCGPLRMP
KFQF
KNPSQQ
KNPSQQQP

KPCAAFLQ
KRSYTV
KTSDERLERLFNQ
KVAKAQQLAA
LALETLPAM
LALQT
LALQTL
LAQGLGIIQPQQPAQL
LAQGLGIIQPQQPAQLEG
LAQGTFLQP
LAQIPERT
LAQISE
LATMCNVYVPPYCSTIRAPFASI
LEQMKPCVAFLQQKCSPLRMPFLQ
LEQMRPCVAFL
LESIRMSL
LEVMTS
LEVMTSIALR
LFQLVQGQG
LGSPAQELT
LHQQ
LIPCRDVVLQQHNIAHGSSQVLQESTYQ
LIQPYLQ
LIQPYLQQ
LLEQRP
LLPLYQQ
LLQIPEQSQ
LLQQSKP
LLRELCCQHLWQIPEQSQCQAIHNV
LMQQQQ
LNPCKN
LNPCKN
LPFPQQ
LPYSQPQPFRPQQPYPQPQPQYSQPQQ
LQELQERE
LQEQQQVQ
LQIPEQSR
LQLQPFSQPQLPYSQPQPFRPQQPYPQ
LQNPSQQQPQEQVPLVQEQ
LQPFL
LQPHQ
LQPHQPF
LQPRQPFPQQPQQPY
LQQHNIAHG
LQQHSIAYGS
LQQILQ
LQQPLS
LQQPLSQ
LQQPLSQQ
LQQSTYQ
LQQSTYQLLQE
LQTQVEQ

LQVVC
LQVVCF
LRIL
LRMPFLQTQ
LRMPFLQTQ
LRPFDQ
LRTLPNM
LSLM
LSLM
LSQIP
LSQQQQVGQGILVQGQG
LVLPQQPPF
LVLRT
LVPQLQQ
LVQGQGI
LVQGQGIIQPQQPAQL
LVQQLCCQQL
LVQQQQF
LVRQ
LVSSLWSMIWPQ
LYQQ
MHILLP
MLLETLYL
MQLQPFP
MRML
MRMLLEN
MRMLLETLYLMCNIYVP
MTAPYLN
MVLQT
NHVSLVS
NIAHASSQ
NIAHGRSQ
NIQVDP
NIQVDPSGQVQW
NIYVPV
NIYVPVQ
NIYVPVQCQQQQQLG
NLALETL
NLALETLP
NLSLMCNIYVPVQCQQ
NMQVDPSGQVQWPQQQ
NMQVDPSSQ
NVAHA
PAIQSF
PAMCNVYIPPYCSTTIAP
PAPTTYNIP
PAPTTYNIPL
PASLVSSL
PCVAFLQQKCSPL
PERTR
PFASIVAGIGG
PFGVGTGVGA

PFIQPSLQ
PFIQPSLQ
PFIQPSLQQ
PFIQPSLQQQ
PFLPQL
PFLPQLPY
PFLQPQLPYSQPQPF
PFPPQLPY
PFPQPQLPYPQPQLPY
PFPQPQQQFPQPQQPQQS
PFPSQLPYL
PFPSQQPYMQLQPFPQPQLPYP
PFSQQQQLV
PFTQQQQQQQQ
PGQQEQFPPQQPYPHQQP
PGQRQLLEQ
PGQRQLLEQMRPCVAFLQQQC
PHQIAHLEAVTSIA
PHQPF
PHQPQQQFPQ
PISQQ
PLAQISEQAR
PLFQL
PLFQLVQGQ
PLGQGSFR
PLGVGI
PLMQQ
PPFA
PPFWQQQPPF
PPQLPYPQTQ
PPQLPYPQTQPFPP
PPQQPY
PPQQPYPQPQPFPSQLPYLQLQPF
PPYCTIAPFGIFGTN
PQAQGSFQPQQ
PQAQGSVQP
PQFQEIRNL
PQFSQQPPFSQQ
PQHTF
PQIRVD
PQLPYPQPPPF
PQLQLQNPS
PQLQLQNPSQQ
PQLQLQNPSQQQ
PQLQQPLSQQP
PQPFLPQL
PQPFPSQQPYLQLQPFPQPQLPYP
PQPHQPFSQQPQQ
PQPQLPY
PQPQQPIS
PQQLPQFQ
PQQLQQR

PQQPAQL
PQQPAQLE
PQQPAQLES
PQQPF
PQQPFPQQPQQP
PQQPQQPYPQQPQRP
PQQPQQTYPQR
PQQPSFSQQQPPF
PQQPYPQPQPFPS
PQQQFPQPQQ
PQQQFPQPQQPQQPFPQPQQPQLPFP
PQQQPFLQPHQ
PQQQPFPQPQ
PQQQPFPQPQQP
PQQQPFPSQQ
PQQQPSLIQ
PQQQQPAIQSFLQ
PQQQQPAIQSFLQQQMN
PQQQRPFI
PQQRPK
PQQSFP
PQQSFPQQQQ
PQQSFPQQQR
PQQSRYEAIR
PQQTFPQQPQLP
PQRLARSQM
PQSFPPQ
PQSQPQYSQPQQPISQQ
PQTQPFPP
PRYSHARCV
PSGQVQW
PSGQVQW
PSGQVQWPQ
PSLIQQ
PSLQQ
PSLQQQLNP
PSQLNP
PSQLNPQA
PSQQNPQAQGSFQPQQLPQFEEIRNLA
PSQQQPQKQVPLVQQQ
PTMCSVNVPLYSATTSVPFGV
PVAGQYEQQ
PVAMPQR
PVEIMGMVLQTL
PVLPPQQSP
PVPQPQPQN
PVQCPAPTA
PVQCQQQQQLGQQ
PVVEISSS
PVVVSPVAGQYEQQ
PWQQQ
PWQQQPLPP

PYCSTIRAPFASIVASIGG
PYCTI
PYLN
PYLQLQPFPQPQPFPPQLPYPQPPPFSP
PYPQQRPKYLQ
PYPQQRPMY
PYVQ
PYVQS
QAFLQPQ
QARCQAVCSVAQIIMRQQQG
QCAAIHSVVHSTIMQQEQQEQLQGVQ
QCAAIHTIIHS
QCAAIHTIIHSII
QCCQQLAQIPQQ
QCCQQLAQIPQQL
QCKPVSLVSSLWSM
QELTFGRP
QEQK
QEQLTPCATFLQHQCSPVT
QEQQQGVP
QEQVPLMQQQQ
QERELKACQQVMDQQLRD
QEVFRA
QFCCQQLWQIP
QFEIRN
QFIQPQQ
QFIQPQQP
QFLGQQQ
QFLQPQQP
QFPGQQQQFPP
QFPQPQQPQQSFPQQQPP
QGDSRRPYVFGPRSF
QGFRQYQQQQQPGQR
QGHRWPLPPFRGDSRDT
QGQGI
QGQGI
QGQGIIQPQ
QGQQQPFPP
QGQSI
QGQSIIQ
QGSFQPSQQNPQAQGSVQPQ
QGSIQ
QGSVQPQQLPQ
QGTFLQPHQI
QHLWQI
QHLWQI
QHLWQIPEQSQC
QHNIVHGR
QHQVP
QHRQ
QIFPQ
QILGPLSQQQ

QIPERT
QIPVVQ
QIPVVQP
QIRVDQPT
QIVCPHLGR
QIVQHQCCQQLAQIPER
QLESIRMSLQ
QLEVIR
QLFPQ
QLFPQSQQ
QLLQI
QLMRQ
QLPYPQT
QLQCAAIHSVVHSI
QLQCTAI
QLQEQLTPCAT
QLQNPSQQQ
QLQPF
QLQPKN
QLQPQN
QLSQIP
QLVPQL
QNPQAQGSVQPQQL
QPAQLE
QPFPQPQLPYSQPQPFRPQQ
QPFPQPQPFL
QPFPQPQPFPP
QPFPQPQQPQQPFPQSQQ
QPFPQPQQTF
QPFPQSK
QPLAQISEQARCQAVCSVAQVIM
QPLPPQQS
QPLPPQQTFPQ
QPPFIQPSLQQ
QPPFSQQPPIS
QPPFSQQQPIL
QPPFSQQQPILS
QPQEQVPLVQQQQFPGQQQQF
QPQFSQQPPFS
QPQGQV
QPQHQVP
QPQHTFPL
QPQQIFPQPQQTFPHQPQQQFPQPQQP
QPQQLPQFQ
QPQQPAK
QPQQPFPQQP
QPQQSGQGVS
QPQQTYPQRPQQP
QPQSFP
QPRQQFLQPRQPFPQQ
QPVLPPQQSPFPQQQQQH
QPYPQPQP

QPYPQQR
QQEQLQGVQILGPLSQQ
QQFIQPQQP
QQFPGQQQPFPP
QQFSQPQ
QQHNIAHGSSQVLQEST
QQHNIARG
QQHNIVHGR
QQILQQ
QQILQQILQQQLIPCMD
QQIPEQSR
QQLAAQLPAMCRLEGG
QQLAQIP
QQLGQQ
QQLPQFEEIRNLALQTLPAMCNV
QQLQCAAIHSVVHSIIMQQEQQEQLQ
QQLR
QQPAQLE
QQPAQLES
QQPAQLESIRMSLQALRSMCNI
QQPFLQPH
QQPFPQPQQQF
QQPFPQQPQQ
QQPFPQQPQQPYPQQPQQPFPQTQQ
QQPFPS
QQPILPQQPPFSQQ
QQPILSQQP
QQPLFSQQ
QQPLPPQQ
QQPLSQV
QQPPFS
QQPPFSQQQQLVLP
QQPPISQQQQPPFSQ
QQPQQQQLAQGTFLQPHQI
QQPQQQQQQQVLQGTFLQ
QQPQQQQQQQVLQGTFLQPHQ
QQPQQTF
QQPQQTF
QQPQQTFPQPQQTFP
QQPRQQF
QQPVLPQQS
QQPVLPQQSPFSQQQQLVLPPQQQQQQL
QQPYLQLQPFS
QQPYPH
QQQCI
QQQGIDIFL
QQQLIPCR
QQQLVPQLQQ
QQQMNPC
QQQPFLQ
QQQPFPQPQQ
QQQPILPQ

QQQPILPQQ
QQQPPFSQQQPI
QQQPQQQV
QQQPSLIQ
QQQQGIDI
QQQQIL
QQQQLF
QQQQLGQQQQQQLQEQLTPCTT
QQQQPGQRQLL
QQQQPGQRQLL
QQQQPLPQQ
QQQQPP
QQQQPQQLG
QQQQQGIDIFLPLSQHEQVGQGSLVQGQ
QQQQQPLSQVCF
QQQQQQQPSSQVSFQQPQ
QQQQQQQQQPLSQVSFQQPQQQYPSG
QQQQVLQG
QQQRQQQPSS
QQQVLQGTFLQP
QQQVPQ
QQQVQGSIQS
QQRPF
QQRQERQQQAQH
QQSTYQPL
QQTFPHQPQQQFPQP
QQVGQGILVQGQG
QQVPVEIMGM
QRCLARS
QRQL
QRSRDIT
QSFK
QSIIQYQQQQ
QSIIQYQQQQPQQLGQCVSQPQQQ
QSTYQLLRE
QSTYQLVQQLC
QTFPHQPQQQFP
QTLPAM
QTPH
QVEQLSSCQIDQ
QVFPQP
QVGQGI
QVLQGTFLQPH
QVPLVQ
QVQGSIQSQ
QYPLGQ
QYQCCQQLAQIPER
RAAF
RDVVLQQ
RDVVLQQ
RDVVLQQHNIAHAR
RDVVLQQHS

RDYRVA
REEERGRGR
REEERGRGRGRHGEG
REVQEVFR
RGDRGRGDEAV
RGRGRHGE
RGRGWH
RHGRLY
RHQQP
RLFNQRQGQEKTRS
RLYEAD
RLYEADARSFHA
RMLLEN
RNERVW
RPFIQ
RPLFQ
RQLQCT
RQQQGQRFGQPQQQQGQSFSQPQQQVPV
RSLVLKTLPTM
RTLPNMCNVYVRPDCST
RTRCHA
RVAVAN
RVWLAGR
SAFLQQCIQTP
SAFVVPPG
SFLQQQMNPC
SFRPSQQN
SIAHGSSQ
SIAHGSSQVL
SIAYGSSQ
SIIM
SIIMQ
SIIMQQ
SIIMQQE
SIIMQQE
SIIWPQSD
SIRMSLQALRS
SIVAGIGGQ
SIYIPVQCPAPTAY
SKPASL
SKQPQQPF
SLVQGQGI
SLVQGQGIIQPQ
SLVSSL
SLVSSLV
SLVSSLWSMI
SLWSI
SMIL
SQPQPQGQV
SQQPYLQLQPF
SQQQQPVLPQQSPFSQQ
SQQQQQPGQRQL

SQQQQQQQQQQQQQQQEQQILQQILQQQLI
SQQQQVGQGIL
SQQQRQERQ
SQTAYVQSQMWQASGCQLMR
SQVLQQSSYQQL
SQVLQQSTYQLVQQLCCQQLWQIPEQSRCQAI
SQVSFQPSQ
SRYEAI
SSLVSI
SSNLQVVCFEINA
SSQVQWPQQQPV
STIAP
STIRAPFASIVAG
SVAHSIIMQ
SVAHSIIMQQ
SVAQIIMR
SVLQQLNPCKV
SVNIPQYCTTTPCSTITPAIYSIP
SVQPQQLPQ
SVQPQQLPQFAEIR
SVVHSTIMQ
SYHVSVEHQAASLKV
TCSALLQQCSPTPYV
TFGRPAR
TFPHQ
TFPHQ
TIAPAIY
TIMQQEQQ
TINVPYANID
TIRAPFASIV
TITPAIYSIPMT
TLPRMCNVYIPP
TLQQI
TPCSTIAPAI
TPCTTFL
TQVEQLS
TQVEQLSSCQIVQHQCCQQL
TSIAP
TTIAPFGI
TTIAPFGIF
TTIAPFGIFGTN
TTIAPFGIFGTN
TTTSVPFGV
TTTSVPFGVGTGVG
TTTSVPFGVGTGVGA
TTTSVPFGVGTGVGAY
TTTSVPL
TYQL
VAFLQQKCSPLRMPFL
VAKAQQL
VAQIIM
VCGVAQVI

VCPHLGRES
VCSVAQI
VCSVSQIIMRQ
VDPSSQVQWPQQ
VEIMGMVL
VEIMRM
VEQLSSCQIVQH
VFPQPQQP
VFR
VGQGIL
VGQGSLVQGQG
VHSIIMQQQQQQQ
VHSTI
VLPPQQQ
VLQQH
VLQQHNIVHG
VLQQSSY
VLQQSSYQQLQQ
VLQQST
VLQQSTYQ
VLQQSTYQL
VLQQSTYQLLQQLC
VLQTLPLM
VMTSIA
VNPCKNFLL
VNTCSALL
VNVPLY
VNVPLYR
VPLSQQQ
VPLSQQQQVGQGT
VPLVQ
VPLYETTTSV
VPLYSATTSVPFGVG
VPPGHP
VPPYCSTIRAPFAS
VPPYCSTIRAPFAS
VPQLQLQ
VPVPQL
VPVPQLQ
VPVPQLQPQNPSQQQPQEQ
VPWPQQQP
VQGQGI
VQGQGIIQPQQPAQL
VQGQGIIQPQQPAQY
VQGSIQSQ
VQHQCCQQLAQI
VQWL
VRAL
VRF
VSFQQPQQQYPSSQG
VSLVSS
VSLVSSLVSM

VSPVAGQY
VSRGSAFVVP
VSRGSAFVVPP
VSSLW
VVHA
VVHAIILHQQQQKQQQQPSSQFSFQQ
VVHAIIMHQ
VVLQQH
VWLAG
WPQQQ
WPQQQPFL
WQIPEQSR
WQQQPP
WSIIWPQSD
YETTTSVPLGVG
YGQCQQQPQQQVNTCSALLQQCSPTPYVQSQMWQASGCQL
YIPPHCSTTI
YLLQQ
YLNTQSFKLAVVLEGEG
YLQLQ
YLQLQPFSQPQ
YLQQ
YMQL
YNVPMATT
YPLGQGS
YPQQPQR
YPQRPQQ
YQQLQ
YRVAIMEV
YTVKEGDVIVAPAGS

1-0.5 kD

AAFKTSDERL
AAIHSVAH
AFLQPQHTFP
AFLQQCIQT
AGSIMHL
AHASSQVLQQSS
AHGRSQ
AIHSVVHAII
AIILH
AIILHQ
AIILHQQ
AIIMHQQ
AIIMHQQEQQ
AIIMQ
AIM
AIQSF
AIRNL
AIYSIPMTA
ALANQD

ALANQDVRVA
ALETLPAM
ALRPFD
ALRSM
AMPQRLARSQ
ANITPG
ANITPGSM
APAIYNIPMT
APFASI
APFASIV
APFASIVA
APFASIVAG
APFASIVAGIGGQ
APTAYNIPMVATY
APVGIFGTN
AQGLGIIQPQQPAQLEG
AQGTFLQP
AQGTFLQP
AQGTFLQPHQIAQ
AQGTFLQPHQIAQL
AQHESIRMSLQ
AQHKSMRML
AQIPEQIRCHAIHNVVEAIMQ
AQIPERIRCHAI
AQIPERT
AQIPQQLQCAAIHSV
AQLESIRMS
AQLEVMT
AQPQQL
AREVQEVF
ARSQMLQ
ASIVAGIGGQ
ASLVSSLW
ASPQQVSY
ASSQVLQQSTYQLLQQLCCQQL
ATFLQHQCSPVTVPF
ATTSVPFGVGTGVG
ATTSVRFGVGT
AVCSVA
AVTSIA
AYGSSQVLQ
AYGSSQVLQQ
CAAIHSIVHSI
CAAIHSVAHSI
CCQQLFQIPEQSRCQAIHNV
CERELQEHSLKACRQVVDQQLRDV
CHAIHIV
CIPGLERPW
CKNYLLQQCNPVSLVSSLVSM
CNIYIPVQCPAPTA
CQAIHNVVHAIILHHHQQQQQP
CQGMYQPQQPAKLES

CQQLAQIPQQLQC
CQQLQQI
CQVMQQQCCQQLAQIPQQL
CRTITQ
CTIAPFGIF
CVAFLQQKCGPL
DIFLPLSQH
DPQSGQGRQ
DPSGQVPWPQQQPFP
DPSGQVPWPQQQPFPQ
DPSGQVQW
DREL
DVVLQQH
EAIRQ
EASEQLQCDRELQELQERELKACQQVMDQQLRDISP
EEIRNL
EIRNL
EIRNLALQT
EITRMVLQ
ELQQPIPQQ
ELTFGRPA
ENLSLMCNIYV
EQIR
EQIRC
EQLR
EQQPRQLQ
EQQRYETVRA
EQQVVVP
ERNERV
ESIRMSLQ
ESTYQLV
ETSCIP
EVMTSIA
EVMTSIALR
FGQPQQQVPVE
FGVGTGV
FIQPQQPF
FIQPQQPFPQQP
FIQPSL
FIQPSLQQ
FIQPSLQQQV
FLQPHQIAQ
FLQPHQIAQL
FLQPQL
FLQPQLPYS
FLQPQQPF
FLQPQQPFPQQP
FLQQQCRPL
FLQQQPSF
FLQTQVEQ
FPPQQPYPHQQPFPSQQPYPQPQPF
FPQPQQPQQPFPQPQQPQLPFPQQPQ

FPQPQQPQQQF
FPQPQQPQQQFPQ
FPQPQQPQQS
FPQPQQPQQSFPQQQQ
FPQPQQTFPHQPQQQ
FPQQPLFSQ
FPQQQQ
FPQTQQPQQPFPQSKQPQQPFPQPQQ
FPSQQPYLQLQ
FPSQQPYMQLQPFPQ
FQLAQGL
FQQPLQ
FQQPLQQ
FRRIIQSDHG
FSQKQ
FSQKQQPV
FSQPQQPQQQ
FSQPQQQFPQPQQPQQ
FSQQPLFSQKQ
FSQQPQQAFLQ
FSQQQPILPQ
FSQQQPILPQQPPF
FVRALRP
FVVPGFTD
GCQLVR
GCQLVRQQC
GDRGRGDEAV
GGSFYPGETTPP
GHPVVEISSSQGSS
GIGVGVY
GKGR
GMYQPQQP
GMYQPQQP
GPLSQQQQVG
GQASSRRP
GQEQQPRQLQ
GQGQPGYYPTSSLQP
GQGSLVQGQG
GQGTLVQGQGI
GQKQPGY
GQPQQQVPVE
GQQLGQGQQGYYPTS
GQQQPFPPQ
GQQQQFPPQQPYPQPQPFPSQQP
GQVPLVQ
GQVQWLQ
GQVQWPQQQPF
GRGRGRH
GRGWH
GSAFVVPPGH
GSLVQGQGIIQ
GSPAQEL

GSVQPQQLP
GSVQPQQLPQ
GTLVQGQGI
GVGIGV
HAIIMHQQ
HAIIMQQ
HALANQDV
HARSQVLQQSTYQPLQ
HAVCGVA
HEQVGQG
HEQVGQGSLVQGQG
HGQ
HGSSQVLQQS
HLEAVTSI
HNIAHGR
HPSVLQ
HPVVEISSSQGSSNLQVVCFEIN
HQIAHL
HQIAHLEAVT
HQPQQQFP
HQQLL
HQQQRQQ
HRWPLPPFRGDSRDTFNLLEQRP
HSIIMQ
HSIIMQQQQ
HSIVHSIIMQ
HSVAHSI
HSVVEAI
HSVVHA
HSVVHAIIM
HSVVHAIIMQQ
HSVVHSIIMQQEQ
IAHA
IAHASSQVLQQ
IAPFGI
IAPFGIF
IAPFGIFG
IAPFGIFGTN
IAPFGIS
IAPVGIF
IAPVGIFGTN
IFWGIPA
IGKLGSPAQ
IHPSVL
IHSIV
IHSVVE
IHSVVEAIM
IILHQQ
IILQEQQQGF
IIMQ
IIMRQ
IIMRQQQG

IIMRQQQGQSFGQPQ
IIQPQQPAQL
ILGPLSQ
ILGPLSQQQQ
ILHQQ
ILPRND
ILQEQQQGF
ILQEQQQGFV
ILQQIL
ILQQQQ
ILSQQ
ILVQGQGI
IMGMVLQ
IMRAPFAS
IMRMVLQTLPSMCSVNIPQYCTTTPCSTITPAIY
IPEQSR
IPLVAT
IPPYCSTTIAPFG
IPPYCTIAPFG
IPPYCTIAPFGI
IPQPH
IPQPHQT
IPQQSR
IPVIHPSV
IPVQCPAPTTYNIPL
IPVVQ
IPVVQP
IQPSL
IQPSLQ
IQT
IQVDPSGQVQW
IRCHAIH
IRDYRV
ISPQQLGQGQQSG
ITPAIY
ITSAPLGVGSRVGA
IVASIGGQ
IVHSIIMQQEQQ
IVQPSVL
IVVEAIIQQQSQQQWQEPQQ
IYI
IYIPVQCPAPTAYNIPMVATYTGG
KACRQ
KELQSPQQSF
KNFLLQQ
KRSYTV
KTSDERLERLFNQ
KVAKAQQLAA
LAHGTF
LALQTL
LAQGLG
LAQGLGIIQPQQPAQLEG

LAQIPERT
LARSQ
LEQMKPCVA
LEQMRPCVAFL
LESIRMS
LETLPAM
LEVMTS
LEVMTSIALR
LFPELQQP
LGIIQPQQ
LGPLSQQQQ
LGQGSF
LGVGSRVGAY
LGYY
LHQQ
LIQPYL
LIQPYLQ
LLQQQI
LLQQSKP
LMQQQQ
LPFPQQ
LPFPQQSEQIIPQQLQ
LPPFRGDSRD
LPPQQSFSQQP
LPPQQTFP
LPPQQTFP
LPQFA
LPQFEEIRNLALETLPAMCNVYIPPYCTIAPVG
LPQIPQQSR
LPYSQPQPFRPQQPYPQPQPQYSQPQQ
LQELQERE
LQEQLTPC
LQHQCSPVTVP
LQLQPFPQ
LQLQPFSQPQLPYSQPQPFRPQQPYPQ
LQNPSQQQPQEQVPLVQEQ
LQPEQGQQG
LQPFL
LQPFL
LQPFPQPQPFL
LQPGQW
LQPHQI
LQPHQIAQ
LQPRQPFPQQPQQPY
LQQCIQTPYVQSQ
LQQHNIAQGR
LQQHSIAHGSSQVLQQSTY
LQQLCCQQLL
LQQLVQGQQGQQPERGQQGQQSGQGQQLGQGQQGQQP
LQQPIPQQPQ
LQQPTQGQQRQQPGQGQQLR
LQQQCI

LQQQIPIVH
LQQSTYQLLQQLCCQQ
LQQYPL
LQQYPLGQG
LQT
LQVVCF
LRDVGPECQPVGGGPVARQ
LRQGQQ
LRSMCSIYIPVQC
LRTL
LRTLPNM
LSLM
LSQIP
LSQIPEQYRCQAIHN
LSQSQQQSQQQLGQC
LTSPQQLGQGQQPRQW
LVATYT
LVPQLQQ
LVQGQGIIQPQQPAQL
LVQGQGIIQPQQPAQY
LVSMILPRND
LWSMILP
LWSMILPR
LYL
LYRTTTSVPFGVG
MLLETLYL
MRMLLETLYLMCNIYVP
MRQQCCQQPAQIP
MRQQQG
MSLQA
MVLQT
NAPFASIVA
NAPFASIVAG
NIAHARSQVLQ
NIAHASSQ
NIAHGRSQ
NIAQGRS
NIAQGRSQ
NIQVDPSGQVQW
NIVHGRSQVL
NIYVPV
NIYVPVQ
NIYVPVQCQQQQQLG
NLALETL
NMQVDPSGQVQWPQQQ
NPCKDFLLQQ
NVAHA
NVPLYS
NVYIPPHCS
PAIQSF
PAMCNVYIPPYCSTTIAP
PASLVSS

PASLVSSL
PCRTITQTPYN
PEQIRCHA
PFASIVAGIGG
PFDQVSRL
PFGVGTGVGA
PFIQPSLQ
PFIQPSLQQQ
PFLPQL
PFLQTQVE
PFPPQLPY
PFPQQPYPQQPYPSQQPYPSQQPFPT
PFPQQSEQIIPQ
PFPSQLPYL
PFPSQQPYMQLQPFPQPQLPYP
PFTQQQQQQQQ
PGLERPWQ
PGQEQQGQQP
PGQEQQGQQPGQGQQP
PGQLQQLVQGQQGQQPERGQQGQ
PGQQEQFPPQQPYPHQQP
PGQWLQSGYYLTSPQQ
PHQIARLE
PILSQQ
PKYLQPQQP
PLFQL
PLPPFRGDS
PLSQQQQVGQ
PLVQQQQFLGQQQP
PLVQQQQFLGQQQPFPPQQPYPQLQ
PLVQQQQFLGQQQPFPPQQPYPQPQPF
PLYQQ
PLYQQQQV
PLYRTTTSVPFGVG
PPFAQQQQPPFSQQPPI
PPFSQQPPISQ
PPFWQQQPPF
PPFWQQQPPF
PPKGGTFYP
PQAQGSFQPQQ
PQAQGSVQP
PQAQGSVQPQQLPQF
PQIPQQSRYEAIRA
PQLPYPQPPPF
PQLQLQNPSQQQ
PQLQQPQQ
PQNPSQQQPQK
PQPFPS
PQPHQPFSQQPQQ
PQPQPFSQQPQQAFLQPQHT
PQPQPQPQPQP
PQPQQPQL

PQQLQQSILWG
PQQPAFSQQQQT
PQQPAQLE
PQQPAQLES
PQQPFPQLQQPQQPFPQPQQQLPQPQQPQ
PQQPFPQQPQQP
PQQPFPQTQQPQQPFPQQPQQPFPQT
PQQPQQPFPLQPQQPFPQQPQQPFPQ
PQQPQQQFIQ
PQQPQQQFLQ
PQQPYPQPQPFPSQQP
PQQPYPQPQPFPSQQP
PQQQA
PQQQFPQPQQ
PQQQPFPQPQQP
PQQQPFPSQQ
PQQQPPFIQPSLQQ
PQQQPSLIQ
PQQQPYGSSLT
PQQQQPAIQSFLQ
PQQQRPFI
PQQSFP
PQQSFPQQQR
PQQTFPQQPQLP
PQQTYPQRPQQ
PQSFPPQ
PQSGQGR
PQSQPQYSQPQQPISQQ
PQSQQPQ
PQSQQPQQQF
PQTQPFPP
PQTQQPQQL
PRQPFPQQPQQPYPQQPQQPFPQTQQPQQPF
PRYS
PSGQVQW
PSGQVQWPQ
PSLIQQ
PSLQQQLNP
PSQLNP
PSQQPF
PSQQQPQ
PSVLQQL
PTMCSVNVPLYRTTTSVPFG
PTMCSVNVPLYSATTSVPF
PTQGQQRQQPGQGQQLRQGQQGQQSGQG
PVEIMGMVLQ
PVQCPAPTA
PVVVSPVAGQYEQQ
PWPQQQ
PWQPQQ
PYCSTIRAPFASIVASIGG
PYCTI

PYLQLQPFS
PYPQPQPFPSQQP
QALRS
QARCQAVCSVAQIIMRQQQG
QASSCQLM
QASSRRPGQGQQEYYLT
QCAAIHTIIHS
QCCQQLAQIPQQ
QCKPVSLVSSLWS
QEHSLKACRQVV
QELCCQHLWQIPEQSQCQAIHNVVHAI
QEQLTPCATFLQHQCSPVT
QEQQPRQLQQPEH
QEQQQGVP
QEQQQVQGSIQSQQQQP
QFIQPQQ
QFLGQQQ
QFLQPQQ
QFPQPQQPQQSFPQQQPP
QFQEIRNLA
QFSFQQPL
QGFGQSQQQQQPGQRQLLEQMKPCVAFLQQKCS
QGFRQYQQQQQPGQR
QGIDIF
QGQQPGQR
QGQQRQQPGQ
QGQSFGQPQQQVPVEIMRMVL
QGQSIIQ
QGSFQPSQQNPQAQGSVQPQ
QGSVQPQQLPQ
QGTFLQP
QHQQLLQQQ
QIAQLE
QIARLEVM
QIIPQQLQQP
QILQQI
QILQQIL
QIPEQ
QIPERTRCH
QIPRQLQC
QIPVVQ
QIPVVQP
QIVCPHLGR
QLAQGTFL
QLGQGQPRYYP
QLGQGQQGQQPGQ
QLGQQPQQQQLAQGTF
QLGQQQQQQL
QLLQCSF
QLLRELCCQHLWQIP
QLPFPQQSEQIIPQQ
QLPQE

QLPQEQVPLVQ
QLPQFEIRN
QLPYLQ
QLPYPQTQ
QLPYPQTQPF
QLQCTAI
QLQPKN
QLQQLC
QLQQPAQ
QLQQPFPLQPQ
QLQQPTQGQQRQQPGQGQQLRQGQQGQQSGQ
QLRDVGPECQPV
QLSQIPEQFRC
QLSSCQIVQHQCCQQLAQIPERIRCHAIHS
QLT
QLTPCTTFL
QNPQAQG
QNPQAQGSVQPQQL
QPAQLE
QPFLPQLPYPQPQSFP
QPFLQPHQPFSQQPQQIFPQP
QPFPQPQLPYSQPQPFRPQQ
QPFPQPQQPFP
QPFPQPQQQLPQPQQPQQSFP
QPFPQPQQTF
QPFPQQPQQS
QPFPQTQQPQQP
QPFRPQQPY
QPFRPQQPY
QPGQRQLLE
QPGQRQP
QPILPQ
QPLPPQQTFPQ
QPLQQLCCQQL
QPPFSQQPP
QPPFSQQQPILS
QPQGQVP
QPQHTFPL
QPQPFPSQQPYLQLQPFPQPQPFP
QPQQA
QPQQIFPQPQQTFPHQPQQQFPQPQQP
QPQQLGQCV
QPQQLPQFQ
QPQQPAK
QPQQPFPQSQ
QPQQPFPQT
QPQQPR
QPQQPRQ
QPQQPYPQQPQQPFPQTQQPQQPFPQSKQ
QPQQQFP
QPQQQQLAH
QPQQQQQQVLQGTFLQPH

QPQQQYPSGQGFFQP
QPQQSFPQQQ
QPQQSGQGVS
QPQQVFPQPQQPQQQFPQPQQP
QPQSFP
QPSSQVSF
QPVLPPQQSPFPQQQQQH
QPYLQ
QPYLQLQPFSQ
QPYPHQQPFPSQQPYPQPQPFPPQLPYPQTQP
QPYPQPQPFPSQQPYLQLQPF
QPYPQQQPFPSQQPYMQLQP
QPYPQQR
QQCKPV
QQCSPVAMPQHL
QQEQLQGVQILGPLSQQ
QQEQQQGVPI
QQFIQPQQP
QQFPGQQQPFPP
QQGFVQPQ
QQGQQQRQ
QQHNIAHGSSQVLQE
QQIGQGQQPGQLQQPTQGQQGQQ
QQILQQ
QQILQQQLT
QQIVV
QQLAQIP
QQLCCQQLFQIPEQSRCQAIHNVV
QQLGQGQPRYYPT
QQLGQQPQQQQVQKGTFLQPHQI
QQLIPC
QQLLQQ
QQLPQFEEIRNLALQTLPAMCNV
QQLQCAAIHSV
QQLQCAAIHSVVHSIIMQQEQQEQLQ
QQLQQLC
QQLVLPQ
QQLWQ
QQPAQ
QQPAQLEAIRSL
QQPAQLES
QQPFLQ
QQPFLQPH
QQPFLQPHQ
QQPFPQPQLPFPQQSEQIIPQQLQ
QQPFPQPQQPQL
QQPFPQPQQQF
QQPFPQPQQQFPQ
QQPFPQQPPFLQ
QQPFPQQPQQ
QQPFPQQPQQPYPQQPQQPFPQTQQ
QQPFPQQPQQTYPQRPQQ

QQPFPQSK
QQPFPQT
QQPFPS
QQPGQLQQSTQ
QQPILSQQP
QQPPFA
QQPPFS
QQPPFSQQQQPV
QQPQQIFPQ
QQPQQLG
QQPQQLGQCVS
QQPQQPFPQPQL
QQPQQPFPQPQQQFPQPQQPQQSFPQQQPSLI
QQPQQPYPQQQPYGSSLTSIG
QQPQQQQQQVLQGTFLQ
QQPQQQQQVLQGTFLQPH
QQPQQSFPQQQPSLIQQSLQQQL
QQPQQSGQGLSQS
QQPQQSVQGVYQP
QQPSSQVSFQQPLQQYPL
QQPTPI
QQPVLPQQS
QQPYLQL
QQPYLQLQPFS
QQPYPH
QQQGFVQ
QQQGIDIFL
QQQILQ
QQQLFPQ
QQQLFPQQP
QQQLIPCR
QQQPFLQ
QQQPFPQ
QQQPILPQQ
QQQPILSQQ
QQQPILSQQPPFSQQQQPVLPQQSPFS
QQQPLPPQQSFSQQP
QQQPLPQQ
QQQPQYLQPQQPI
QQQPSLIQQ
QQQQFLGQQQPFPPQQPY
QQQQLF
QQQQLGQQQQQQLQEQLTPCTT
QQQQPLPQQ
QQQQPP
QQQQPVLPQ
QQQQQLGQQQQQQLQEQLTPC
QQQQQPLSQVCF
QQQQQQLFPQ
QQQQQQQPSSQVSFQQPQ
QQQQQQQQQQPFTQQQQ
QQQQVLQG

QQQSQQQWQERQQQAQHKSMRMLLENLSLM
QQQVPVEIMRMVLQTLPSMCSV
QQRQERQQQAQH
QQSGQGQQPGQR
QQVPVEIMGM
QRCLARS
QSEQIIPQQL
QSFPQQQR
QSGQLQQPA
QSIIQYQQQQ
QSILWGV
QSRCQAIHNVVHAIILHHHQQQQQQPSS
QSTYQLLQQLCCQQLLQIPEQSQCQAI
QSTYQLVQQLC
QTFPHQPQQQFP
QTFPQQPL
QTPH
QVDPSGQVQ
QVEQLSSCQIDQ
QVGQGIL
QVLQESTYQL
QVLQESTYQLVQ
QVLQGTFLQPH
QVPLVQ
QVPLVQQQQFLGQQ
QVQGSIQSQ
QVQKGTFLQP
QVSFQQPLQ
QWPQQQPFLQ
QWPQQQPFPQPQ
RAAF
RDVVLQQHS
REHGKGRRREEEEDDQRQQRRRGSESESEEEEEQQRYET
RFPVP
RGRGRG
RGRGRGRH
RHRRRGD
RLEVMTSIAL
RLFNQRQGQEKTRS
RMPFLQ
RMVL
RNERVW
RPFIQ
RPMYL
RQGQQSGQR
RQPFPQQPQQPYPQQPQQPFPQTQQPQQ
RQQCCQQLAQIPQQLQCAAIHSVVHST
RQQQAQHKSM
RSLVLKTLPTM
RSMCNIYIPVQCPAPTAYN
RSQVL
RSRDITS

RVDQPTSCQN
RVWLAGR
RYYPTCP
SFLQQQMNPC
SFPLQPQQ
SFQQPLQQYPL
SFYPGETTPPQQLQQSILWGVP
SGQGQLGY
SGQGQLGYYPTSPQQ
SGQKQP
SGQVPWPQQ
SIAHGSSQ
SIAPRTL
SIAYGSSQVLQQSTYQL
SIIMQ
SIIMQQ
SIIMQQE
SILQQLNPCKVF
SIRMSLQALRS
SIRMSLQALRSMCRIYIPVQCPAPTAY
SIVAGIGGQ
SIVRAS
SLIQQ
SLQQQL
SLSKRVLRA
SLVQGQGI
SLVQGQGIIQPQ
SLVSSL
SLWSI
SMRMLLENLSL
SMRMLLETLYLM
SPFPQQ
SPQQLG
SPQRSR
SPVTVPFPQ
SQKQQPV
SQPQQPQQQF
SQPQQQVPVEIMRMVL
SQPRQGQ
SQQPYLQLQPF
SQQPYLQLQPF
SQQQLL
SQQQPILPQQP
SQQQPILPQQP
SQQQQQPGQRQL
SQQQQQPGQRQL
SQQQQQQQQQQQQQQEQQILQQILQQQLI
SQQQR
SRYEAI
SSQVQWPQQQ
SSQVQWPQQQPV
STIRAPFASIVAG

SVAHSIIMQ
SVLQQLN
SVNIPQY
SVNIPQYCTTTPCSTITPAIYSIP
SVNVPLYETTTS
SVNVPLYSA
SVNVPLYSATTSVRF
SVPFGVGTG
SVVHSTIMQ
TAIHSVVHA
TCSALLQQCSPTPYVQSQM
TFHHQPQQ
TFLQHQCSP
TIAPAIY
TIAPFGIFG
TIMQQEQQ
TINAPFASIV
TIPQPHQ
TLPAMCNVYIP
TLPLMCR
TPCRTITQTP
TPCSTIAPAI
TQGQQRQQPGQ
TQPQQPT
TQQQQPPFSQQPP
TQQSFPL
TRSVSIVRASEEQ
TSDERLERL
TTIAPFGIF
TTIAPFGIFGTN
TTPCSTIAPAI
TTSVPFGVGTGVGA
TTTSVPFGV
TTTSVPFGVG
TTTSVPFGVGTGVG
TTTSVPFGVGTGVGA
TTTSVPFGVGTGVGAY
TTTSVPL
TYNIPL
TYQLV
VAEAIRHQQ
VAFLQQQC
VAKAQQL
VAMPQHLAR
VAMQRCLARSQML
VAQIIM
VDPSSQVQWPQQ
VEIMGMVL
VEQLSSCQIVQH
VETFLRMAT
VHGRS
VHGRSQ

VHSIIMQQQQQQ
VHSTI
VLPPQQQ
VLQQHSIAH
VLQQSSY
VLQQSSYQQLQQ
VLQQST
VLQQST
VLQQSTYQ
VLQQSTYQL
VLQQSTYQPL
VLQTLPLM
VLQTLPSMCNV
VLQTLPSMCNV
VMTSIA
VNPRAF
VNVPLY
VNVPLYR
VPLSQQQQVGQGT
VPLVQQQQFLGQQQPFPPQQPYPQLQP
VPLYET
VPLYETTTSV
VPLYSATTSVPFGVGTGVGA
VPQLQLQ
VPVPQL
VPVPQLQPQNPSQQQPQEQ
VQGQGII
VQGQGIIQPQQPAQL
VQGQGIIQPQQPAQY
VQPQQSFPQQSQQ
VQQLCCQQLWQI
VRAL
VRPDC
VRVSVPQ
VSFQQPQQQYPSSQG
VSLVSS
VSLVSSLVSM
VSPVAGQY
VSQIIMR
VSRGSAFVVPP
VSSLW
VSYYPGQASPQR
VVHAIILHQQQQKQQQQPSSQFS
VVHAIILHQQQQKQQQQPSSQFSFQQ
VVHAIIMHQ
VVHAIIMHQQ
VVHAIIMHQQ
VVLQQH
VVPPGHPVVE
WLAGRN
WLQPGQW
WLQPGQWLQSG

WPQQQPFL
WQIPEQSR
WQQQPLQQKETFPQQPPSSQQQQPFP
WQQQPP
WSIIWPQSD
YCTIAPFGIF
YETTTSVPLGVG
YIPPHCSTTI
YIPVQCPAPTTY
YLNTQSFKLAVVLEGEG
YLTSPQQSGQWQQPGQGQ
YPLG
YPLGQGS
YPQLQ
YPQPQPQYSQPQQPISQ
YPQQPQR
YPQRPQQ
YQLLRE
YQQQQPQQL
YRVAIMEV
YSIILQQQQQ
YSQQQQPPFS
YSTSPQQL
YTVKEGDVIVAPAGS
YVAQG
YVAQGEGVLTV
YYPTSSQQPGQL

3-1 kD

AAFLQQCIQT
AAFLQQCS
AAIHSIVHS
AAIHSVAHSIIMQ
ACQQVMDQQLRDISPECHPVVVSPVAGQYEQQIVV
ADPSGQVQWP
AFLQQCIQTPY
AFSQQQQTVL
AGRNNVIGKLGSPA
AGSIMHLANT
AGSIMHLANTDGR
AHARSQ
AHARSQV
AHGSSQVLQESTYQLVQQLCCQQLWQ
AIHSVAHSIIMQ
AIHSVVHAI
AIILHQ
AIILHQQ
AIILHQQQQQ
AIIMHQQEQQ
AIIMHQQEQQQ
AIIMHQQQQ

AIIMHQQQQQQQEQ
AIIMQQ
AIIYSIIL
AIM
AIYNIPMTA
ALETLPAM
ALLQQCSPTPY
ALQTL
ALQTLPAM
ALQTLPAMCNVYIPPYCT
AMCNVYIPPHCSTTIAPFGI
AMPQHLARSQMW
AMPQRLARSQ
AMQRCLARSQMLQQSICHV
ANRHGRLYEADA
APFASIV
APFASIVA
APFASIVAG
APFASIVAGIGGQ
APTAYNIPMVAT
APTAYNIPMVATY
APTTYNIPLVATYTGG
APVGIF
APVGIFGTN
AQGLGIIQPQQPAQ
AQGLGIIQPQQPAQLEG
AQGSVQPQQLPQFEI
AQGTFLQPHQIAQ
AQGTFLQPHQIAQL
AQGTFLQPHQIAQLEVMTS
AQIIMRQQ
AQIPEQIRCHAIH
AQIPERT
AQIPQQLQ
AQPQQL
ARELNPSNK
ARELNPSNKE
ARSFHALANQ
ARSQMLQ
ASIVAGIGGQ
ASLVSSLW
ATTSVPFGVGTGVG
AVANITPGSMT
AVTSIA
AYGSSQVLQ
CAAIHSIVHSI
CIQTPYVQSQ
CKNYLLQQ
CKPVSLVSSLWSMIL
CPAPTAYNIPMV
CQAIHNVAHAIIMHQQ
CQPLAQISEQA

CRIYIPVQC
CRLEGGDALLAS
CSALLQQCSPTPYVQSQ
CSPVAMPQRL
CSPVAMPQRL
CSVNVPL
CTIAPFGIF
DARSFHAL
DDRRGG
DEAVETFL
DPSGQVQW
DRRGGH
DVIVAPAGSIM
DVIVAPAGSIMH
DVVLQQH
EAIIQQQS
EAIIQQQSQQQW
EASEQLQCDRELQELQERELKACQQVMDQQLRDISP
EEIRNL
EGEVQIVCP
EHGRGRGR
EHGRGRGRHGEGE
EHSLKACR
EIMRMVLQT
EIRNL
EIRNLALQT
EITRMVLQ
EKLQCQAIHN
ELCCQHLWQIPEQSQCQAIHN
EQERHRRRGDR
EQIIPQ
EQIRC
EQKQQLQQQQ
EQQEQLQGVQILGPLSQQ
EQQPRQLQ
EQQRYETVRA
EQSQCQAIHNVVHAII
ERGRGH
ERPWQQQPL
ERQQQAQHKSMRMLLENL
ESIRMSLQA
ETTTSVPLGVG
EVMTS
EVMTSIA
EVMTSIALR
EVQEVF
FAQPQQL
FASIVASIGGQE
FGQPQQQVPVE
FHALANQDV
FIQPQQPF
FIQPSL

FIQPSLQQ
FIQPSLQQQV
FLPQ
FLPQL
FLQHQCSPVTVP
FLQPHQIAQ
FLQPHQIAQL
FLQPHQIAR
FLQPHQP
FLQPQQPF
FLQQCSPVAMP
FLQQKCSP
FPELQQP
FPPQ
FPPQLPYPQTQPF
FPQIPVDQPTSCQNVQHQCCRQ
FPQP
FPQPQPFP
FPQPQQPFPW
FPQPQQPQQQF
FPQPQQPQQS
FPQPQQPQQSFPQQQQ
FPQPQQT
FPQPQQTFPHQPQQQ
FPQQPL
FPQQPQQPFPQTQQPQQPF
FPQQPQQPYPQQQPYGSSL
FPQQPSFSQQQPPFWQ
FPQQQQQHQQLVQQQIP
FPQQQRPF
FPQSKQ
FPQSKQPQQPFPQP
FPQSQQPQQQ
FPQTQQPQQPFPQSKQPQQPFPQPQQ
FPSQLPYL
FPSQQPYMQLQPFPQ
FPSQQPYPQPQPF
FPVPQL
FQLVQGQG
FQPSQQNPQAQGSFQ
FQQPLQ
FQQPLQQ
FSFQQPLQQYPLGQGSFRP
FSQPQLPYSQPQPFRPQQPYPQPQ
FSQPQQQFPQPQQPQQ
FSQQPQQAFLQ
FSQQPQQT
FSQQPQQTFPQPQ
FTQPQQPT
FVVPPGHPVVEIS
GDALLASQ
GDRGRGDEAV

GEGEREEEEGRGRGRRGEGER
GEGEVQIVCPHLG
GEGQQGQQP
GFVQPQQ
GHPV
GIDIFL
GIIQPQQPAQYEVI
GLSQSQQQSQQQ
GPLSQQQQVG
GQASPQRSRDITSS
GQEQQPRQLQ
GQGIIQPQQPAQLE
GQGQPGYYPTSPQQIGQGQQP
GQGQPGYYPTSPQQSGQLQQP
GQGQPGYYPTSSLQP
GQGQQPGQLQQPT
GQGQQPGQLQQPTQGQQGQQP
GQGTLVQGQGIIQ
GQKQPGYYP
GQLQQPAQGQQPG
GQLQQSTQEQQLGQEQQDP
GQPQQQVPVE
GQQGQQPERGQQGQQSGQGQQLGQGQQ
GQQLGQGQQGYYPTS
GQQPRQWS
GQQQPFPPQ
GQQQQFPPQQPYPQPQPFPSQQP
GQRFGQPQQQQ
GQRQLLEQM
GQVQWLQ
GRGWH
GSPAQEL
GTFLQPHQIAR
GTFYPGETTPPQQL
GVAQVI
GYVAQGEGVLT
GYYLTSPQQLGQG
HAIILHHHQ
HALANQDV
HGRLYE
HGRSQVLQQSTY
HHQPQQTFPQPQ
HHQPQQTFPQPQQTYPHQPQQQFPQTQQPQQPFP
HILL
HKSMRML
HLANTDGR
HLWQIPEQ
HNIAHASSQVLQQSTY
HNIAHGRS
HNVAHA
HPVVEISSSQGSSNLQVVCFEIN
HQPFSQQPQQTF

HQVPIEIT
HRRRG
HSIIMQ
HSVAHSI
HTFPLQPQQ
HTISVPGKF
HVPPYCSTIR
HVSVEH
IAHGSSQVL
IAPFGIF
IAPFGIFGTN
IAPFGIS
IAPVGIF
IAPVGIFGTN
IAQLEVMTSIA
IAYGSSQVLQ
IAYGSSQVLQQ
IFWGIPA
IGQGQQPGQL
IGSLVLQTLPTMCNVHVPPYCST
IHNVVEA
IHSIIMQ
IHSIV
IHSVAHS
IHSVV
IHSVVHS
IHTIIHSIIMQ
IILHHHQQQQQ
IILHQQ
IILPR
IILQQQ
IIMHQQEQQQQLQ
IIMQ
IIMQQEQ
IIMQQEQQEQL
IIMQQEQQGI
IIMQQEQQGIQIL
IIMQQQQQQQ
IIMRQ
IIMRQQQG
IIQPQQPAQL
IIQQQ
ILGPLSQ
ILQEQQQGFV
ILQQIL
ILQQQ
ILQQQL
ILQQQQ
ILQQQQQQQ
ILSQQPPFSQ
ILVQGQGI
ILWGVPA

IMGMV
IMGMVLQ
IMGMVLQTLPSMCS
IMQQEQQEQLQ
IMQQQSQQQR
IMRAPFAS
IMRQQQGQRF
IMRQQQGQRFG
INAPFASIVAGIG
IPEQFRCQAIHN
IPEQSR
IPIVH
IPLVA
IPLVAT
IPLVATYT
IPMTA
IPMVATYTG
IPPHCSTTIAPFGIS
IPPYCSTTIAPFG
IPPYCTIAPFG
IPPYCTIAPFGI
IPQQLQCAAIHSIVHS
IPQQLQCAAIHSV
IPQQSR
IPQQSRYEA
IPVIH
IPVIHPSV
IPVVQ
IPVVQP
IQPQQPA
IQPQQPFPQQPQ
IQPSL
IQPSLQ
IQPSLQQQ
IQPYLQQ
IQVDPSGQVQW
IQVDPSGQVQWPQQ
IQYQQQQPQQL
IRAPFASIV
IRCHAIH
IRHQQPQQQCQGMYQPQQPAK
IRMSLQALRS
IRSL
IRVDQ
IRVDQPTSC
ISPECHPVVV
ISQQQAQQ
ISQQQQQQQQQ
ITPAIY
ITPAIYSI
ITSAPLGVGSRVG
ITSAPLGVGSRVGA

IVAGIGG
IVAGIGGQ
IVAGIGGQ
IVASIGGQ
IVQPSVL
IVVEAIIQQQSQQQWQEPQQ
IYNIPMTATCAGGA
IYVPVQCQQ
KACRQVVDQQLRDVGPECQPVGGGPVAR
KEGDVIVAPA
KELQSPQQSF
KGGSFYPG
KNYLLQQCN
KQQSGQG
KSQVLQQSTYQLLQ
LALETLPAM
LALQTL
LAQGLGIIQPQQPAQL
LAQGLGIIQPQQPAQLEG
LAQGTFLQP
LAQIPERT
LAQISEQARCQAVC
LARSQMLQQSIC
LARSQMW
LARSQMWQQS
LARSQMWQQS
LEAVTSIA
LEGGDAL
LENLSLMCNIYVPIQC
LEQMKPCVAFLQQKCS
LEQMRPCVAFL
LESIRMS
LETLPAM
LEVMTS
LEVMTSI
LFPQ
LFQIPEQS
LFQLVQGQG
LGQGQPRYY
LGQQPQQQQLAH
LGSPAQEL
LHQQ
LHQQ
LLEQMKPCAAFL
LLETLYLMCNIYVPIQCQQQQ
LLQIPEQSQCQAIHNVAHAI
LLQQQ
LLQQQI
LLQQQIPIVHPSI
LLRELCCQHLWQI
LMCRVNI
LNPQAQGSVQPQQLPQF

LPPQQSFSQQP
LPQEQVPLVQQQ
LPQFEEIRNLALQTLPAMCNVYIPPYCT
LPQQPAFSQQQQTV
LPYPQPQLPYPQPQ
LPYPQPQSFPPQQ
LQCSFQQPQQQLG
LQCTAIH
LQELQERE
LQEQLTPCA
LQEQLTPCTTFL
LQEQQQVQ
LQHQ
LQIPEQ
LQIPEQSR
LQLQPFPQ
LQNPSQQQPQEQVPLVQEQ
LQPFL
LQPHQIAR
LQPQQPFPQQPQ
LQPRQPFP
LQQCIQTPYVQSQ
LQQCIQTPYVQSQMW
LQQHNIAQGR
LQQHNIARG
LQQHNIVHGK
LQQHSIAYG
LQQHSIAYGS
LQQILQ
LQQKETFPQ
LQQLNPCKVFLQQQC
LQQLNPCKVFLQQQCSPVAMPQR
LQQQCSPVTV
LQQQIPI
LQQQIPIVH
LQQQQQQQL
LQQSTYQLLQQLCCQQ
LQQYPLGQG
LQTQVEQ
LQVVCF
LQVVCFEI
LRIL
LRILP
LRPFD
LRQGQQGQQSGQG
LRQGQQGQQSGQGQP
LRSMCSIYIPVQC
LRTLPNM
LRTLPTM
LSLMCNIYVPVQCQQQQQLGQQQ
LSQIP
LSQIPEQFR

LTSPQQLGQGQQPRQW
LVATYTGG
LVPQLQQ
LVQGQGI
LVQGQGIIQPQQPAQL
LVQGQGIIQPQQPAQY
LVQQQIPIVQPSV
LVSMILPRND
LYRTTTSVPFGVG
MCSIYIPVQCPAPTA
MEVNPRAF
MLLENLSLMCNIYVPIQCQ
MNPCKNYLL
MPFLQ
MRMLLETLYLMCNIYVP
MRMVLQTLPSM
MRQQCCQQPAQIP
MRSIYIPVQCP
MTAPYLN
NIAHARSQVLQ
NIAHASSQ
NIAHGRSQ
NIAHGRSQV
NIAQGRSQ
NIQVDPSGQVQW
NIYVPV
NIYVPVQ
NIYVPVQCQQQQQLG
NKELQSPQQ
NKELQSPQQS
NLALETL
NLSLMCNIYVPVQCQQ
NMQVDPSGQVQWPQQQ
NPQAQGSVQPQQLPQFEEIRN
NRHGRLY
NVPLYR
NVPLYRTTTSVPFG
NVPLYRTTTSVPFG
NVVEA
NVYVPPYCSTIRAPFASIVASIG
PAIYSIPMTATCA
PAPTAYNIPMVAT
PAREVQE
PASLVSS
PASLVSSL
PASLVSSLW
PCKNYLLQQCNPV
PCSTIAPAI
PCVAFLQQKCGPLRMPF
PEQIRC
PEQLQQPTQGQ
PEQSRYEAIR

PERIRCHAIHSV
PFAQQQQPP
PFASIVAGIGG
PFDQVSRL
PFGVGTGVG
PFGVGTGVGA
PFIQPSL
PFIQPSLQ
PFIQPSLQQQ
PFLPQL
PFLQ
PFLQPHQPFSQQ
PFPPQLP
PFPPQLPYPQPQ
PFPQ
PFPQPQLPYSQPQ
PFPQQPPF
PFPQQPPFLQQQ
PFPQQSEQIIPQ
PFPQTQQSFPLQPQQPFP
PFPSQLPYL
PFPSQQPYMQLQPFPQPQLPYP
PFPTPQQQFPEQS
PFSQQQPIL
PFSQQQPILPQQPPF
PFTQQQQQQQQ
PGETTPPQQL
PGQASSRRPGQG
PGQLQQLVQGQQGQQPERGQQGQ
PGQQEQFPPQQPYPHQQP
PGQWLQPGQWLQSGYYLTSPQQLGQGQQPRQW
PGQWLQSGYYLTSPQQ
PGSMTAPYLNTQSF
PHQIAQLE
PHQPF
PHQPQQQ
PILSQQ
PIPQQPQQPFP
PIPQQPQQPFPLQPQQPFPQQPQQPFPQQPQQS
PIQPQQPFPQQPQQPQQPFPQPQQPF
PISPQQLGQGQQSGQGQ
PISQQ
PKGGSFYPGETTPPQQ
PLAQISEQAR
PLFQL
PLMQQ
PLPPFRGDS
PLQPGQGQPG
PLQQYPLGQGS
PLSQQ
PLSQQPQQT
PLVQQQQFLGQQQP

PLVQQQQFLGQQQPFPPQQPYPQL
PLVQQQQFPGQQQQ
PLYQQQQV
PLYQQQQVGQG
PLYRTTTSVPFGVGTGVGAY
PPFWQQQPPF
PPGHPVVE
PPPFSPQQPYPQPQPQY
PPQLPYPQTQ
PPQQPYPQP
PQAQGSFQPQQ
PQAQGSVQP
PQAQGSVQPQQL
PQAQGSVQPQQLPQF
PQFSQQPPFSQQQQPPFSQQQQQPPFA
PQHLARSQM
PQIRVDQPTSCQ
PQLPYSQPQ
PQLQQPLSQQP
PQPFPSQ
PQPHQTFHHQP
PQPPPFSPQQPYPQPQP
PQPQLPYSQ
PQPQNPSQPQPQG
PQPQPFR
PQPQPQNPSQPQPQ
PQPQQPIS
PQPQQPQL
PQPQQT
PQQAFLQ
PQQLGQC
PQQLQCAAIHSIV
PQQPAKLE
PQQPAQL
PQQPAQLE
PQQPAQLEGIRS
PQQPAQLESIRM
PQQPFPQPQQPFPWQPQQ
PQQPFPQQPQQP
PQQPGQEQQPRQLQQPEHGQQGQQPEQGQQGQQQRQG
PQQPISQQQAQQQQ
PQQPQQQFLQPR
PQQPQQSFPQQQPSL
PQQPQQSFPQQQR
PQQPYPQPQPFPSQQPYLQLQPFPQPQLP
PQQPYPQQQPFPSQQPYMQL
PQQQFPQPQQ
PQQQPFPQPQQP
PQQQPFPSQQ
PQQQPYGSSLTSIGG
PQQQQLA
PQQQQLAQGTF

PQQQQPAIQSFLQ
PQQQQPAIQSFLQQQMN
PQQQSQQQLLQCSFQQPQQQLG
PQQRPMYLQPQQ
PQQTFPH
PQQTFPHQPQQQFPQPQQPRQ
PQQVFP
PQQVSYYPGQASPQRSR
PQTQQPQQL
PRQLQCTA
PSFSQQPLFSQ
PSGQVPWPQQQPFPQPH
PSGQVQW
PSGQVQWL
PSGQVQWPQ
PSGQVQWPQQQPFPQPQQPFCQQP
PSLIQQ
PSLQQ
PSLQQQLNP
PSQLPYL
PSQQNPQAQGSFQP
PSQQQPQKQVPLVQQQ
PSSQVSFQPSQLN
PSSQVSFQQ
PSSQVSFQQPLQ
PTMCSVNVPLYRTTTSVPFG
PTSPWQPEQLQQP
PTSSQQPGQLQQ
PVAMPQR
PVAMQRCLAR
PVLPPQQSP
PVLPQQPAFSQQQQ
PVVEISSS
PVVVSPVAGQY
PVVVSPVAGQYEQQ
PWQQQPLPPQQSFSQQPPFSQ
PWQQQPLPPQQSFSQQPPFSQQQQQP
PWQQQPLPPQQT
PWQQQPLQQKE
PWQQQPLQQKETFPQQPPSSQQQQPF
PYCTI
PYLN
PYNVPMAT
PYPQPQPFP
PYPQPQPFRP
PYPQQPQQPF
PYPQQQPQYLQP
PYVQ
PYVQSQMWQASGCQLMRQ
QAFLQPQ
QARCQAVCSVAQIIMRQQQG
QCAAIHTIIHS

QCCQQLAQIPQQ
QCIQTPYV
QCNHVSLVS
QCPAPTAYNIPMV
QCSFQQPQQQLGQQPQQQ
QEHSLKACRQVV
QEQERHRRRGDRGRGD
QEQLTPCATFLQHQCSPVT
QEQQPRQLQQPEH
QEQVPLMQQQQ
QERELKACQQVMDQQLR
QFAEIRN
QFEIRNLAL
QFIQPQQ
QFLGQQQP
QFLQPQQ
QFPGQQQQFPP
QFSQPQQPQQ
QGHRWPLPPFRGDSRDT
QGIDIF
QGQPGYYPTSPQQSGQL
QGQQPGQR
QGQQQPFPP
QGQQRQQPGQ
QGQSFGQPQQQVPVEIMRMVL
QGQSFSQPQQQVPVEIM
QGSFQPSQQNPQAQGSVQPQ
QGSLVQGQGI
QGSLVQGQGII
QGSVQPQQLPQ
QGTFLQPHQI
QHLARSQMWQQS
QHNIAH
QHNIVHG
QIIMRQQQGQSFGQPQQQVPV
QILGPL
QILQQIL
QILQQQ
QIPEQFRCQAIHNVAEAIRQQQPQQQW
QIPEQI
QIPEQSRCQAIHNVVHAI
QIPQQLQCAAIHSVA
QIPVDQPT
QIPVDQPT
QIPVVQ
QIPVVQP
QISEQAR
QISQQQQQPPFSQQQQPQFS
QIVQHQCCQQLAQIPER
QKETFPQQP
QKGTFLQPHQ
QLAHGTFL

QLAQGTF
QLAQGTFL
QLAQIPEQIRCHA
QLAQIPR
QLEAIRSLVLQTL
QLGQGQQGYYPTSPQ
QLGYYPTSPQQS
QLLQI
QLLQQQ
QLLRELCCQHL
QLMRQ
QLNPCKNFLL
QLNPCKVFLQQQCSPVAMPQRL
QLNPQAQGSVQPQQL
QLPQEQVPLVQQQQ
QLPQFEIRN
QLPYLQLQP
QLQCAAIHSVVHSI
QLQCTAI
QLQPFPQPQLPYSQP
QLQPFS
QLQPQNP
QLQPQNPSQ
QLQPQNPSQQL
QLQQPFPLQPQ
QLQQPLSQ
QLQQSILWGVPAL
QLRDVGPECQPV
QLSQIPEQYRCQAI
QLVLPQQ
QLVQQLC
QLVR
QMKPCAAFLQQKCSPLR
QMLQQSICHV
QNPQAQGSVQPQQL
QNPSQQQPQEQVPLVQEQQFQGQQQPFPPQQP
QPAQIPQ
QPEQLQQP
QPFLQPHQPFSQQPQQIFPQP
QPFPPQQPYPQP
QPFPQPQLPYSQPQPFRPQQ
QPFPQPQPFPP
QPFPQPQQTF
QPFPQQPQQS
QPFPQQPQQTYPQRPQQPFPQTQQPQQPFPQS
QPFPQTQQPQQP
QPFPSQLPYL
QPFPSQQPYPQPQP
QPFSQQPQQTFPQPQQTFPHQPQQQ
QPGQKQQSGQGQQ
QPGQRQP
QPGYYPTSPQQ

QPHQIARL
QPIPQQPQQPFPLQ
QPKNPSQQQPQE
QPLPPQQS
QPLQQLCCQ
QPLSQQPQQTFPQPQQTFPHQ
QPPFSQQQPILPQQ
QPPFSQQQPILS
QPPFSQQQQQPL
QPPFSQQQQQPLPQQP
QPPISQQQQPPFSQQQQPQFSQQQQIPV
QPPYSQQQQPPYSQQQQP
QPQEQVP
QPQEQVPLVQ
QPQFSQQQQP
QPQHQVP
QPQHQVPI
QPQHTFP
QPQPQ
QPQPQPQPQMNTC
QPQPQPQPQPQM
QPQQAFL
QPQQIFPQPQQ
QPQQLPQFEIRN
QPQQP
QPQQPAK
QPQQPAQLEVIRSLVLQTLPTMCNVYV
QPQQPFPQLQQPQ
QPQQPFPQQPQQP
QPQQPFPQSKQP
QPQQPFPQT
QPQQPFPQTQQSF
QPQQPFPWQPQQ
QPQQPISQQQAQ
QPQQPISQQQAQ
QPQQPISQQQAQQ
QPQQPQQPFPQPQ
QPQQPR
QPQQPT
QPQQQLGQQPQQQQVQKG
QPQQQSQQQLGQQPQQQQ
QPQQQSQQQLLQC
QPQQQVP
QPQQQVQSFSQPQHQ
QPQQSGQGVS
QPQQTYPQRP
QPQQVFPQPQQPQQQFPQPQQP
QPQYSQPQQPISQQQQQQQ
QPRQPFPQQPQQPYPQQPQQP
QPRQPFPQQPQQPYPQQPQQPFP
QPSQLNPQA
QPVGGGPVA

QPVLPPQQSPFPQQQQQHQ
QPVLPQQS
QPVPQPHQPF
QPYGSSLTSI
QPYLQLQP
QPYLQLQPFSQ
QPYPQLQPF
QPYPQPQPFPSQQPYLQLQPF
QQAFLQPQHTFP
QQCCQQLAQIPQ
QQCKPV
QQEQFPPQQPYPHQQP
QQEQLQGVQ
QQFIQPQQP
QQFIQPQQPFP
QQFLGQQ
QQFLQPQQP
QQFLQPQQPFP
QQFPGQQQPFP
QQFPGQQQPFPP
QQFPQPQQPQQSFPQQ
QQGQQQRQ
QQGQQSGQGQPRY
QQHNIVHGR
QQHSIAYGSSQVLQQSTY
QQILQQI
QQILQQQLT
QQIVV
QQKCSPLRMP
QQLAQGTFLQP
QQLAQIPQQLQCAAIHSVVHSTIMQQEQQEQLQ
QQLCCQQLFQIPEQSRCQAIHNVV
QQLGQCVSQPQQQLQQQ
QQLGQEQQDPQSGQG
QQLGQGQQPRQW
QQLGQQPQQQQVQKGTFLQPHQI
QQLIPC
QQLLQIPEQ
QQLNPCKNILLQQS
QQLNPCKV
QQLNPCKVFL
QQLPQFA
QQLPQFEEIRNLALQTLPAMCNV
QQLPQIPQQSRY
QQLQCAAIHSV
QQLQCAAIHSVVHSIIMQQEQQEQLQ
QQLQCAAIHTI
QQLQQQQQQQLQQQQQQQQQQQQP
QQLVQGQQGQ
QQPAFSQQQQT
QQPAQLEAIRSL
QQPFLQ

QQPFLQPH
QQPFLQPHQ
QQPFLQPQQPF
QQPFPQPQLPFPQQSEQIIPQQLQ
QQPFPQQPQQ
QQPFPQT
QQPFTQQQQPPFS
QQPGQGQ
QQPGQGQPGYY
QQPGQKQQSGQGQQGYYPISPQQL
QQPGQRQPG
QQPILSQQPP
QQPLFS
QQPLSQVSF
QQPPFI
QQPPFIQPSLQQQVN
QQPPFWQQQPP
QQPPISQQQQPPFSQ
QQPQP
QQPQQIFPQ
QQPQQIFPQPQQTFPHQPQQ
QQPQQPYPQQPQQ
QQPQQPYPQQPQQPFPQTQQPQQPF
QQPQQPYPQQQPYGSSLTSIG
QQPQQQFI
QQPQQQFL
QQPQQQQLAHG
QQPQQQQLAQGTFLQPHQI
QQPQQQQQQQVLQGTFLQ
QQPQQQQV
QQPTPI
QQPYLQL
QQPYLQLQPFS
QQPYMQLQPFPQ
QQPYPQLQPFPSQQPYLQ
QQPYPQQQPQYLQ
QQPYPQQQPYGSSLTSIG
QQQEQQILQQILQQQLTPCMD
QQQGFVQ
QQQGFVQP
QQQGFVQPQQQQPQQSVQGVY
QQQGIDIFL
QQQGQRFGQPQQQQGQSFGQP
QQQKQQQQQQQQI
QQQLFPQ
QQQLFPQQP
QQQLGQQPQQQQVQKGTF
QQQLPQPQQPQQ
QQQLQQQQQQQQQQQPSSQVSFQQPQQQYPSSQGS
QQQPFLQ
QQQPFPQPQQ
QQQPILSQ

QQQPILSQQPPFS
QQQPILSQQPPFSQQQQPVLPQQSPFS
QQQPLSQ
QQQPP
QQQPPFSQQQPILSQQPP
QQQPQEQVPLVQQQQFLGQQQPFPPQQPYPQP
QQQPQQQV
QQQPQQQWQGMYQPQQPAQLESI
QQQPSLIQ
QQQPVLPPQQSPFPQQQQQHQQLVQQQIP
QQQPVPQ
QQQQFPPQQPY
QQQQLGQQQQQQLQEQLTPCTT
QQQQLQEQLTPCTT
QQQQPAIQSFLQQQMNPCKNFLLQQCNHVSLV
QQQQPFTQQQQPPFSQQP
QQQQPGQRQLLEQMKPCAAFLQQKCSP
QQQQPGQRQLLEQMKPCVAFLQQQCR
QQQQPP
QQQQPQQLG
QQQQPSSQVSFQQPLQQ
QQQQQGIDIF
QQQQQGIDIFLPLSQHEQVGQGSLVQGQ
QQQQQPFTQQQQPPF
QQQQQPLSQVCF
QQQQQPPFTQQQQQQQ
QQQQQQLFPQ
QQQQQQLQEQLTP
QQQQQQPSSQVSFQQPQQ
QQQQQQQQPFTQQQQP
QQQQQQQQQQ
QQQQQQQQQQEQQIL
QQQQQQQQQQPLSQVS
QQQQQQQQQQQGQSIIQYQQQQPQQLG
QQQQQQQQTLQQ
QQQQTLQQI
QQQQVLQG
QQQRPFIQPS
QQQRQQQPSS
QQQRQQQPSSQVSLQQPQ
QQQSQQQLLQCSFQQPQQQLG
QQQVGQGI
QQQVQKGTFL
QQQYPLGQGSFR
QQRPKYL
QQRPMYLQPQQPISQQQAQQ
QQRQQPGQGQQ
QQSEQIIPQQLQQ
QQSGQKQPGYYPTSPW
QQSILW
QQSILWG
QQSLQQQLNPCKN

QQSPFPQQQQ
QQSQQSQQPF
QQTFPQQPLF
QQTYPHQPQQQ
QQTYPQRPQQP
QQVPVE
QQVPVEIMGM
QQVQGSIQSQQQQ
QQWQERQQQAQHKSM
QQWQERQQQAQHKSMRMLL
QRQQPGQGQ
QSFK
QSFPQQQPSLIQQSLQQQLNPC
QSIIQYQQQQPQQLGQCVSQPQQQ
QSKQPQQPFPQPQQ
QSTYQLLQQLCCQQLLQIPEQSQCQAI
QSTYQLVQQLC
QTFHHQPQ
QTFPHQPQQQFP
QTFPQPQQTFP
QTLPSMCNVNIPQ
QTPH
QTQQSFPLQP
QTVLPQQ
QTYPQR
QVEQLSSCQIDQ
QVIMRQQQGQ
QVLQQSTYQP
QVMDQQLRDI
QVMRQQCCQQPAQIPQQ
QVPLVQ
QVPWPQQQPFP
QVSFQPSQLN
QVVCFEINAERNERVWLAG
QWPQQQPFPQPQ
QYEQQIV
QYPSSQVSFQPSQLN
RCHAIHI
RCHAIHIVVE
RCHAIHSVVEAIMQQQSQQQWQER
RDTF
RDVVLQQH
RDVVLQQHS
RDVVLQQHSI
REAAEGGQGHRWPL
REEERGRGRG
RELKACQ
RELKACQQVMD
RESESEREHGKGRR
RESESEREHGKGRRREEEEDDQRQQ
RGDRGRGDEAVETFLRMATGA
RGQQGQQSGQGQQLGQGQQ

RGRGDEAVET
RHESIRA
RIYIPVQCPAPTAYNIPMV
RLEVM
RLEVMT
RLYEAD
RLYEADARSFHALANQD
RMPFLQ
RMSLQ
RMSLQALRS
RNLALETLPAM
RNNVIGKLGSPA
RPCVAFLQQQ
RPFIQPSLQ
RPLFQ
RPQQSYPQ
RPWQQQPLQQKET
RQGQQGYYP
RQGQQSGQRQ
RQLLEQM
RQLSQIPEQFRCQAIHNVAEAIRQQQ
RQPGYYSTSPQQLGQ
RQQQAQHKSM
RQQQAQHKSMRMLLE
RRRGDRGRG
RSIYIPVQCP
RSLVLQTLA
RSQML
RTTTSVPFGVGTGV
RTTTSVPFGVGTGVGA
RVDQPTSCQN
RYYLSVASPQQV
RYYPSV
RYYPSVTCPQQV
RYYPTCP
SAFVVPPGHPVVEISSSQGSSNLQVVCFEI
SEQARCQAVCSVSQIIM
SFGQPQQQV
SFQPQQLPQF
SFQQPLQQYPL
SFQQPLQQYPLGQGSFRPSQQNPQAQGSVQPQQLP
SFQQPQQQLGQ
SGQGFFQPSQQNPQAQGSVQPQ
SGQVPWPQQQPFPQPH
SGQVQWLQ
SIAHGSSQ
SIAHGSSQVLQQST
SIAPRTL
SIAYGSSQ
SIAYGSSQVLQQSTYQ
SIAYGSSQVLQQSTYQL
SIIMQ

SIIMQQ
SIIMQQE
SIIMQQEQQEQRQGVQ
SILQQLNPCKVF
SILQQLNPCKVFLQQCS
SIRMSLQALRSMCN
SIYIPVQCPAPTAYNIPMVATYTG
SKQPQQPF
SLQALRSMCS
SLQQQL
SLVSSLVS
SLWSIILPP
SLWSIIWPQS
SMRSIYIPVQCP
SPQQSGQLQQPAQGQQPG
SPQRSRDITSSSYHVSVEHQAASLKVAK
SPVAMPQHL
SQCQAIHNVVH
SQCQAIHNVVH
SQGSSNLQVVCFEI
SQKQQPV
SQLNPQAQGSVQ
SQPQLPYS
SQPQQPISQQQQQQQQQQQQQ
SQPQQQVPVEIMRMVL
SQPRQ
SQQPYLQLQPF
SQQPYMQLQPFPQP
SQQPYPQPQPFPP
SQQQAQ
SQQQLL
SQQQPILPQQP
SQQQQPPFSQQQQQPPFT
SQQQQPVL
SQQQQQPGQRQL
SQQQQQQQQQQQQQQQEQQILQQILQQQLI
SQTAYVQS
SQTAYVQSQMWQASGCQLMR
SQVLQEST
SQVLQQSTYQLVQQLCC
SQVLQQSTYQPLQQLC
SQVLQQSTYQPLQQLCCQQLWQIPEQSRCQAI
SQVSFQQPL
SQVSLQQP
SRRPYVFG
SSNLQVVCFEIN
SSQGSSNLQVVCFEINAERNER
SSQQNPQAQGSVQ
STINAPFA
STIRAPFASIVAG
STYQLLQELCCQHLWQIP
STYQLLRELCCQHLWQIPEQSQCQAIHNVVHA

SVAHSIIMQ
SVAHSIIMQQEQ
SVASPQQV
SVLQQLNPCKV
SVNIPQY
SVNIPQYCTTTPCSTITPAIYSIP
SVNVPLYETTTS
SVNVPLYSAT
SVNVPLYSATTSVRF
SVPQLQPQ
SVQPQQLP
SVQPQQLPQFAEIR
SVRFGVGTGVGAY
SVVEAIMQQ
SVVHSII
SVVHSTIMQ
SYHVSVEHQAASLKV
TAYNIPMVA
TAYVQS
TFPHQPQQQVP
TFPQPQQT
TIPQPHQ
TIPQPHQTFHHQPQQT
TITQTPYNI
TLPSMCNVNIP
TLPSMCSVNIPQYCTTTPCSTITPAIYS
TLVQGQ
TPAIYSIPMT
TPCRTITQTP
TPCSTIAPAI
TPCSTITPAIYS
TPCSTITPAIYSIPM
TPCTTFL
TPCTTFLQQQ
TQGQQGQQPGQGQQG
TQPFPP
TQPQQPTPIQ
TQQQQPPFSQQPP
TQQSFPL
TSAPLGVGSRVGA
TSAQQPGQLQ
TSCIPGLER
TSPLQPGQG
TTFLQQ
TTIAPFGIF
TTIAPFGIF
TTIAPFGIFGTN
TTPCSTIAPAI
TTPCSTITPAIYSIPMTAT
TTSVPFGVGTG
TTSVPFGVGTGVGA
TTSVPLGVGIGVGVY

TTTSVPFGVGTGVG
TTTSVPFGVGTGVG
TTTSVPFGVGTGVGA
TTTSVPFGVGTGVGA
TTTSVPFGVGTGVGAY
TTTSVPLGVGIGVG
TYQPLQQ
VAFLQQKCGP
VAFLQQKCSPLRMPFL
VAKAQQL
VAQIIM
VAVANI
VCGVAQVIM
VCGVAQVIMRQ
VCSVAQVIMR
VCSVSQIIMR
VDPSGQVPWPQQ
VEIMGMVLQT
VEQLSSCQIVQ
VEQLSSCQIVQH
VGQGIL
VGQGTLVQGQ
VHSII
VHSIIMQQEQQ
VHSIIMQQQQQQ
VHSIIMQQQQQQQQQGID
VHVP
VHVPPYCSTIRAPFAS
VLEGEGEVQIVC
VLPPQQQQQQL
VLPPQQQQQQLVQQQIP
VLQESTY
VLQQH
VLQQHNIVHG
VLQQHSIAH
VLQQLN
VLQQSSY
VLQQSSYQQLQQ
VLQQST
VLQQSTYQ
VLQQSTYQL
VLQTL
VLQTLATMC
VLQTLPLM
VLQTLPTMCNVYVPP
VMDQQLRDIS
VMRQQCC
VMRQQCCQQPAQI
VMTSIA
VNPRAF
VNVPLY
VNVPLYR

VNVPLYSS
VPFGVGTGVGAY
VPFPQI
VPGFTDADGVGY
VPLMQQ
VPLSQQQQVGQGT
VPLSQQQQVGQGTLVQGQG
VPLVQ
VPLVQQQQFLGQQQPFPPQQPYPQLQP
VPLYETTTSV
VPQLQLQ
VPVEIMGMVLQTLPSM
VPVEIMR
VPVPQL
VPVPQLQ
VPVPQLQLQN
VPVPQLQP
VPVPQLQPQNPSQQQPQEQ
VPVPQPQPQ
VPWPQQQP
VPYANIDAG
VQGQGIIQPQQPAQL
VQGQGIIQPQQPAQY
VQPQQSFP
VQPQQSFPQQSQQ
VQQLCCQQLWQI
VQQQIPVVQPSILQQ
VQQQIPVVQPSILQQ
VSLVSS
VSPVAGQY
VSQPQQQSQQQLGQQPQQQQLAQGTFLQPH
VSSLVS
VSSLWS
VSYYPGQASSR
VVCFEINAERN
VVDQQLRDVGP
VVHAIILHQQQQKQQQQPSSQFSFQQ
VVHAIIMQQE
VVLEGE
VVLQQH
VVLQQHNIAHGRS
VVLQQHNIAHGRS
VVLQQHSI
VVPPGHPVVE
VYQPQQQSQQQLLQ
WLQPGQW
WLQSGYYLTSPQQ
WPQQQPFL
WQIPEQSQ
WQIPEQSQ
WSIIWPQSD
WSIIWPQSDCQV

YEAIRAIIYS
YETTTSVPLGVG
YLQPQQP
YLSVASPQ
YLTSPQQSGQWQQPGQGQ
YPGETTPPQQLQQ
YPGQASSRR
YPISPQQLGQGQQSGQGQL
YPLGQGS
YPLGQGSFRPS
YPQPQQPISQQQ
YPQQPQRPF
YPQQRPMYLQPQQPISQQQAQQQQQQQQ
YPQRPQQPF
YPTSAQQPGQLQQ
YPTSPQQSGQKQPGYYPTSPWQPEQL
YQLLQE
YQLVQQLC
YQPLQQLCCQQL
YQPLQQLCCQQLWQIPEQSRCQAIHN
YQPQQPAQHESIRMSLQA
YRTTT
YRTTTSVPFGVGTGVGA
YRVAIMEV
YSTSPQQL
YTVKEGDVIVAPAGS
YVAQGEGVLTV
YVAQGEGVLTVIE
YVPIQCQQQQQLGQQQQQQLQ
YYPISPQQ

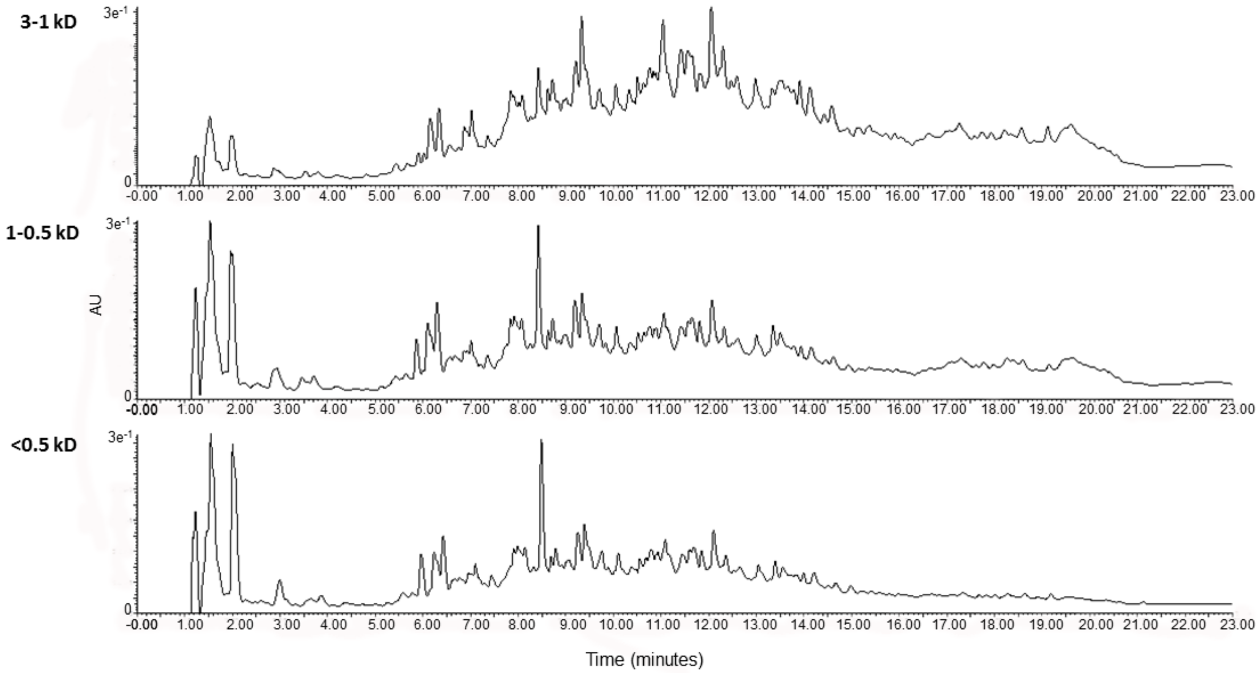

Supplementary file 6. UV chromatogram of the peptide fractions 3-1 kD, 1-0.5 kD and <0.5 kD of Wheat 1.
Specific peaks were increased in the TLR2 inhibiting fractions 1-0.5 kD and <0.5 kD compared to the not inhibiting 3-1 kD fraction, mainly at lower retention times. Also some new peaks are formed in these fractions.

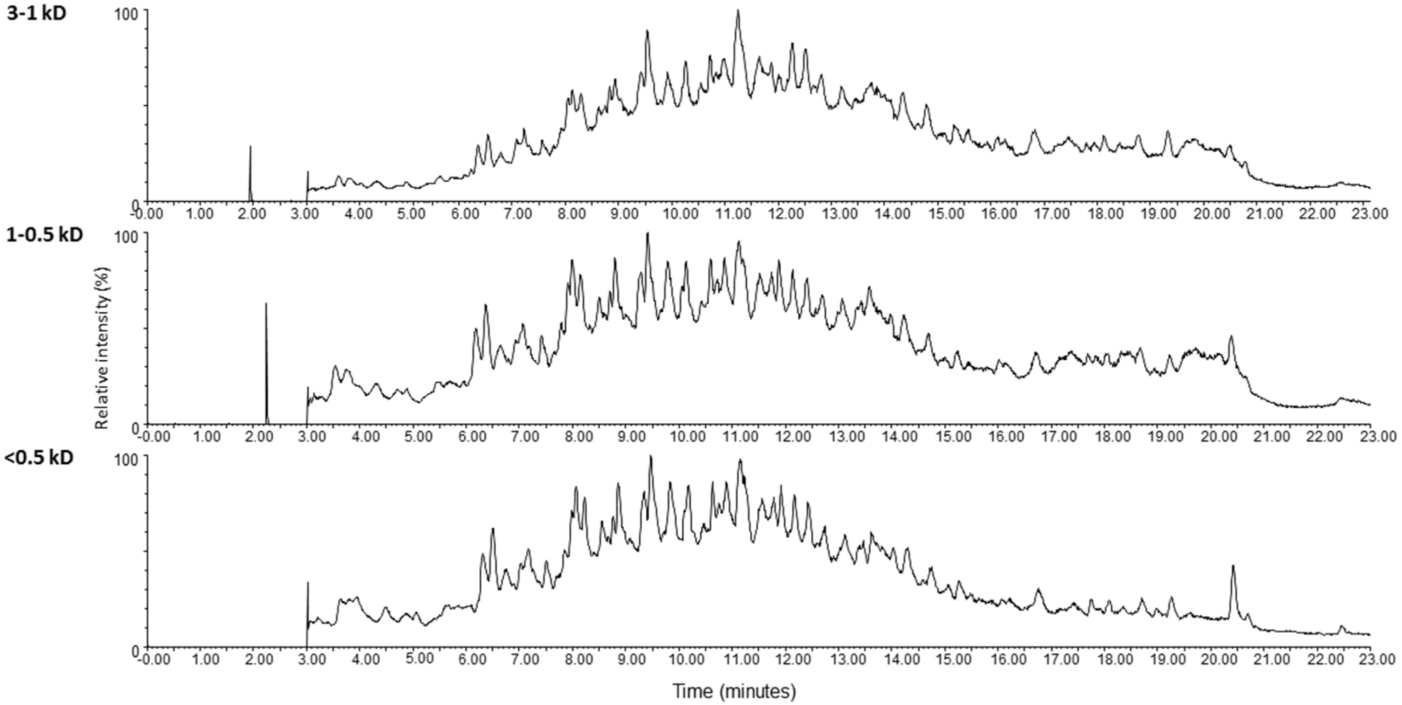

Supplementary file 7. Mass spectrum (total ion count) of the peptide fractions 3-1 kD, 1-0.5 kD and <0.5 kD of Wheat 1.
Relative intensity of several peaks was higher in the TLR2 inhibiting fractions 1-0.5 kD and <0.5 kD compared to the not inhibiting 3-1 kD fraction, and some new peaks are present in these fractions.
