# Supplementary material for: Identification of a TLR2 Inhibiting Wheat Hydrolysate
Source: Mol Nutr Food Res. 2018 Nov 2;62(23):1800716. doi: 10.1002/mnfr.201800716 (PMC6646915; doi:10.1002/mnfr.201800716)
Supplement: Supplementary file 2 — Supplementary [file MNFR-62-na-s002.docx]

**Reporter cell assay**

To test whether the wheat hydrolysates are able to inhibit TLR2, 4 and 9 activation induced by known ligands, the samples were tested on a HEK-XBlue^TM^-hTLR2, 4 and 9 (Invivogen, Toulouse, France) reporter cell assay.

For the assay, cells were seeded in a flat bottom 96 wells plate at a concentration described in table 1 (180 μL/well). Cells were stimulated with 2 mg/mL wheat hydrolysate, and the respective activating ligand (table 1) at the same time, and incubated for 24 hours (37 ̊C, 95% oxygen, 5% CO_2_). TLR ligand alone was used as a positive control. Medium was used as a negative control. After incubation, Quanti-Blue detection medium was added as described before [27]. Absorbance (650 nm) was quantified using a VersaMax microplate reader (Molecular Devices GmbH, Biberach an der Riss, Germany) and SoftMax Pro Data Acquisition & Analysis Software to determine SEAP activity, which represents activation of NF-κB/AP-1. The median and range for each sample were plotted as the fold-change compared to the positive control, which were TLR ligand stimulated cells. The positive controls were set at 1.

**Cell densities and ligands used in the different reporter cell line assays.**

| **Cell line** | **Cell density for seeding** | **Positive control (concentration in well)** |
| --- | --- | --- |
| HEK-Blue human TLR2 | 2.8*10^5^ cells/mL (180 µl/well) | Heat killed Listeria monocytogenes (10^7^ cells/mL)  P3CSK4 (25 ng/mL)  FSL-1 (25 ng/mL) |
| HEK-Blue human TLR4 | 1.4*10^5^ cells/mL (180 µl/well) | Escherichia coli K12 Lipopolysaccharide  (10 ng/mL) |
| HEK-Blue human TLR9 | 4.5*10^5^ cells/mL (180 µl/well) | Type B CpG oligonucleotide (ODN 2006, 0,25 μM) |

**Fractionation**

The hydrolysate was fractionated based on size using an Amicon stirred cell (Merck, Nottingham, UK) with a capacity of 50 mL. Filter membranes with MWCO’s of 3 kD (regenerated cellulose), 1 kD (regenerated cellulose) and 0.5 kD (cellulose acetate) (all Merck, Nottingham, UK) were used to prepare fractions containing peptides and proteins >3 kD, peptides between 3 and 1 kD, peptides between 1 and 0.5 kD and peptides <0.5 kD.Before use, the ultracentrifugal unit was sterilized, and the collecting tubes were cleaned with 70% ethanol. Filter membranes with MWCO’s of 3 kD (regenerated cellulose), 1 kD (regenerated cellulose) and 0.5 kD (cellulose acetate) (all Merck, Nottingham, UK) were used to prepare fractions containing peptides and proteins >3 kD, peptides between 3 and 1 kD, peptides between 1 and 0.5 kD and peptides <0.5 kD. To remove glycerine from the membranes before use, the membranes were soaked in sterile H­­_2_O for 1 hour.

Then, the hydrolysate was dissolved in 50 mL sterile water at a concentration of 40 mg/mL. To remove undissolved particles that could block the filtration membranes, the hydrolysate was centrifuged at 4000xg for 5 min, after which the supernatant was used for further processing. Two mL of the hydrolysate was stored at -20 ̊C. The rest was first filtered using the 3 kD filter under continuous stirring, by applying a N_2_ pressure (3,5 bar). The permeate was collected in a 50 mL tube, until approximately 80% of the sample was filtered. Then, the filtration was stopped, and the retentate was stirred for 15 min to remove proteins from the membrane. The retentate was also collected in a 50 mL tube. The described filtration steps were repeated for the collected 3 kD permeate using the 1 kD filter and for the collected 1 kD permeate using the 0.5 kD filter. Sterile water was filtered in the same manner to check for contamination during the filtration steps. The filtration process was performed 7 times and fractions were pooled in order to collect enough volume of the sample for subsequent experiments.

**RP-UHPLC**

In order to investigate which individual peptides could be responsible for TLR modulating effects, the peptide composition of the specific hydrolysate was fractionated and analyzed with RP-UHPLC coupled to MS.

To this end, wheat fraction samples (3-1 kD, 1-0.5 D and <0.5 kD) were diluted 4x with Mili-Q water. The diluted samples were centrifuged (16.110 g for 10 min), after which the supernatant was transferred to an HPLC vial. The obtained fractions were analyzed on an H class Acquity UPLC system (Waters, Milford, MA, USA) equipped with a BEH C18 column (1.7 μm, 2.1×100 mm, Waters) with an Acquity BEH C18 guard precolumn. The UPLC system was coupled to an Acquity 145 UPLC® PDA detector (Waters). Separation was carried out using the following elution profile at a flowrate of 0.350 mL/min: 5% ACN isocratic for 2 minutes; 5-42% ACN in 37 minutes; isocratic cleaning step of 90% ACN for the duration of 3 minutes; and re-equilibration to starting conditions for 6 minutes. Ultraviolet (UV) data was acquired using MassLynx software (Waters).

The mass spectra of the peptides were determined with Electron Spray Ionization Time of Flight Mass Spectrometry (ESI -Q-TOF-MS), using an online SYNAPT G2-Si high definition mass spectrometer (Waters) coupled to the RP-UHPLC. The system was calibrated with sodium iodide. The capillary voltage was set to 3 kV with the source operation in positive ion mode and the source temperature at 150 ̊C. The sample cone was operated at 40 V. Nitrogen was used as desolvation gas (500 ̊C, 800 L/h) and cone gas (200 L/h). MS and MS/MS (Resolution method) data were collected between m/z 100-3000 with a scan time of 0.3 seconds. Online lock mass data (Angiotensin II, Mw 523.7751 Da) were collected and the correction was applied during data reprocessing. The data were analyzed using Unifi software (Waters).
