# Supplementary material for: Identification of a TLR2 Inhibiting Wheat Hydrolysate
Source: Mol Nutr Food Res. 2018 Nov 2;62(23):1800716. doi: 10.1002/mnfr.201800716 (PMC6646915; doi:10.1002/mnfr.201800716)
Supplement: Supplementary file 3 — Supplementary [file MNFR-62-na-s003.docx]

**List of reference proteins**

Search strategy Uniprot: ‘triticum aestivum’+‘storage protein’ 🡪 72 hits

Excluded enzymes and unknown fragments

Glutenin, high molecular weight subunit DY10 EGEASRQLQCERELQESSLEACRQVVDQQLAGRLPWSTG

LQMRCCQQLRDVSAKCRSVAVSQVARQYEQTVVPPKGGSFYPGETTPLQQLQQGIFWGTS

SQTVQGYYPGVTSPRQGSYYPGQASPQQPGQGQQPGKWQEPGQGQQWYYPTSLQQPGQGQ

QIGKGQQGYYPTSLQQPGQGQQGYYPTSLQHTGQRQQPVQGQQPEQGQQPGQWQQGYYPT

SPQQLGQGQQPRQWQQSGQGQQGHYPTSLQQPGQGQQGHYLASQQQPGQGQQGHYPASQQQPGQGQQGHYPASQQQPGQGQQGHYPASQQEPGQGQQGQIPASQQQPGQGQQGHYPASLQQPGQGQQGHYPTSLQQLGQGQQTGQPGQKQQPGQGQQTGQGQQPEQEQQPGQGQQGYYPTSLQQPGQGQQQGQGQQGYYPTSLQQPGQGQQGHYPASLQQPGQGQPGQRQQPGQGQHPEQGKQPGQGQQGYYPTSPQQPGQGQQLGQGQQGYYPTSPQQPGQGQQPGQGQQGHCPTSPQQSGQAQQPGQGQQIGQVQQPGQGQQGYYPTSVQQPGQGQQSGQGQQSGQGHQPGQGQQSGQEQQGYDSPYHVSAEQQAASPMVAKAQQPATQLPTVCRMEGGDALSASQ

Glutenin, high molecular weight subunit DX5 EGEASEQLQCERELQELQERELKACQQVMDQQLRDISPE

CHPVVVSPVAGQYEQQIVVPPKGGSFYPGETTPPQQLQQRIFWGIPALLKRYYPSVTCPQ

QVSYYPGQASPQRPGQGQQPGQGQQGYYPTSPQQPGQWQQPEQGQPRYYPTSPQQSGQLQ

QPAQGQQPGQGQQGQQPGQGQPGYYPTSSQLQPGQLQQPAQGQQGQQPGQAQQGQQPGQGQQPGQGQQGQQPGQGQQPGQGQQGQQLGQGQQGYYPTSLQQSGQGQPGYYPTSLQQLGQGQSGYYPTSPQQPGQGQQPGQLQQPAQGQQPGQGQQGQQPGQGQQGQQPGQGQQPGQGQPGYYPTSPQQSGQGQPGYYPTSSQQPTQSQQPGQGQQGQQVGQGQQAQQPGQGQQPGQGQPGYYPTSPQQSGQGQPGYYLTSPQQSGQGQQPGQLQQSAQGQKGQQPGQGQQPGQGQQGQQPGQGQQGQQPGQGQPGYYPTSPQQSGQGQQPGQWQQPGQGQPGYYPTSPLQPGQGQPGYDPTSPQQPGQGQQPGQLQQPAQGQQGQQLAQGQQGQQPAQVQQGQRPAQGQQGQQPGQGQQGQQLGQGQQGQQPGQGQQGQQPAQGQQGQQPGQGQQGQQPGQGQQGQQPGQGQQPGQGQPWYYPTSPQESGQGQQPGQWQQPGQGQPGYYLTSPLQLGQGQQGYYPTSLQQPGQGQQPGQWQQSGQGQHWYYPTSPQLSGQGQRPGQWLQPGQGQQGYYPTSPQQPGQGQQLGQWLQPGQGQQGYYPTSLQQTGQGQQSGQGQQGYYSSYHVSVEHQAASLKVAKAQQLAAQLPAMCRLEGGDALSASQ

Avenin-like b1

QLETTCSQGFGQYQQQQQPGQRQLLEQMKPCVAFLQQQCRPL

RMPFLQTQVEQLSSCQIVQHQCCQQLAQIPERIRCHAIHSVVEAIMQQQSQQQWQERQQQ

AQHKSMRMLLENLSLMCNIYVPVQCQQQQQMGQQQQQQQLQEQLTPCATFLQHQCSPVTV

PFPQIPVDQPTSCQNVQHQCCRQLSQIPEQFRCQAIHNVAEAIRQQQPQQQWQGMYQPQQ

PAQHESIRMSLQALRSMCNIYIPVQCPAPTAYNIPMVATCTSGAC

Alpha/beta-gliadin

VRVPVPQLQPQNPSQQQPQEQVPLVQQQQFPGQQQPFPPQ

QPYPQPQPFPSQQPYLQLQPFPQPQLPYPQPQLPYPQPQLPYPQPQPFRPQQPYPQSQPQ

YSQPQQPISQQQQQQQQQQQQKQQQQQQQQILQQILQQQLIPCRDVVLQQHSIAYGSSQV

LQQSTYQLVQQLCCQQLWQIPEQSRCQAIHNVVHAIILHQQQQQQQQQQQQPLSQVSFQQ

PQQQYPSGQGSFQPSQQNPQAQGSVQPQQLPQFEEIRNLALETLPAMCNVYIPPYCTIAP

VGIFGTN

Glutenin, low molecular weight subunit 1D1 RCIPGLERPWQQQPLPPQQTFPQQPLFSQQQQQQLFP

QQPSFSQQQPPFWQQQPPFSQQQPILPQQPPFSQQQQLVLPQQPPFSQQQQPVLPPQQSP

FPQQQQQHQQLVQQQIPVVQPSILQQLNPCKVFLQQQCSPVAMPQRLARSQMLQQSSCHV

MQQQCCQQLPQIPQQSRYEAIRAIIYSIILQEQQQVQGSIQSQQQQPQQLGQCVSQPQQQ

SQQQLGQQPQQQQLAQGTFLQPHQIAQLEVMTSIALRILPTMCSVNVPLYRTTTSVPFGV

GTGVGAY

Glutenin, high molecular weight subunit 12

EGEASRQLQCERELQESSLEACRQVVDQQLAGRLPWSTG

LQMRCCQQLRDVSAKCRSVAVSQVARQYEQTVVPPKGGSFYPGETTPLQQLQQGIFWGTS

SQTVQGYYPSVTSPRQGSYYPGQASPQQPGQGQQPGKWQEPGQGQQWYYPTSLQQPGQGQ

QIGKGKQGYYPTSLQQPGQGQQIGQGQQGYYPTSPQHTGQRQQPVQGQQIGQGQQPEQGQ

QPGQWQQGYYPTSPQQLGQGQQPGQWQQSGQGQQGHYPTSLQQPGQGQQGHYLASQQQPAQGQQGHYPASQQQPGQGQQGHYPASQQQPGQGQQGHYPASQQEPGQGQQGQIPASQQQPGQGQQGHYPASLQQPGQQGHYPTSLQQLGQGQQIGQPGQKQQPGQGQQTGQGQQPEQEQQPGQGQQGYYPTSLQQPGQGQQQGQGQQGYYPTSLQQPGQGQQGHYPASLQQPGQGQGQPGQRQQPGQGQHPEQGQQPGQGQQGYYPTSPQQPGQGQQLGQGQQGYYPTSPQQPGQGQQPGQGQQGHCPMSPQQTGQAQQLGQGQQIGQVQQPGQGQQGYYPTSLQQPGQGQQSGQGQQSGQGHQPGQGQQSGQEKQGYDSPYHVSAEQQAASPMVAKAQQPATQLPTVCRMEGGDALSASQ

Glutenin, low molecular weight subunit PTDUCD1

METSCIPGLERPWQEQPLPPQHTLFPQQQPFPQQQQPPFS

QQQPSFLQQQPILPQLPFSQQQQPVLPQQSPFSQQQLVLPPQQQYQQVLQQQIPIVQPSV

LQQLNPCKVFLQQQCNPVAMPQRLARSQMLQQSSCHVMQQQCCQQLPQIPEQSRYDVIRA

ITYSIILQEQQQGFVQAQQQQPQQLGQGVSQSQQQSQQQLGQCSFQQPQQQLGQQPQQQQ

VLQGTFLQPHQIAHLEVMTSIALRTLPTMCSVNVPLYSSTTSVPFSVGTGVGAYL

Glutenin, high molecular weight subunit PW212 EGEASEQLQCERELQELQERELKACQQVMDQQLRDISPE

CHPVVVSPVAGQYEQQIVVPKGGSFYPGETTPPQQLQQRIFWGIPALLKRYYPSVTSPQQ

VSYYPGQASPQRPGQGQQPGQGQQSGQGQQGYYPTSPQQPGQWQQPEQGQPGYYPTSPQQ

PGQLQQPAQGQQPGQGQQGRQPGQGQPGYYPTSSQLQPGQLQQPAQGQQGQQPGQGQQGQQPGQGQQPGQGQQGQQPGQGQQPGQGQQGQQLGQGQQGYYPTSLQQSGQGQPGYYPTSLQQLGQGQSGYYPTSPQQPGQGQQPGQLQQPAQGQQPEQGQQGQQPGQGQQGQQPGQGQQPGQGQPGYYPTSPQQSGQGQPGYYPTSSQQPTQSQQPGQGQQGQQVGQGQQAQQPGQGQQPGQGQPGYYPTSPLQSGQGQPGYYLTSPQQSGQGQQPGQLQQSAQGQKGQQPGQGQQPGQGQQGQQPGQGQQGQQPGQGQPGYYPTSPQQSGQGQQPGQWQQPGQGQPGYYPTSPLQPGQGQPGYDPTSPQQPGQGQQPGQLQQPAQGQQGQQLAQGQQGQQPAQVQQGQQPAQGQQGQQLGQGQQGQQPGQGQQPAQGQQGQQPGQGQQGQQPGQGQQPGQGQPWYYPTSPQESGQGQQPGQWQQPGQWQQPGQGQPGYYLTSPLQLGQGQQGYYPTSLQQPGQGQQPGQWQQSGQGQHGYYPTSPQLSGQGQRPGQWLQPGQGQQGYYPTSPQQSGQGQQLGQWLQPGQGQQGYYPTSLQQTGQGQQSGQGQQGYYSSYHVSVEHQAASLKVAKAQQLAAQLPAMCRLEGGDALSASQ

Glutenin, low molecular weight subunit

QISQQQQAPPFSQQQQPPFSQQQQPPFSQQQQSPFSQQQQQ

PPFAQQQQPPFSQQPPISQQQQPPFSQQQQPQFSQQQQPPYSQQQQPPYSQQQQPPFSQQ

QQPPFSQQQQQPPFTQQQQQQQQQQPFTQQQQPPFSQQPPISQQQQPPFLQQQRPPFSRQ

QQIPVIHPSVLQQLNPCKVFLQQQCIPVAMQRCLARSQMLQQSICHVMQQQCCQQLRQIP

EQSRHESIRAIIYSIILQQQQQQQQQQQQQQGQSIIQYQQQQPQQLGQCVSQPLQQLQQQ

LGQQPQQQQLAHQIAQLEVMTSIALRTLPTMCNVNVPLYETTTSVPLGVGIGVGVY

Avenin-like a1

QLYTTCSQGYGQCQQQPQPQPQPQPQMNTCAAFLQQCSQTP

HVQTQMWQASGCQLVRQQCCQPLAQISEQARCQAVCSVAQIIMRQQQGQSFGQPQQQVPV

EIMRMVLQTLPLMCRVNIPQYCTTTPCSTITPAIYSIPMTATCAGGAC

Alpha/beta-gliadin

VRFPVPQLQPQNPSQQQPQEQVPLVQQQQFLGQQQPFPPQ

QPYPQPQPFPSQLPYLQLQPFPQPQLPYSQPQPFRPQQPYPQPQPQYSQPQQPISQQQQQ

QQQQQQQQQQQQQILQQILQQQLIPCMDVVLQQHNIAHGRSQVLQQSTYQLLQELCCQHL

WQIPEQSQCQAIHNVVHAIILHQQQKQQQQPSSQVSFQQPLQQYPLGQGSFRPSQQNPQA

QGSVQPQQLPQFEEIRNLALQTLPAMCNVYIPPYCTIAPFGIFGTN

Avenin-like a5

QLDTTCSQGYGQCQQQPQQQVNTCSALLQQCSPTPYVQSQM

WQASGCQLMRQQCCQPLAQISEQARCHAVCGVAQVIMRQQQGQSFGQPQQQQGQSFSQPQ

QQVPIEIRRMVLQTLPSMCNVNIPQYCTTTPCSTITQTPYNVPMATTCVGGTC

Alpha/beta-gliadin A-II

VRVPVPQLQLQNPSQQQPQEQVPLVQEQQFQGQQQPFPPQ

QPYPQPQPFPSQQPYLQLQPFPQPQLPYPQPQPFRPQQPYPQPQPQYSQPQQPISQQQQQ

QQQQQQQQQQILQQILQQQLIPCRDVVLQQHNIAHGSSQVLQESTYQLVQQLCCQQLWQI

PEQSRCQAIHNVVHAIILHQQHHHHQQQQQQQQQQPLSQVSFQQPQQQYPSGQGFFQPSQ

QNPQAQGSFQPQQLPQFEEIRNLALQTLPAMCNVYIPPYCTIAPFGIFGTN

Alpha/beta-gliadin A-III

VRVPVPQLQPQNPSQQQPQEQVPLMQQQQQFPGQQEQFPP

QQPYPHQQPFPSQQPYPQPQPFPPQLPYPQTQPFPPQQPYPQPQPQYPQPQQPISQQQAQ

QQQQQQQTLQQILQQQLIPCRDVVLQQHNIAHASSQVLQQSSYQQLQQLCCQQLFQIPEQ

SRCQAIHNVVHAIILHHHQQQQQQPSSQVSYQQPQEQYPSGQVSFQSSQQNPQAQGSVQP

QQLPQFQEIRNLALQTLPAMCNVYIPPYCSTTIAPFGIFGTN

Alpha/beta-gliadin A-IV

VRVPVPQLQPQNPSQQQPQKQVPLVQQQQFPGQQQPFPPQ

QPYPQQQPFPSQQPYMQLQPFPQPQLPYPQPQLPYPQPQPFRPQQSYPQPQPQYSQPQQP

ISQQQQQQQQQQQQQQQILQQILQQQLIPCRDVVLQQHSIAHGSSQVLQQSTYQLVQQFC

CQQLWQIPEQSRCQAIHNVVHAIILHQQQQQQQQQQQQQQQPLSQVCFQQSQQQYPSGQG

SFQPSQQNPQAQGSVQPQQLPQFEEIRNLALETLPAMCNVYIPPYCTIAPVGIFGTN

Alpha/beta-gliadin clone PW1215

VPVPQPQPQNPSQPQPQGQVPLVQQQQFPGQQQQFPPQ

QPYPQPQPFPSQQPYLQLQPFPQPQPFPPQLPYPQPPPFSPQQPYPQPQPQYPQPQQPIS

QQQAQQQQQQQQQQQQQQQQQQILQQILQQQLIPCRDVVLQQHNIAHARSQVLQQSTYQP

LQQLCCQQLWQIPEQSRCQAIHNVVHAIILHQQQRQQQPSSQVSLQQPQQQYPSGQGFFQ

PSQQNPQAQGSVQPQQLPQFEEIRNLALQTLPRMCNVYIPPYCSTTIAPFGIFGTN

Alpha/beta-gliadin A-V

VRVPVPQLQPQNPSQQQPQEQVPLVQQQQFPGQQQQFPPQ

QPYPQPQPFPSQQPYLQLQPFPQPQPFPPQLPYPQPQSFPPQQPYPQQQPQYLQPQQPIS

QQQAQQQQQQQQQQQQQQQILQQILQQQLIPCRDVVLQQHNIAHASSQVLQQSTYQLLQQ

LCCQQLLQIPEQSQCQAIHNVAHAIIMHQQQQQQQEQKQQLQQQQQQQQQLQQQQQQQQQQPSSQVSFQQPQQQYPSSQVSFQPSQLNPQAQGSVQPQQLPQFAEIRNLALQTLPAMCNV

YIPPHCSTTIAPFGISGTN

Alpha/beta-gliadin A-I

VRVPVPQLQPQNPSQQQPQEQVPLVQQQQFLGQQQPFPPQ

QPYPQPQPFPSQQPYLQLQPFLQPQLPYSQPQPFRPQQPYPQPQPQYSQPQQPISQQQQQ

QQQQQQQQQQQQQQIIQQILQQQLIPCMDVVLQQHNIVHGKSQVLQQSTYQLLQELCCQH

LWQIPEQSQCQAIHNVVHAIILHQQQKQQQQPSSQVSFQQPLQQYPLGQGSFRPSQQNPQ

AQGSVQPQQLPQFEEIRNLARK

Alpha/beta-gliadin clone PW8142

PVPQLQPKNPSQQQPQEQVPLVQQQQFPGQQQQFPPQQPY

PQPQPFPSQQPYLQLQPFPQPQPFLPQLPYPQPQSFPPQQPYPQQRPKYLQPQQPISQQQ

AQQQQQQQQQQQQQQQQQILQQILQQQLIPCRDVVLQQHNIAHASSQVLQQSTYQLLQQL

CCQQLLQIPEQSRCQAIHNVVHAIIMHQQEQQQQLQQQQQQQLQQQQQQQQQQQQPSSQV

SFQQPQQQYPSSQGSFQPSQQNPQAQGSVQPQQLPQFAEIRNLALQTLPAMCNVYIPPHC

STTIAPFGIFGTN

Avenin-like b5

QLETTCSQGFRQYQQQQQPGQRQLLEQMRPCVAFLQQQCRPL

RMPFLQTQVEQLSSCQIVQYQCCQQLAQIPEQIRCHAIHNVVEAIMQQQSQQQRQERQQQ

AQHKSMRMLLETLYLMCNIYVPIQCQQQQQLGQQQQQQLQEQLTPCATFLQHQCSPVTVP

FPQIPVDQPTSCQNVQHQCCRQLSQIPEQFRCQAIHNVAEAIRQQQPQQQWQGMYQPQQP

AQLESIRMSLQALRSMCSIYIPVQCPAPTAYNIPMVATYTGGAC

Avenin-like a2

QLYTTCSQGYGQCQQQPQPQPQPQPQMNTCAAFLQQCIQTP

YVQSQMWQASGCQLMRQQCCQPLAQISEQARCQAVCSVSQIIMRQQQGQRFGQPQQQQGQ

SFGQPQQQVPVEIMRMVLQTLPSMCSVNIPQYCTTTPCSTITPAIYSIPMTATCAGGAC

Avenin-like a3

QLYTTCSQGYGQCQQQPQPQPQMNTCAAFLQQCIQTPYVQS

QMWQASGCQLMRQQCCQPLAQISEQARCQAVCSVSQIIMRQQQGQRFGQPQQQQGQSFGQ

PQQQVPVEIMRMVLQTLPSMCSVNIPQYCTTTPCSTITPAIYSIPMTATCAGGAC

Avenin-like a4

QLDTTCSQGYGQCQQQPQQQVNTCSALLQQCSPTPYVQSQM

WQASGCQLMRQQCCQPLAQISEQARCQAVCSVAQVIMRQQQGQSFGQPQQQVQSFSQPQH

QVPIEITRMVLQTLPSMCNVNIPQYCTTTPCRTITQTPYNIPMSATCVGGTC

Avenin-like b10

QLETTCSQGFGQSQQQQQPGQRQLLEQMKPCVAFLQQKCSPL

RMPFLQTQVEQLSSCQIVQYQCCQQLAQIPERTRCHAIHIVVEAIIQQQSQQQWQEPQQQ

AQHKSMRMLLENLSLMCNIYVPVQCQQQQQLGQQQQQQLQEQLTPCTTFLQQQCSPVTVP

FPQIPVDQPTSCQNVQHQCCRQLSQIPEQFRCQAIHNVAEAIRQQQPQQQWQGMYQPQQP

AQLESIRMSLQALRSMRSIYIPVQCPAPTTYNIPLVATYTGGAC

Avenin-like b2

QLETTCSQGFGQSQQQQQPGQRQLLEQMKPCVAFLQQKCSPL

RMPFLQTQVEQLSSCQIVQYQCCQQLAQIPERTRCHAIHIVVEAIIQQQSQQQWQEPQQQ

AQHKSMRMLLENLSLMCNIYVPVQCQQQQQMGQQPQQQQLQEQLTPCATFLQHQCSPVTV

PFPQIPVDQPTSCQNVQHQCCRQLSQIPEQFRCQAIHNVAEAIRQQQPQQQWQGMYQPQQ

PAQHESIRMSLQALRSMCNIYIPVQCPAPTAYNIPMVATCTSGAC

Avenin-like b8

QLETTCSQGFGQSQQQQQPGQRQLLEQMKPCVAFLQQKCGPL

RMPFLQTQVEQLSSCQIVQYQCCQQLAQIPERTRCHAIHIVVEAIIQQQSQQQWQEPQQQ

AQHKSMRMLLENLSLMCNIYVPVQCQQQQQLGQQQQQQLQEQLTPCTTFLQQQCSPVTVP

FPQIPVDQPTSCQNVQHQCCRQLSQIPEQFRCQAIHNVAEAIRQQQPQQQWQGMYQPQQP

AQLESIRMSLQALRSMCSIYIPVQCPAPTTYNIPLVATYTGGAC

Avenin-like a6

QLYTTCSQGYGQCQQQPQPQPQPQPQPQMNTCSAFLQQCSQ

TAYVQSQMWQASGCQLMRQQCCQPLAQISEQARCQAVCSVAQIIMRQQQGQRFGQPQQQQ

GQSFSQPQQQVPVEIMGMVLQTLPSMCSVNIPQYCTTTPCSTIAPAIYNIPMTATCAGGA

C

Avenin-like b3

QLETTCSQGFGQSQQQQQPGQRQLLEQMKPCVAFLQQKCSPL

RMPFLQTQVEQLSSCQIVQYQCCQQLAQIPERTRCHAIHIVVEAIIQQQSQQQWQEPQQQ

AQHKSMRMLLENLSLMCNIYVPVQCQQQQQLGQQQQQQLQEQLTPCTTFLQQQCSPVTVP

FPQIPVDQPTSCQNVQHQCCRQLSQIPEQFRCQAIHNVAEAIRQQQPQQQWQGMYQPQQP

AQLESIRMSLQALRSMCNIYIPVQCPAPTTYNIPLVATYTGGAC

Avenin-like b6

QLETTCSQGFGQSQQQQQPGQRQLLEQMKPCVAFLQQKCSPL

RMPFLQTQVEQLSSCQIVQYQCCQQLAQIPERTRCHAIHIVVEAIIQQQSQQQWQEPQQQ

AQHKSMRMLLENLSLMCNIYVPVQCQQQQQLGQQQQQQLQEQLTPCTTFLQQQCSPVTVP

FPQIPVDQPTSCQNVQHQCCRQLSQIPEQFRCQAIHNVAEAIRQQQPQQQWQGMYQPQQP

AQLESIRMSLQALRSMCSIYIPVQCPAPTTYNIPLVATYTGGAC

Avenin-like a7

QLYTTCSQGYGQCQQQPQPQPQMNTCSAFLQQCIQTPYVQS

QMWQASSCQLMRQQCCQPLAQISEQARCQAVCSVSQIIMRQQQGQRFGQPQQQQGQSFSQ

PQQQVPVEIMRMVLQTLPSMCSVNIPQYCTTTPCSTITPAIYSIPMTATCAGGAC

Avenin-like b4

QLETTCSQGFRQYQQQQQPGQRQLLEQMRPCVAFLQQQCRPL

RMPFLQTQVEQLSSCQIVQYQCCQQLAQIPEQIRCHAIHNVVEAIMQQQSQQQRQERQQQ

AQHKSMRMLLENLSLMCNIYVPIQCQQQQQLGQQQQQQLQEQLTPCATFLQHQCSPVTVP

FPQIPVDQPTSCQNVQHQCCRQLSQIPEQFRCQAIHNVAEAIRQQQPQQQWQGMYQPQQP

AQLESIRMSLQALRSMCSIYIPVQCPAPTAYNIPMVATYTGGAC

Avenin-like b9

QLETTCSQGFGQSQQQQQPGQRQLLEQMKPCVAFLQQKCSPL

RMPFLQTQVEQLSSCQIVQYQCCQQLAQIPERTRCHAIHIVVEAIIQQQSQQQWQEPQQQ

AQHKSMRMLLENLSLMCNIYVPVQCQQQQQLGQQQQQQLQEQLTPCTTFLQQQCSPVTVP

FPQIPVDQPTSCQNVQYQCCRQLSQIPEQFRCQAIHNVAEAIRQQQPQQQWQGMYQPQQP

AQLESIRMSLQALRSMCSIYIPVQCPAPTTYNIPLVATYTGGAC

Avenin-like b11

QLDTTCSQGFRQYQQQQQPGQRQLLEQMRPCVAFLQQQCRPL

RMPFLQTQVEQLSSCQIDQYQCCQQLAQIPEQIRCHAIHNVVEAIMQQQSQQHRQERQQQ

AQHKSMRMLLETLYLMCNIYVPIQCQQQQQLGQQQQQQLQEQLTPCATFLQHQCSPVTVP

FPQIRVDQPTSCQNVQHQCCRQLSQIPEQYRCQAIHNVAEAIRHQQPQQQCQGMYQPQQP

AKLESIRMSLQALRSMCRIYIPVQCPAPTAYNIPMVATYTGGAC

Avenin-like b7

QLETTCSQGFGQSQQQQQPGQRQLLEQMKPCAAFLQQKCSPL

RMPFLQTQVEQLSSCQIVQYQCCQQLAQIPERTRCHAIHIVVEAIIQQQSQQQWQEPQQQ

AQHKSMRMLLENLSLMCNIYVPVQCQQQQQLGQQQQQQLQEQLTPCTTFLQQQCSPVTVP

FPQIPVDQPTSCQNVQHQCCRQLSQIPEQFRCQAIHNVAEAIRQQQPQQQWQGMYQPQQP

AQLESIRMSLQALRSMCSIYIPVQCPAPTTYNIPLVATYTGGAC

Gamma-gliadin

NIQVDPSGQVQWLQQQLVPQLQQPLSQQPQQTFPQPQQTFP

HQPQQQVPQPQQPQQPFLQPQQPFPQQPQQPFPQTQQPQQPFPQQPQQPFPQTQQPQQPF

PQQPQQPFPQTQQPQQPFPQLQQPQQPFPQPQQQLPQPQQPQQSFPQQQRPFIQPSLQQQ

LNPCKNILLQQSKPASLVSSLWSIIWPQSDCQVMRQQCCQQLAQIPQQLQCAAIHSVVHS

IIMQQQQQQQQQQGIDIFLPLSQHEQVGQGSLVQGQGIIQPQQPAQLEAIRSLVLQTLPS

MCNVYVPPECSIMRAPFASIVAGIGGQ

Gamma-gliadin B

NMQADPSGQVQWPQQQPFLQPHQPFSQQPQQIFPQPQQTFP

HQPQQQFPQPQQPQQQFLQPRQPFPQQPQQPYPQQPQQPFPQTQQPQQPFPQSKQPQQPF

PQPQQPQQSFPQQQPSLIQQSLQQQLNPCKNFLLQQCKPVSLVSSLWSIILPPSDCQVMR

QQCCQQLAQIPQQLQCAAIHSVVHSIIMQQEQQEQLQGVQILVPLSQQQQVGQGILVQGQ

GIIQPQQPAQLEVIRSLVLQTLPTMCNVYVPPYCSTIRAPFASIVASIGGQ

Gamma-gliadin B-I

SCISGLERPWQQQPLPPQQSFSQQPPFSQQQQQPLPQ

QPSFSQQQPPFSQQQPILSQQPPFSQQQQPVLPQQSPFSQQQQLVLPPQQQQQQLVQQQI

PIVQPSVLQQLNPCKVFLQQQCSPVAMPQRLARSQMWQQSSCHVMQQQCCQQLQQIPEQS

RYEAIRAIIYSIILQEQQQGFVQPQQQQPQQSGQGVSQSQQQSQQQLGQCSFQQPQQQLG

QQPQQQQQQQVLQGTFLQPHQIAHLEAVTSIALRTLPTMCSVNVPLYSATTSVPFGVGTG

VGAY

Gamma-gliadin

NMQVDPSGQVQWPQQQPFPQPQQPFCQQPQRTIPQPHQTFH

HQPQQTFPQPQQTYPHQPQQQFPQTQQPQQPFPQPQQTFPQQPQLPFPQQPQQPFPQPQQ

PQQPFPQSQQPQQPFPQPQQQFPQPQQPQQSFPQQQQPAIQSFLQQQMNPCKNFLLQQCN

HVSLVSSLVSIILPRSDCQVMQQQCCQQLAQIPQQLQCAAIHSVAHSIIMQQEQQQGVPI

LRPLFQLAQGLGIIQPQQPAQLEGIRSLVLKTLPTMCNVYVPPDCSTINVPYANIDAGIG

GQ

Gamma-gliadin

NIQVDPSGQVQWPQQQPFPQPQPFSQQPQQAFLQPQHTFPL

QPQQVFPQPQQPQQQFPQPQQPQQPFPQPQQPQLPFPQQPQQPFPQPQQPQQPFPQSQQP

QQPFPQPQQQFPQPQQPQQSFPQQQPPLIQPYLQQQMNPCKNYLLQQCNPVSLVSSLVSM

ILPRNDCQVMQQQCCQQLAQIPRQLQCTAIHSVVHAIIMQQEQQGIQILRPLFQLVQGQG

IIQPQQPAQYEVIRSLVLRTLPNMCNVYVRPDCSTINAPFASIVAGIGGQ

Low-molecular-weight glutenin storage protein

QISQQQQQPPFSQQQQPQFSQQPPFSQQQQPPFSQQQQQPP

FAQQQQPPFSQQPPISQQQQPPFSQQQQPQFSQQQQPPYSQQQQPPYSQQQQPPFSQQQQ

PPFSQQQQQPPFTQQQQQQQQQQPFTQQQQPPFSQQPPISQQQQPPFSQQQQPQFSQQQQ

IPVIHPSVLQQLNPCKVFLQQQCIPVAMQRCLARSQMLQQSICHVMQQQCCQQLRQIPEQ

SRHESIRAIIYSIILQQQQQQQQQQQQGQSIIQYQQQQPQQLGQCVSQPQQQLQQQLGQQ

PQQQQLAHGTFLQPHQIAQLEVMTSIAPRTLPTMCSVNVPLYETTTSVPLGVGIGVGVY

Gamma-gliadin

NMQVDPSGQVQWPQQQPFLQPHQPFSQQPQQIFPQPQQTFP

HQPQQQFPQPQQPRQQFLQPRQPFPQQPQQPYPQQPQRPFPQTQQPQQPFPQSKQPQQPF

PQPQQPQQSFPQQQPSLIQQSLQQQLNPCKDFLLQQCKPVSLVSSLWSIILPPSDCQVMR

QQCCQQLAQIPQQLQCAAIHSVVHSTIMQQEQQEQLQGVQILGPLSQQQQVGQGILVQGQ

GIIQPQQPAQLEVIGSLVLQTLPTMCNVHVPPYCSTIRAPFASIVASIGGQE

LMW-GS P-21

QMETRCIPGLERPWQQQPLPPQQTFPQQPLFSQQQQQQLFP

QQPSFSQQQPPFWQQQPPFSQQQPILPQQPPFSQQQQLVLPQQPPFSQQQQPVLPPQQSP

FPQQQQQHQQLVQQQIPVVQPSILQQLNPCKVFLQQQCSPVAMPQRLARSQMLQQSSCHV

MQQQCCQQLPQIPQQSRYEAIRAIIYSIILQEQQQVQGSIQSQQQQPQQLGQCVSQPQQQ

SQQQLGQQPQQQQLAQGTFLQPHQIAQLEVMTSIALRILPTMCSVNVPLYRTTTSVPFGV

GTGVGAY

Alpha gliadin

VRVSVPQLQPQNPSQQQPQEQVPLVQQQQFLGQQQPFPPQ

QPYPQLQPFPSQQPYLQLQPFPQPQLPYSQPQPFRPQQPYPQPQPQYSQPQQPISQQQQQ

QQQQQQQQQQQQQQQQILQQILQQQLIPCMDVVLQQHNIVHGRSQVLQQSTYQLLRELCC

QHLWQIPEQSQCQAIHNVVHAIILHQQQKQQQQPSSQVSFQQPLQQYPLGQGSFRPSQQN

PQAQGSVQPQQLPQFEEIRNLALQTLPAMCNVYIPPYCTIAPFGIFGTN

Alpha-gliadin

VRVPVPQLQPQNPSQQQPQEQVPLVQQQQFLGQQQPFPPQ

QPYPQPQPFPSQQPYLQLQPFLQPQLPYSQPQPFRPQQPYPQPQPQYSQPQQPISQQQQQ

QQQQQQQQQQQQQQIIQQILQQQLIPCMDVVLQQHNIVHGKSQVLQQSTYQLLQELCCQH

LWQIPEQSQCQAIHNVVHAIILHQQQKQQQQPSSQVSFQQPLQQYPLGQGSFRPSQQNPQ

AQGSVQPQQLPQFEEIRNLALQTLPAMCNVYIPPYCTIAPFGIFGTN

Omega gliadin storage protein

ARELNPSNKELQSPQQSFSYQQQPFPQQPYPQQPYPSQQPY

PSQQPFPTPQQQFPEQSQQPFTQPQQPTPIQPQQPFPQQPQQPQQPFPQPQQPFPWQPQQ

PFPQTQQSFPLQPQQPFPQQPQQPFPQPQLPFPQQSEQIIPQQLQQPFPLQPQQPFPQQP

QQPFPQPQQPIPVQPQQSFPQQSQQSQQPFAQPQQLFPELQQPIPQQPQQPFPLQPQQPF

PQQPQQPFPQQPQQSFPQQPQQPYPQQQPYGSSLTSIGGQ

Low-molecular-weight glutenin storage protein QMETRCIPGLERPWQQQPLPPQQTFPQQPLFSQQQQQQLFP

QQPSFSQQQPPFWQQQPPFSQQQPILPQQPPFSQQQQLVLPQQPPFSQQQQPVLPPQQSP

FPQQQQQHQQLVQQQIPVVQPSILQQLNPCKVFLQQCSPVAMPQRLARSQMLQQSSCHVM

QQQCCQQLPQIPQQSRYEAIRAIIYSIILQEQQQVQGSIQSQQQQPQQLGQCVSQPQQQS

QQQLGQQPQQQQLAQGTFLQPHQIAQLEVMTSIALRILPTMCSVNVPLYRTTTSVPFGVG

TGVGAY

High molecular weight glutenin subunit EGEASGQLQCERELQEHSLKACRQVVDQQLRDVGPECQP

VGGGPVARQYEQQVVVPPKGGSFYPGETTPPQQLQQSILWGVPALLRRYYLSVASPQQVS

YYPGQASSRRPGQGQQEYYLTSPQQSGQWQQPGQGQSGYYPTSPQQSGQKQPGYYPTSPW

QPEQLQQPTQGQQRQQPGQGQQLRQGQQGQQSGQGQPRYYPTSSQQPGQLQQLVQGQQGQQPERGQQGQQSGQGQQLGQGQQGQQPGQKQQSGQGQQGYYPISPQQLGQGQQSGQGQLGYYPTSPQQSGQGQSGYYPTSAQQPGQLQQSTQEQQLGQEQQDPQSGQGRQGQQSGQRQQDQQSGQGQQPGQRQPGYYSTSPQQLGQGQPRYYPTCPQQPGQEQQPRQLQQPEHGQQGQQPEQGQQGQQQRQGEHGQQPGQGQQGQQPGQGQPGYYPTSPQQSGQGQPGYYPTSPQQSGQLQQPAQGQQPGQEQQGQQPGQGQQPGQGQPGYYPTSPQQPGQEQQLEQWQQSGQGQPGHYPTSPLQPGQGQPGYYPTSPQQIGQGQQPGQLQQPTQGQQGQQPGQGQQGQQPGEGQQGQQPGQGQQPGQGQPGYYPTSLQQSGQGQQPGQWQQPGQGQPGYYPTSSLQPEQGQQGYYPTSQQQPGQGPQPGQWQQSGQGQQGYYPTSSQQSGQGQQPGQWLQPGQWLQSGYYLTSPQQLGQGQQPRQWSQPRQGQQGYYPTSPQQSGQGQQLGQGQQGYYPTSPQQSGQGQQGYDSPYYVSAEHQAASLKVAKAQQLAAQLPAMCRLEGGDALLASQ

Low-molecular-weight glutenin storage protein

QMETSCIPGLERPWQQQPLQQKETFPQQPPSSQQQQPFPQQ

PPFLQQQPSFSQQPLFSQKQQPVLPQQPAFSQQQQTVLPQQPAFSQQQHQQLLQQQIPIV

HPSILQQLNPCKVFLQQQCSPVAMPQHLARSQMWQQSSCNVMQQQCCQQLPRIPEQSRYE

AIRAIIFSIILQEQQQGFVQPQQQQPQQSVQGVYQPQQQSQQQLLQCSFQQPQQQLGQQP

QQQQVQKGTFLQPHQIARLEVMTSIALRTLPTMCSVNVPLYSSITSAPLGVGSRVGAY

Alpha-gliadin storage protein

VRFPVPQLQPQNPSQQLPQEQVPLVQQQQFLGQQQPFPPQ

QPYPQPQPFPSQLPYLQLQPFPQPQLPYSQPQPFRPQQPYPQPQPQYSQPQQPISQQQQQ

QQQQQQQQQQQQQILQQILQQQLIPCMDVVLQQHNIAHGRSQVLQQSTYQLLQELCCQHL

WQIPEQSQCQAIHNVVHAIILHQQQKQQQQPSSQVSFQQPLQQYPLGQGSFRPSQQNPQA

QGSVQPQQLPQFEEIRNLALQTLPAMCNVYIPPYCTIAPFGIFGTN

Low-molecular-weight glutenin storage protein QMETSCISGLERPWQQQPLPPQQSFSQQPPFSQQQQQPLPQ

QPSFSQQQPPFSQQQPILSQQPPFSQQQQPVLPQQSPFSQQQQLVLPPQQQQQQLVQQQI

PIVQPSVLQQLNPCKVFLQQQCSPVAMPQRLARSQMWQQSSCHVMQQQCCQQLQQIPEQS

RYEAIRAIIYSIILQEQQQGFVQPQQQQPQQSGQGLSQSQQQSQQQLGQCSFQQPQQQLG

QQPQQQQQQVLQGTFLQPHQIAHLEAVTSIALRTLPTMCSVNVPLYSATTSVRFGVGTGV

GAY

Alpha-/beta-gliadin storage protein VRVPVPQLQPQNPSQQQPQEQVPLVQQQQFPGQQQPFPPQ

QPYPQPQPFPSQQPYLQLQPFPQPQLPYPQPQPFRPQQPYPQPQPQYSQPQQPISQQQQQ

QQQQQQQQQILQQILQQQLIPCMDVVLQQHNIAQGRSQVLQQSTYQLLQELCCQHLWQIP

EQSQCQAIHNVVHAIILHQQHHHHQQQQQQQQQQPLSQVSFQQPQQQYPSGQGFFQPSQQ

NPQAQGSFQPQQLPQFEAIRNLALQTLPAMCNVYIPPYCTIAPFGIFGTN

Alpha-gliadin storage protein

VRWPVPQLQPQNPSQQQPQEQVPLVQQQQFLGQQQPFPPQ

QPYPQPQPFPSQQPYLQLQPFSQPQLPYSQPQPFRPQQPYPQPQPQYSQPQQPISQQQQQ

QQQQQQQQQQQQEQQILQQILQQQLTPCMDVVLQQHNIARGRSQVLQQSTYQLLQELCCQ

HLWQIPEKLQCQAIHNVVHAIILHQQQQKQQQPSSQVSFQQPQQQYPLGQGSFRPSQQNP

QAQGSVQPQQLPQFEEIRNLALQTLPAMCNVYIPPYCTIAPFGIFGTN

Alpha-/beta-gliadin storage protein

VRVPVPQLQPQNPSQQQPQEQVPLVQQQQFLGQQQPFPPQ

QPYPQPQPFPSQQPYLQLQPFPQPQLPYPQPQPFRPQQPYPQPQPQYSQPQQPISQQQQQ

QQQQQQQQQQILQQILQQQLIPCMDVVLQQHNIVHGRSQVLQQSTYQLLRELCCQHLWQI

PEQSQCQAIHNVVHAIILHQQQKQQQQPSSQVSFQQPLQQYPLGQGSFRPSQQNPQAQGS

VQPQQLPQFEEIRNLALQTLPAMCNVYIPPYCTIAPFGIFGTN

Storage protein

SHDDEDDRRGGHSLQQCVQRCRQERPRYSHARC

VQECRDDQQQHGRHEQEEEQGRGRGWHGEGEREEEHGRGRGRHGEGEREEEHGRGRGRHG

EGEREEERGRGHGRHGEGEREEERGRGRGRHGEGEREEEEGRGRGRRGEGERDEEQGDSR

RPYVFGPRSFRRIIQSDHGFVRALRPFDQVSRLLRGIRDYRVAIMEVNPRAFVVPGFTDA

DGVGYVAQGEGVLTVIENGEKRSYTVKEGDVIVAPAGSIMHLANTDGRRKLVIAKILHTI

SVPGKFQFLSVKPLLASLSKRVLRAAFKTSDERLERLFNQRQGQEKTRSVSIVRASEEQL

RELRREAAEGGQGHRWPLPPFRGDSRDTFNLLEQRPKIANRHGRLYEADARSFHALANQD

VRVAVANITPGSMTAPYLNTQSFKLAVVLEGEGEVQIVCPHLGRESESEREHGKGRRREE

EEDDQRQQRRRGSESESEEEEEQQRYETVRARVSRGSAFVVPPGHPVVEISSSQGSSNLQ

VVCFEINAERNERVWLAGRNNVIGKLGSPAQELTFGRPAREVQEVFRAQDQDEGFVAGPE

QQSREQEQEQERHRRRGDRGRGDEAVETFLRMATGAI

Seed storage protein

NMQVDPSGQVPWPQQQPFPQPHQPFSQQPQQTFPQPQQTFP

HQPQQQFSQPQQPQQQFIQPQQPFPQQPQQTYPQRPQQPFPQTQQPQQPFPQSQQPQQPF

PQPQQQFPQPQQPQQSFPQQQPSLIQQSLQQQLNPCKNFLLQQCKPVSLVSSLWSMILPR

SDCQVMRQQCCQQPAQIPQQLQCAAIHSIVHSIIMQQEQQEQRQGVQILVPLSQQQQVGQ

GTLVQGQGIIQPQQPAQLEVIRSLVLQTLATMCNVYVPPYCSTIRAPFASIVAGIGGQ

High-molecular-weight glutenin EGEASEQLQCDRELQELQERELKACQQVMDQQLRDISPECHPVVVSPVAGQYEQQIVVP

PKGGTFYPGETTPPQQLQQRIFWGIPALLKRYYPSVTCPQQVSYYPGQASPQRSRDITSS

SYHVSVEHQAASLKVAKAQQLAAQLPAMCRLEGGDALSASQ

Alpha/beta-gliadin storage protein VRVPVPQLQPKNPSQQQPQEQVPLVQQQQFPGQQQQFPPQQPY

PQPQPFPSQQPYLQLQPFPQPQPFLPQLPYPQPQSFPPQQPYPQQRPMYLQPQQPISQQQ

AQQQQQQQQQQQQQQQQQILQQILQQQLIPCRDVVLQQHNIAHASSQVLQQSTYQLLQQL

CCQQLLQIPEQSRCQAIHNVVHAIIMHQQEQQQQLQQQQQQQLQQQQQQQQQQQQPSSQV

SFQQPQQQYPSSQGSFQPSQQNPQAQGSVQPQQLPQFAEIRNLALQTLPAMCNVYIPPHC

STTIAPFGIFGTN

Alpha-gliadin storage protein

VRVPVPQLQPKNPSQQQPQEQVPLVQQQQFPGQQQQFPPQQPY

PQPQPFPSQQPYLQLQPFPQPQPFLPQLPYPQPQSFPPQQPYPQQRPKYLQPQQPISQQQ

AQQQQQQQQQQQQQQQQQILQQILQQQLIPCRDVVLQQHNIAHASSQVLQQSTYQLLQQL

CCQQLLQIPEQSRCQAIHNVVHAIIMHQQEQQQQLQQQQQQQLQQQQQQQQQQQQPSSQV

SFQQPQQQYPSSQVSFQPSQLNPQAQGSVQPQQLPQFAEIRNLALQTLPAMCNVYIPPHC

STTIAPFGIFGTN
