# Supplementary figures and images for: Identification of a TLR2 Inhibiting Wheat Hydrolysate
Source: Mol Nutr Food Res. 2018 Nov 2;62(23):1800716. doi: 10.1002/mnfr.201800716 (PMC6646915; doi:10.1002/mnfr.201800716)

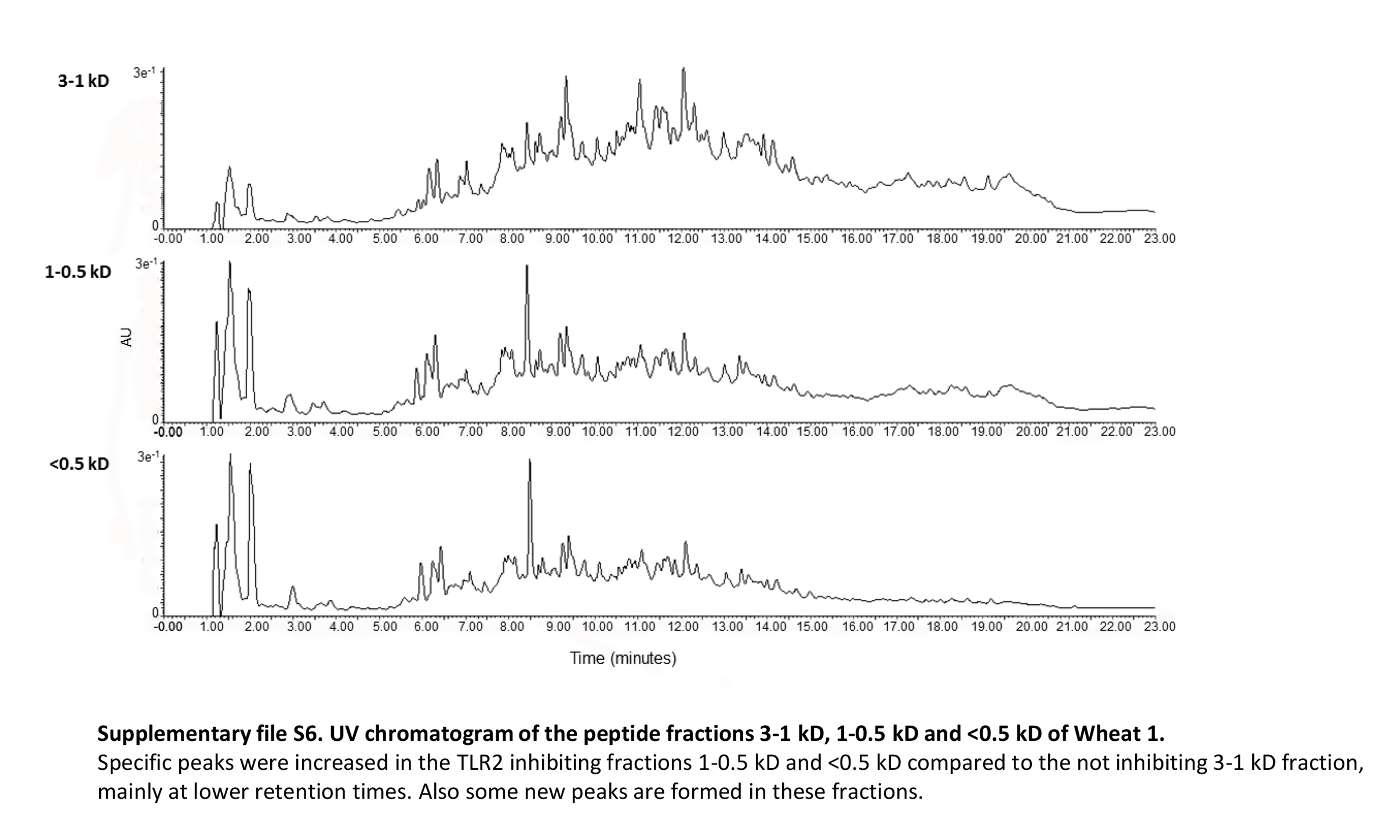

Supplement: Supplementary file 6 — Supplementary [file MNFR-62-na-s006.tif]

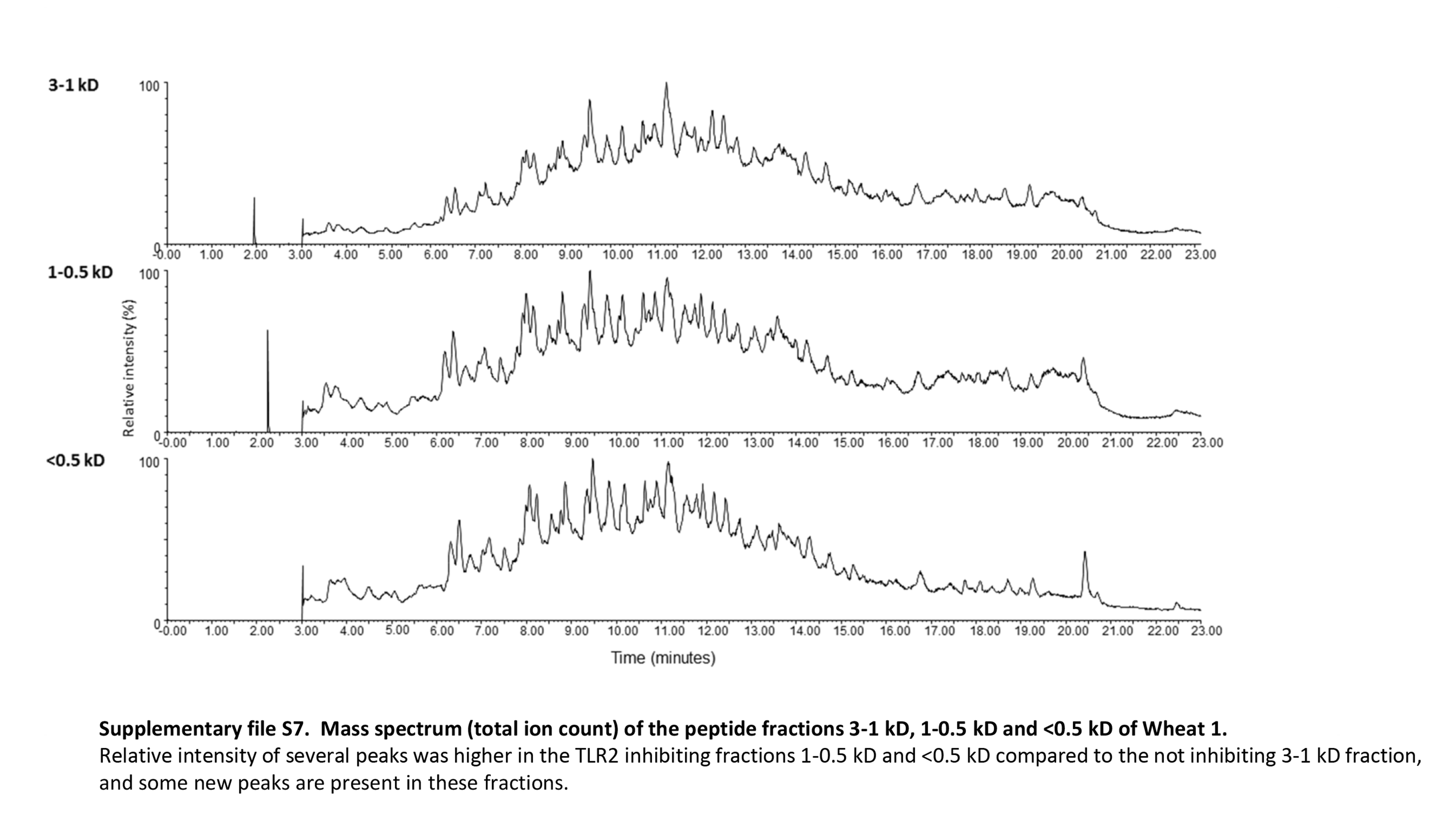

Supplement: Supplementary file 7 — Supplementary [file MNFR-62-na-s007.tif]
